# Mutant CHCHD10 disrupts cytochrome *c* oxidation and activates mitochondrial retrograde signaling

**DOI:** 10.1038/s44321-025-00358-5

**Published:** 2025-12-19

**Authors:** Márcio Augusto Campos-Ribeiro, Erminia Donnarumma, Hendrik Nolte, Paul Cobine, Elodie Vimont, Dusanka Milenkovic, Juan Diego Hernandez-Camacho, Francina Langa-Vives, Etienne Kornobis, Esthel Pénard, Sonny Yde, Thomas Langer, Véronique Paquis-Flucklinger, Timothy Wai

**Affiliations:** 1 Institut Pasteur, CNRS UMR 3691, Mitochondrial Biology Unit, Université Paris Cité, Paris, 25-28 Rue du Docteur Roux, 75015 France; 2 Max-Planck-Institute for Biology of Ageing, Department of Mitochondrial Proteostasis, Joseph-Stelzmann-Str. 9b, Cologne, 50931 Germany; 3 Department of Biological Sciences, Auburn University, Auburn, AL USA; 4 Institut Pasteur, Mouse Genetics Engineering Center, Université Paris Cité, Paris, 25-28 Rue du Docteur Roux, 75015 France; 5 Institut Pasteur, Biomics core facility, Bioinformatics and Biostatistics Hub, Université Paris Cité, Paris, 25-28 Rue du Docteur Roux, 75015 France; 6 Institut Pasteur, Ultrastructural Bioimaging Core Facility, Université Paris Cité, Paris, 25-28 Rue du Docteur Roux, 75015 France; 7 Université Côte d’Azur (UniCA), Inserm U1081, CNRS UMR7284, Institute for Research and Aging (IRCAN), Mitochondria, Disease and Aging Team, Nice, France; 8 Department of Medical Genetics, Reference Centre for Mitochondrial Diseases, Centre Hospitalier Universitaire (CHU) de Nice, Nice, France

**Keywords:** Mitochondrial Disease, CHCHD10, OMA1, Cytochrome c, Cardiomyopathy, Cardiovascular System, Metabolism

## Abstract

Mutations in *CHCHD10*, a mitochondrial intermembrane space (IMS) protein implicated in proteostasis and cristae maintenance, cause mitochondrial disease. Knock-in mice modeling the human *CHCHD10*^*S59L*^ variant associated with ALS–FTD develop a mitochondrial cardiomyopathy driven by CHCHD10 aggregation and activation of the mitochondrial integrated stress response (mtISR). We show that cardiac dysfunction is associated with dual defects originating at the onset of disease: (1) bioenergetic failure linked to impaired mitochondrial copper homeostasis and cytochrome c oxidation, and (2) maladaptive mtISR signaling via the OMA1-DELE1-HRI axis. Using protease-inactive *Oma1*^*E324Q/E324Q*^ knock-in mice, we show that blunting mtISR in *Chchd10*^*S55L/+*^ mice delays cardiomyopathy onset without rescuing CHCHD10 insolubility, cristae defects or OXPHOS impairment. Proteomic profiling of insoluble mitochondrial proteins in *Chchd10*^*S55L/+*^ mice reveals widespread disruptions of mitochondrial proteostasis, including IMS proteins involved in cytochrome *c* biogenesis. Defective respiration in mutant mitochondria is rescued by the addition of cytochrome *c*, pinpointing IMS proteostasis disruption as a key pathogenic mechanism. Thus, mutant CHCHD10 insolubility compromises metabolic resilience by impairing bioenergetics and stress adaptation, offering new perspectives for the development of therapeutic targets.

## Synopsis

Characterization of *Chchd10*^*S55L*^ mouse model of mitochondrial disease reveals that mutant CHCHD10 protein aggregation triggers a primary bioenergetic deficiency and integrated stress response activation in a tissue-specific manner, which can be uncoupled by crossing with *Oma1*^*E324Q/E324Q*^ mice.

- Genetic inhibition of OMA1 suppressed the ISR and delayed cardiomyopathy in *Chchd10*^*S55L*^ mice, despite persistent CHCHD10 insolubility and respiratory chain defects.
- CHCHD10 insolubility caused secondary defects in cytochrome *c* and copper homeostasis.
- Exogenous cytochrome c supplementation rescued impaired respiration in mutant mitochondria, pinpointing disrupted cytochrome *c* biology as a therapeutic vulnerability.
- CHCHD10 insolubility was established as a primary pathogenic trigger linking mitochondrial proteostasis failure to cardiomyopathy progression.

## Abstract (web summary)

Characterization of *Chchd10*^*S55L*^ mouse model of mitochondrial disease reveals that mutant CHCHD10 protein aggregation triggers a primary bioenergetic deficiency and integrated stress response activation in a tissue-specific manner, which can be uncoupled by crossing with *Oma1*^*E324Q/E324Q*^ mice.

## Graphical abstract

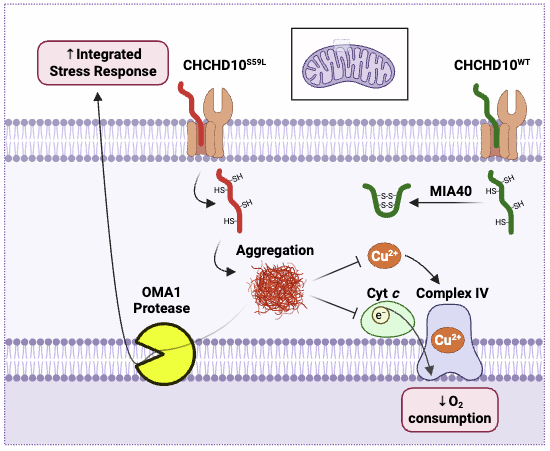

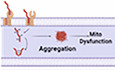

The paper explained **Problem**

Mutations in the mitochondrial protein CHCHD10 cause multisystemic mitochondrial diseases. The dominantly inherited S59L mutation, linked to ALS–FTD, has an unclear mechanism, with debate over whether bioenergetic failure or maladaptive stress signaling drives pathology. The relationship between CHCHD10 insolubility, mitochondrial stress responses, and respiratory chain defects remains unresolved.

**Results**

Using a knock-in *Chchd10*^*S55L*^ mouse model, we show that catalytic inactivation of OMA1 suppressed the mitochondrial stress response and delayed cardiomyopathy, without correcting CHCHD10 insolubility, cristae disruption, or respiratory defects. Knock-on effects of mutant CHCHD10 protein insolubility uncovered by proteomics revealed cytochrome *c* and copper homeostasis defects. Supplementation of exogenous cytochrome c rescued defective respiration in mutant mitochondria.

**Impact**

These findings identify CHCHD10 insolubility as a pathogenic trigger that impairs both energy metabolism and stress signaling. They highlight intermembrane space proteostasis and cytochrome *c* biology as therapeutic opportunities for mitochondrial cardiomyopathies and related disorders.

## Introduction

Mitochondria are multi-functional organelles that execute essential biosynthetic and signaling functions that govern the life and death of the cell (Monzel et al, 2023). Inherited defects in mitochondria cause Mitochondrial Diseases (MD), which are rare, clinically heterogeneous, and multisystemic disorders caused by pathogenic variants in genes that code for ~400 out of the >1200 mitochondrial proteins that have been identified to date (Russell et al, 2020; Rath et al, 2021). The tissue-specific nature of MD has long been proposed to reflect the differential bioenergetic and metabolic requirements and tissue-specific biochemical thresholds (Wallace et al, 2010). More recent studies exploring the functional impact of downstream stress signaling triggered by mitochondrial dysfunction have highlighted the physiological relevance of cell signaling for disease etiology, trajectory, and severity (Picard and Shirihai, 2022; Lepelley et al, 2021). The mitochondrial integrated stress response (mtISR) (Lehtonen et al, 2016) depends on the proteolytic cleavage and maturation of DAP3 Binding Cell Death Enhancer 1 (DELE1) within mitochondria by the stress-induced mitochondrial protease OMA1 (Guo et al, 2020; Fessler et al, 2020), which enables translocation of DELE1 to the cytosol where it activates the heme-regulated inhibitor kinase HRI, leading to cytosolic translational attenuation and selective upregulation of stress-responsive genes encoding chaperones, transport proteins, and proteases by Activating Transcription Factors like ATF4 (Ng et al, 2021).

CHCHD10 is a small 14 kDa protein that belongs to the family of CHCHD proteins, which are characterized by the presence of a CHCH domain, which consists of two helix-coil-helix motifs connected by a loop. The twin Cx(9)C motif (C-X₉-C-X₂-C-X₉-C) contains four conserved cysteines that are required for intramolecular disulfide bridges that are critical for protein stability and function. CHCHD proteins are translated on cytosolic ribosomes and imported into the intermembrane space (IMS) via the disulfide relay system catalyzed by the copper- and zinc-dependent protein (encoded by *CHCHD4*) and the Mitochondrial FAD-linked sulfhydryl oxidase ERV1 (encoded by *GFER*) (Dickson-Murray et al, 2021). MIA40 forms a redox cycle with cysteine motif-containing client proteins destined for IMS import, which are initially translocated in a reduced state. Upon entry, the oxidized form of MIA40 forms a transient intermolecular disulfide intermediate with the reduced precursor, facilitating the transfer of disulfide bonds. Electrons passed from MIA40 to ERV1, which reduces FAD to FADH_2_, are then passed on to cytochrome *c* or molecular oxygen (O_2_) to complete the redox cycle. Defects in the MIA40/ERV1 import pathway can impinge on the biogenesis of client proteins, which play roles in various intramitochondrial processes, including the assembly of enzymes of the electron transport chain (ETC), maintenance of the MICOS complex and cristae morphology, and mitochondrial proteostasis (Dickson-Murray et al, 2021). Inborn errors in ERV1/GFER are associated with multisystemic MD characterized by combined respiratory chain deficiency and disordered cristae (Di Fonzo et al, 2009; Nambot et al, 2017). Pathogenic variants in the mitochondrial protein coiled-coil-helix-coiled-coil-helix domain-containing protein 10 (CHCHD10) were initially linked to autosomal dominant (AD) amyotrophic lateral sclerosis and frontotemporal dementia (ALS–FTD) (Bannwarth et al, 2014; Johnson et al, 2014; Müller et al, 2014) and later studies identified mutations in patients suffering from Charcot-Marie-Tooth neuropathy (Auranen et al, 2015), spinal muscular atrophy (Penttilä et al, 2015), mitochondrial myopathy, and cardiomyopathy (Shammas et al, 2022). Transgenic mouse models for these pathogenic variants recapitulate some of the cellular dysfunctions described in patients, yet there are conflicting hypotheses regarding the precise molecular defects underscoring disease onset (Genin et al, 2022; Sayles et al, 2022; Lin et al, 2024; Baek et al, 2021).

Toxic gain-of-function mechanisms underlying the expressivity of the AD *CHCHD10* variant S59L (OMIM: #615911) promote CHCHD10 insolubility, aggregation, and accumulation, consistent with atomic-level structural studies suggesting a greater propensity for oligomerization (Alici et al, 2022; Lv et al, 2025). For CHCHD10^S59L^, insolubility occurs within the IMS and promotes the accumulation and insolubility of its interaction partner CHCHD2 in both patient-derived cells and mouse models (Shammas et al, 2022; Anderson et al, 2019; Genin et al, 2019, 2016; Southwell et al, 2024). In *CHCHD10*^*S59L/+*^ patients, muscle biopsies showed ragged-red fibers, cytochrome *c* oxidase (COX)-negative fibers, and mitochondrial DNA (mtDNA) deletions that have been proposed to result from mtISR-dependent nucleotide imbalance (Sayles et al, 2022). Heterozygous *Chchd10*^*S55L/+*^ knock-in mice (carrying the S59L murine equivalent) develop a tissue-specific MD characterized by an early-onset cardiomyopathy and inability to gain weight, followed by a late-onset neuromuscular decline and death around 1 year of age. In these animals, CHCHD10 accumulates and aggregates in affected tissues, leading to mtISR induction and culminating in extensive proteomic, morphological, and ultrastructural remodeling of mitochondria (Anderson et al, 2019; Genin et al, 2019; Sayles et al, 2022; Shammas et al, 2023). The prevailing model proposes that mtISR induction triggers progressive metabolic and late-stage OXPHOS decline in *Chchd10*^*S55L/+*^ knock-in mice through the remodeling of pathways governed by proteins encoded by ISR target genes (Sayles et al, 2022). Efforts to blunt the mtISR through the whole-body *Oma1* deletion in *Chchd10*^*G54R/+*^ knock-in mice, which model an AD mitochondrial myopathy caused by the G58R gain-of-function variant, revealed OMA1 and mutant CHCHD10 to be synthetically lethal (Shammas et al, 2022). In this model, tissue-specific ablation of *Oma1* and/or *Dele1* exacerbated myopathy, leading to the opposite conclusion that the mtISR is required for tissue homeostasis (Lin et al, 2024), at least in skeletal muscle. Hence, the functional impact of the mtISR remains controversial, with its inhibition in models of mitochondrial dysfunction beyond CHCHD10 reported to be both protective and maladaptive in vivo (Han et al, 2023; Ahola et al, 2022; Croon et al, 2022; Vela-Sebastián et al, 2024; Kaspar et al, 2021; Jackson et al, 2025).

In this study, we set out to characterize the relationship between CHCHD10 protein insolubility caused by the S59L mutation, the mtISR signaling pathway, and mitochondrial bioenergetics in the heart. We confirmed that CHCHD10 protein insolubility and mtISR induction precede cardiac dysfunction and are therefore a candidate trigger for disease onset in *Chchd10*^*S55L/+*^ mice. To determine the relevance of the mtISR, we introduced the catalytically-inactivating mutation E324Q in the *Oma1* gene of *Chchd10*^*S55L/+*^ mice, which blunted mtISR signaling and delayed cardiomyopathy without rescuing early-onset defects in CHCHD10 insolubility, cristae structure, cytochrome *c* oxidation, or mtDNA depletion. Biochemical and OMICs-based studies of cardiac mitochondria from *Chchd10*^*S55L/+*^ mice revealed defects in cytochrome *c* oxidation capacity, mitochondrial copper levels, and mitochondrial respiration that manifest at or before disease onset, which is in contrast with previous studies proposing OXPHOS defect to be a late-stage consequence of ISR-dependent rewiring of mitochondria (Sayles et al, 2022; Southwell et al, 2024). Importantly, in vitro supplementation of exogenous cytochrome *c* was able to rescue impaired respiration in mitoplasts from *CHCHD10*^*S55L/+*^ hearts, which were found to be deficient in cytochrome *c* and associated biogenesis factors that rely on the MIA40/ERV1 disulfide relay for import into the IMS. Taken together, our results reveal the contribution of impaired bioenergetics and mtISR signaling for mitochondrial homeostasis and cardiac health, which are compromised by CHCHD10 insolubility.

## Results

### CHCHD10 insolubility triggers ISR activation and cardiac remodeling in *Chchd10* mice

**Figure 1:**
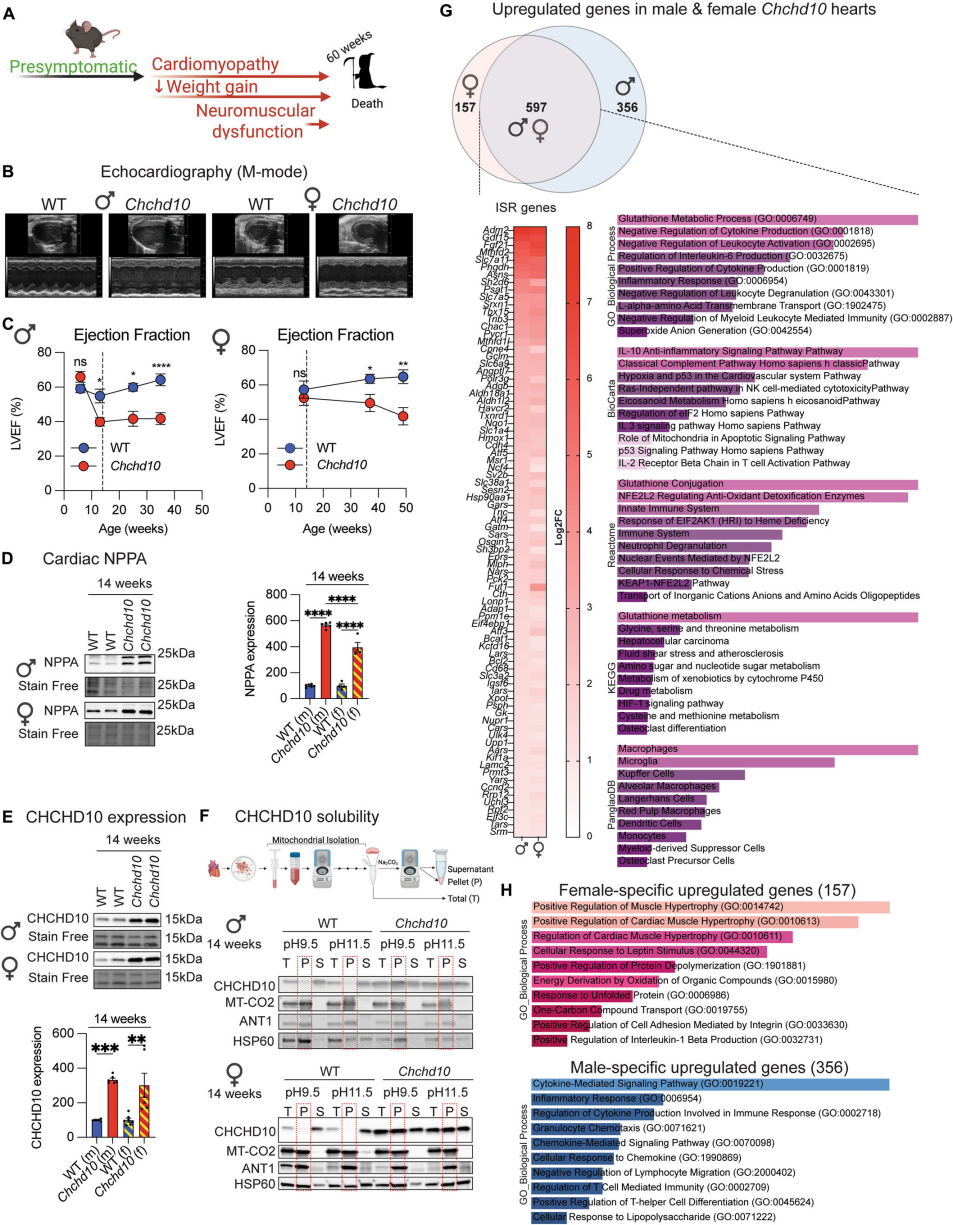
CHCHD10 insolubility triggers ISR activation and cardiac remodeling. (**A**) *Chchd10* heterozygous missense Ser55Leu (S55L) mutation causes tissue-specific defects in mice and reduced lifespan in *Chchd10*^*S55L/+*^ mutant mice. The presymptomatic–symptomatic transition to cardiomyopathy precedes neuromuscular dysfunction. (**B**) Representative M-Mode echocardiographic images of the left ventricle of wild-type (WT) and *Chchd10* male (35 weeks) and female (49 weeks) mice. (**C**) Left ventricular ejection fraction (% LVEF) of WT (blue, *n* = 3–9) and *Chchd10* (red, *n* = 3–8) male mice (left) and WT (blue, *n* = 4–9) and *Chchd10* (red, *n* = 6–7) female mice (right). Data represent mean *± *SEM. The dotted line represents 14 weeks. One-way ANOVA, *P = 0.032548 (6 weeks, male), *P = 0.020173 (13 weeks, male), ****P = 0.000330 (35 weeks, male) and **P = 0.0017454 (37 weeks, female), **P = 0.007404 (49 weeks, female), ns=not significant. (**D**) Representative immunoblots of NPPA protein levels in cardiac lysates of male and female mice at 14 weeks of age. Densitometric quantification of WT (blue, male *n* = 6, female *n* = 4) and *Chchd10* (red, male *n* = 6, female *n* = 4) mice is relative to stain-free. Data are means ± SEM, ordinary one-way ANOVA. ****P < 0.0001. (**E**) Representative immunoblots of CHCHD10 protein accumulation in cardiac lysates of male and female mice at 14 weeks of age. Densitometric quantification of WT (blue, male *n *= 6, female *n* = 7) and *Chchd10* (red, male *n* = 6, female *n* = 6) mice is relative to stain-free. Data are means ± SEM, Ordinary one-way ANOVA. ***P = 0.0006. (**F**) performed on WT and *Chchd10* mutant male (top) and female (bottom) mice at 14 weeks of age. Total (T), insoluble pellet (P), and soluble supernatant (S) fractions were analyzed by immunoblotting with the indicated antibodies. Dotted outline of pellet fraction. (**G**) Venn diagram and heatmap of upregulated differentially expressed genes (DEGs) in *Chchd10* mice. Bulk RNA-seq was performed on cardiac biopsies from male (*n* = 3) and female (*n *= 3) *Chchd10* mice compared to sex-matched littermate controls at 14 weeks of age. The Venn diagram shows 597 overlapping (purple), 157 female-specific (pink), and 356 male-specific (blue) upregulated DEGs. Integrated stress response (ISR) genes (according to Labbé et al, 2024) were significantly upregulated in both sexes (heatmap), and pathway enrichment analysis using Gene Ontology (GO), BioCarta, Reactome, KEGG, and PanglaoDB databases confirmed ISR pathway enrichment among these DEGs. (**H**) Gene Ontology (GO) pathway analyses of female-specific upregulated genes (157) and male-specific upregulated genes (356) identified by bulk RNAseq in (**G**). Source data are available online for this figure.

**Figure EV1:**
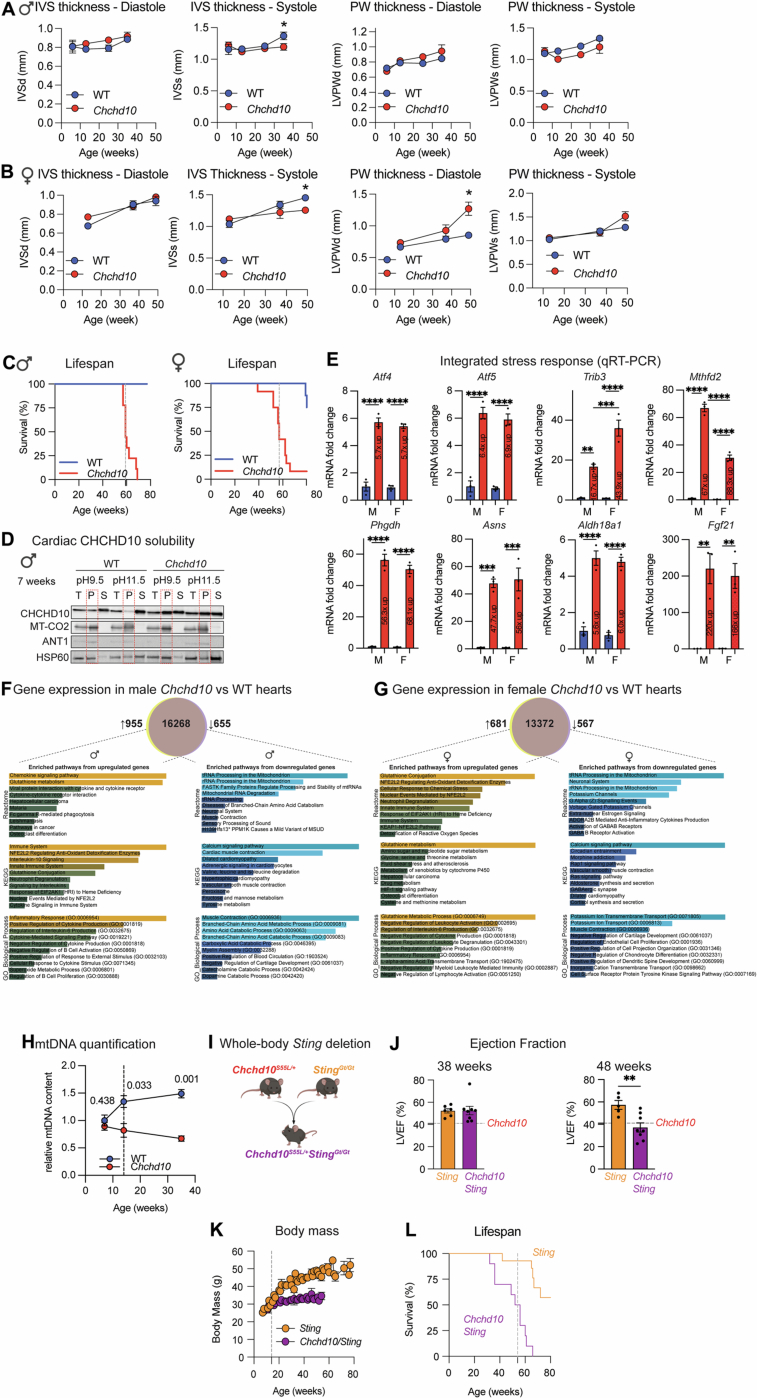
Cardiac remodeling in *Chchd10* mutant hearts. (**A**) Diastolic (IVSd, mm) and Systolic interventricular septum thickness (IVSs, mm) and Diastolic (LVPWd, mm) and Systolic left ventricle posterior wall thickness (LVPWs, mm) of WT (blue, *n* = 3–9) and *Chchd10* (red, *n* = 3–8) male mice in Fig. 1C. Data represent mean *± *SEM. One-way ANOVA, *P = 0.046910. (**B**) Diastolic (IVSd, mm) and Systolic interventricular septum thickness (IVSs, mm) and Diastolic (LVPWd, mm) and Systolic left ventricle posterior wall thickness (LVPWs, mm) of WT (blue, *n* = 4–9) and *Chchd10*^*S55L*^ (red, *n* = 6–7) female mice in Fig. 1C. Data represent mean *± *SEM. One-way ANOVA, IVSs; *P = 0.023457, LVPWd; *P = 0.008310. (**C**) Kaplan–Meier survival curve of WT (blue, *n* = 10) and *Chchd10* (red, *n* = 9) male mice (left) and WT (blue, *n* = 13) and *Chchd10* (red, *n* = 12) female mice (right). Dotted line (gray) represents median lifespan of *Chchd10* mice (male; 62 weeks, Log-rank test, *P* = 0.0006 female 57 weeks, Log-rank test, *P* < 0.0001). (**D**) Alkaline carbonate (Na_2_CO_3_) extraction of cardiac mitochondria performed on WT and *Chchd10* mutant male mice at 7 weeks of age. Total (T), insoluble pellet (P), and soluble supernatant (S) fractions were analyzed by immunoblotting with the indicated antibodies. (**E**) Analysis of integrated stress response (ISR) genes *Atf4*, *Atf5*, *Trib3*, *Mthfd2*, *Phgdh*, *Asns, Aldh18a1*, and *Fgf21* via qRT-PCR of total cardiac biopsies from wild-type (blue, *n* = 3) and *Chchd10* (red, *n* = 3) male and female mice at the indicated ages. Data are relative mean fold changes ± SEM, one-way ANOVA. *Atf4*: WT vs *Chchd10* male ****P < 0.0001, WT vs *Chchd10* female ****P < 0.0001, *Atf5*: WT vs *Chchd10* male ****P < 0.0001, WT vs *Chchd10* female ****P < 0.0001, *Trib3*: WT vs *Chchd10* male **P = 0.0030, WT vs *Chchd10* female ****P < 0.0001, Chchd10 male vs *Chchd10* female ***P = 0.0008, *Mthfd2*; WT vs *Chchd10* male ****P < 0.0001, WT vs *Chchd10* female ****P < 0.0001, Chchd10 male vs *Chchd10* female ****P < 0.0001, *Phgdh*; WT vs *Chchd10* male ****P < 0.0001, WT vs *Chchd10* female ****P < 0.0001, *Asns*; WT vs *Chchd10* male ***P = 0.003, WT vs *Chchd10* female ****P = 0.0002, *Alsh18a1*; WT vs *Chchd10* male ****P < 0.0001, WT vs *Chchd10* female ****P < 0.0001, *Fgf21*; WT vs *Chchd10* male **P = 0.0016, WT vs *Chchd10* female **P < 0.0031. (**F**) Venn diagram of differentially expressed genes (DEGs) in male *Chchd10* versus WT mice. Bulk RNA-seq was performed on cardiac biopsies (*n* = 3) at 14 weeks of age. 955 genes were upregulated in male *Chchd10* vs wild-type (WT) hearts and 655 genes were downregulated. Reactome, KEGG, and Gene Ontology (GO) pathway enrichment performed with Enrichr. (**G**) Venn diagram of differentially expressed genes (DEGs) in female *Chchd10* versus WT mice. Bulk RNA-seq was performed on cardiac biopsies (*n* = 3) at 14 weeks of age. 681 genes were upregulated in female *Chchd10* vs wild-type (WT) hearts and 567 genes were downregulated. Reactome, KEGG, and Gene Ontology (GO) pathway enrichment performed with Enrichr. (**H**) Quantification of mitochondrial DNA (mtDNA) in cardiac biopsies from male WT (blue, *n* = 3), *Chchd10* (red, *n* = 3) mice at 7, 14, and 35 weeks. Primers directed at mtDNA encoding 16 s rRNA and β-actin for nDNA were used. Data are means ± SEM, multiple unpaired *t* test *P* values indicated. (**I**) Whole-body deletion of *Sting* in *Chchd10* mice. Generation of the *Chchd10*^*S55L/+*^*Sting*^*Gt/Gt*^ mice were generated by intercrossing *Chchd10*^*S55L/+*^ (*Chchd10*) with *Sting*^*Gt/Gt*^ mice lacking functional STING (*Sting*). (**J**) Left ventricular ejection fraction (% LVEF) of *Sting* (*Sting*^*Gt/Gt*^, orange, *n* = 6) and *Chchd10/Sting* (*Chchd10*^*S55L/+*^*Sting*^*Gt/Gt*^, purple, *n* = 8) male mice at 38 weeks (left) and 48 weeks (right) of age. Data represent mean *± *SEM. Student’s *t* test, **P < 0.01, ns=not significant. Dotted line represents *Chchd10%*LVEF at 35 weeks. (**K**) Body mass of *Sting* (*Sting*^*Gt/Gt*^, orange, *n* = 3–12), *Chchd10/Sting* (*Chchd10*^*S55L/+*^*Sting*^*Gt/Gt*^, purple= *n* = 3–14) male mice. Data are means ± SEM, 2-tailed unpaired Student’s *t* test used to identify significant differences (*P* < 0.05). Dotted gray line represents the age after which body mass differences between WT and *Chchd10* male (17 weeks) mice. (**L**) Kaplan–Meier survival curve of *Sting* (orange, *n* = 14), *Chchd10/Sting* (purple = *n* = 10) male mice. Dotted line (gray) represents median lifespan of *Chchd10* mice Log-rank test *Chchd10* vs *Chchd10/Sting*, *P* = 0.002. Source data are available online for this figure.

The dominant pathogenic S59L variant in *CHCHD10* responsible for multisystemic mitochondrial dysfunction in humans(Bannwarth et al, 2014) promotes CHCHD10 protein insolubility and compromises cardiac function in heterozygous *Chchd10*^*S55L/+*^ knock-in mice (henceforth *Chchd10* mice), leading to heart failure and death by 1 year (Fig. 1A) (Genin et al, 2019; Anderson et al, 2019). We confirmed these findings in rederived *Chchd10* mutant maintained on a C57Bl6/N background, whose cardiac function we characterized by longitudinal echocardiography (Figs. 1B,C and EV1A,B). While the lifespans of mutant mice were shortened to similar degrees in males and females (Fig. EV1C), we observed differences in cardiac dysfunction at 14 weeks of age: only male mutant mice showed reduced %LVEF (Fig. 1B,C). In line, the cardiac dysfunction biomarker NPPA was upregulated 5.6-fold in males but only fourfold in females *Chchd10* mice (Fig. 1D), suggesting that the onset of cardiac dysfunction is subject to biological sex. On the other hand, CHCHD10 protein accumulation in cardiac lysates were found to be increased to similar levels between male and female *Chchd10* mutant mice (Fig. 1E) and alkaline sodium carbonate (Na_2_CO_3_) extraction studies performed on isolated cardiac mitochondria showed reduced solubility of CHCHD10 in mutant mice of both sexes, both at pH 9.5 and pH 11.5 (Fig. 1F). The increased abundance of CHCHD10 in the insoluble pellet fraction in *Chchd10* mutant cardiac mitochondria did not reflect a general disruption of protein insolubility, as other mitochondrial markers such as MT-CO2, ANT1, and HSP60 behaved similarly between genotypes (Fig. 1F; Appendix Fig. S1). CHCHD10 insolubility preceded the onset of cardiomyopathy in both male (Fig. 1D) and female mice (Fig. 1F), and was associated with an induction of the ISR, which we could measure by cardiac bulk RNAseq (Fig. 1G; Dataset EV1) and qRT-PCR analyses of marker genes *Atf4*, *Atf5*, *Trib3*, *Mthfd2*, *Phdgh*, *Asns, Aldh18a1*, and *Fgf21* (Fig. EV1E). Transcriptomic analyses of differentially expressed genes (DEGs) revealed that induction of core ISR gene expression signatures (Labbé et al, 2024) were similar between male and female *Chchd10* mutant mice when compared to sex-matched wild-type (WT) controls (Figs. 1G and EV1E). Taken together, our data support the model (Sayles et al, 2022) that the earliest defects leading to CHCHD10 insolubility and ISR induction trigger the onset of cardiac dysfunction.

Although transcriptomic analyses revealed the magnitude and onset of ISR induction were similar between male and female mutant hearts (Fig. 1G; Dataset EV2), we observed sex-specific differential gene expression, raising the possibility that cell signaling downstream of mitochondrial dysfunction may modulate the trajectory of cardiomyopathy. The 157 female-specific upregulated DEGs in *Chchd10* mutant hearts were associated with cardioprotective pathways involving BMP10 (Qu et al, 2019) and NR4A3 (Jiang et al, 2019), whose upregulation has been independently demonstrated to protect against cardiomyopathy. In male mutant mice, pathway enrichment based on the 356 upregulated male-specific DEGs revealed an induction of immune, inflammatory, and chemokine signaling characteristic of host-pathogen interaction pathways (including viruses, bacteria, and parasites) (Fig. 1H; Dataset EV2). These enriched pathways also emerged when comparing all 955 upregulated DEGs in symptomatic *Chchd10* male mice to WT littermate male mice, but the same was not observed in females (Fig. EV1F,G, Datasets EV3 and 4). Downregulated pathways including those associated with mtDNA transcription were similarly enriched in both male and female *Chchd10* mice, consistent with a relative reduction of mtDNA we observed at the onset of cardiomyopathy (Genin et al, 2019) (Fig. EV1H). As *Chchd10* mutant mice were housed in specific and opportunistic pathogen-free (SOPF) conditions, we wondered whether overactive innate immune signaling triggered in response to mitochondrial dysfunction may contribute to cardiac inflammation and dysfunction, as in other models of MD (Lei et al, 2021; Oka et al, 2012). To test this hypothesis, we blunted innate immune signaling in *Chchd10* mutant mice via the whole-body ablation of Stimulator of interferon genes (STING) by crossing *Chchd10* mice with the Goldenticket mouse (*Sting*^*Gt/Gt*^, Fig. EV1I), which carries a loss-of-function I199N mutation in *Sting*, effectively knocking out the gene (Sauer et al, 2011). *Sting* encodes a transmembrane ER protein that acts as an adapter protein involved in interferon signaling that can be triggered by microbial infection and mitochondrial dysfunction to activate innate immunity (Lei et al, 2023). Echo analyses of *Chchd10*^*S55L/+*^*Sting*^*Gt/Gt*^ (henceforth C*hchd10/Sting*) mutant mice revealed rescued cardiac function at 38 weeks but impaired %LVEF at 48 weeks of age (Fig. EV1J), pointing to a temporary, cardioprotective effect of inhibiting overactive innate immune signaling downstream of mitochondrial dysfunction. *Sting* deletion did not negatively impact cardiac function in *Sting*^*Gt/Gt*^ (henceforth *Sting*) mice (Fig. EV1J) and did not influence the reduced body mass or lifespan shortening in *Chchd10/Sting* mice (Fig. EV1K,L). Taken together, our data indicate that inflammatory signaling triggered by mitochondrial dysfunction can modulate the progression of cardiac dysfunction downstream caused by mutant CHCHD10 insolubility.

### Impaired mitochondrial respiration is an early defect in mutant *Chchd10* hearts

**Figure 2:**
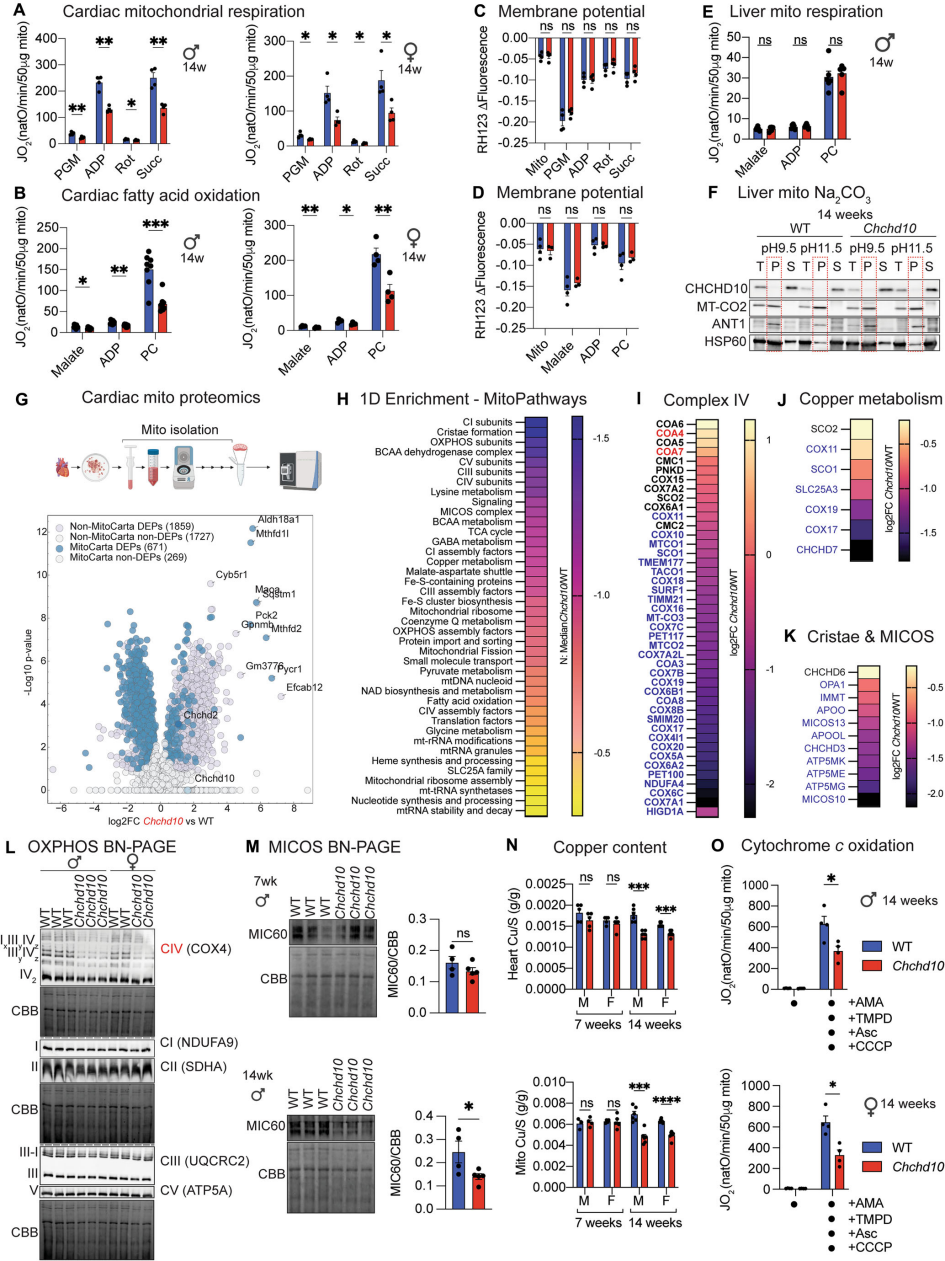
Impaired mitochondrial respiration in mutant *Chchd10* hearts. (**A**) (Left) Oxygen consumption rates (JO_2_) of cardiac mitochondria isolated from WT (*n* = 4) and *Chchd10* (*n* = 4) male mice at 14 weeks. Data represent mean ± SEM; multiple unpaired *t* test. JO_2_ measured sequentially in the presence of pyruvate, glutamate, malate (PGM, **P = 0.004660), adenosine diphosphate (ADP, **P = 0.000351), rotenone (Rot, *P = 0.031108), and succinate (Succ, **P = 0.001588). (Right) Oxygen consumption rates (JO_2_) of cardiac mitochondria isolated from WT (*n* = 4) and *Chchd10* (*n* = 4) female mice at 14 weeks. Data represent mean ± SEM; multiple unpaired *t* test. JO_2_ measured sequentially in the presence of pyruvate, glutamate, malate (PGM, *P = 0.019533), adenosine diphosphate (ADP, *P = 0.010106), rotenone (Rot, *P = 0.021817), and succinate (Succ, *P = 0.024415). (**B**) (Left) Oxygen consumption rates (JO_2_) of cardiac mitochondria isolated from WT (*n* = 4–8) and *Chchd10* (*n* = 8) male mice at 14 weeks. Data represent mean ± SEM; multiple unpaired *t* test. JO_2_ measured sequentially in the presence of malate (*P = 0.018794), adenosine diphosphate (ADP, **P = 0.001275), and palmitoyl carnitine (PC, ***P = 0.000299). (Right) Oxygen consumption rates (JO_2_) of cardiac mitochondria isolated from WT (*n* = 4–8) and *Chchd10* (*n* = 4) female mice at 14 weeks. JO_2_ measured sequentially in the presence of malate (**P = 0.004820), adenosine diphosphate (ADP, *P = 0.031068), and palmitoyl carnitine (PC, **P = 0.006237). (**C**) Mitochondrial membrane potential (ΔΨ) measured by quenching of Rhodamine 123 (RH123) fluorescence in cardiac mitochondria of WT (*n* = 4) and *Chchd10* (*n* = 4) female mice from (**A**, right). Data represent mean ± SEM; multiple unpaired *t* test, ns=not significant. (**D**) Mitochondrial membrane potential (ΔΨ) measured by quenching of Rhodamine 123 (RH123) fluorescence in cardiac mitochondria of WT (*n* = 4) and *Chchd10* (*n* = 3) male mice from (**B**, right). Data represent mean ± SEM; multiple unpaired *t* test, ns=not significant. (**E**) Oxygen consumption rates (JO_2_) of liver mitochondria isolated from WT (*n* = 6) and *Chchd10* (*n* = 6) male mice at 14 weeks. JO_2_ measured sequentially in the presence of malate, adenosine diphosphate (ADP), and palmitoyl carnitine (PC). Data represent mean ± SEM; multiple unpaired *t* test, ns=not significant. (**F**) Alkaline carbonate (Na_2_CO_3_) extraction of liver mitochondria performed on WT and *Chchd10* mutant male mice at 14 weeks of age. Total (T), insoluble pellet (P), and soluble supernatant (S) fractions generated at pH 9.5 and 11.5 were analyzed by immunoblotting with the indicated antibodies. Dotted outline of pellet fraction. (**G**) Volcano plot of differentially expressed proteins (DEPs) identified by proteomics of isolated cardiac mitochondria (Dataset EV5) from WT (*n* = 4) and *Chchd10* (red, *n* = 4) male mice at 14 weeks of age. In total, 671 DEPs belonging to MitoCarta 3.0 were identified. Horizontal dotted line represents −log_10_(padj) >0.05. Two-sided unpaired *t* test followed by permutation-based FDR correction. (**H**) 1D enrichment analysis of MitoPathways (MitoCarta 3.0), revealing the dysregulated pathways in (**G**) (Dataset EV5). (**I**) Heatmap of Complex IV proteins and assembly factors that were quantified by cardiac proteomics in (**G**) and significantly upregulated (red), downregulated (blue), or unchanged (black) in *Chchd10* cardiac mitochondria relative to wild-type. (**J**) Heatmap of Copper metabolism proteins that were quantified by proteomics in (**G**) and significantly downregulated (blue) or unchanged (black) in *Chchd10* cardiac mitochondria relative to wild-type (Dataset EV5). (**K**) Heatmap of Cristae and MICOS complex proteins that were quantified by proteomics in (**G**) and significantly downregulated (blue) or unchanged (black) in *Chchd10* cardiac mitochondria relative to wild-type (Dataset EV5). (**L**) BN-PAGE immunoblot analysis of cardiac OXPHOS complexes isolated from WT and *Chchd10* male and female mice at 14 weeks using the indicated antibodies for Complex IV (COX4), Complex I (NDUFA9), Complex II (SDHA), Complex III (UQCRC2), and Complex V (ATP5A). Coomassie brilliant blue (CBB) was used as a loading control. Quantification in Fig. EV2F. (**M**) Representative BN-PAGE immunoblot analysis of cardiac MICOS complexes isolated from WT (*n* = 3) and *Chchd10* (*n* = 3) male mice at 7 and 14 weeks using the indicated antibody against MIC60. Densitometric quantification is relative to Coomassie brilliant blue (CBB). Data are means ± SEM, two-tailed unpaired Student’s *t* test. *P = 0.0440, ns=not significant. (**N**) (Top) Copper content in total heart (top, *n* = 5–6) samples measured by inductively coupled plasma–optical emission spectrometry (ICP-OES) from wild-type (WT) and *Chchd10* male (M) and female (F) mice at 7 and 14 weeks of age. Data represent mean values normalized to sulfur (S) ± SEM, multiple two-tailed unpaired Student’s *t* test. 14 weeks M; WT vs Chchd10, ***P < 0.000110, 14 weeks F; WT vs Chchd10, ***P < 0.000170, ns=not significant. (Bottom) Copper content in cardiac mitochondria (bottom, *n* = 3–5) samples measured by inductively coupled plasma–optical emission spectrometry (ICP-OES) from wild-type (WT) and *Chchd10* male (M) and female (F) mice at 7 and 14 weeks of age. 14 weeks M; WT vs *Chchd10*, ***P < 0.000189, 14 weeks F; WT vs *Chchd10*, ****P < 0.000015, ns=not significant. (**O**) Oxygen consumption rates (JO_2_) of cardiac mitochondria from WT (*n* = 4) and *Chchd10* (*n* = 4) male (top) and female (bottom) mice at 14 weeks incubated with antimycin A (AMA) to prevent electron transfer from Complex III followed by addition of carbonyl cyanide m-chlorophenyl hydrazine (CCCP), and N,N,N′,N′-Tetramethyl-p-phenylenediamine (TMPD) and ascorbate (Asc) to measure cytochrome *c* oxidation. Data represent mean ± SEM; unpaired Student’s *t* test, male; *P = 0.024444, female; *P = 0.007250. Source data are available online for this figure.

**Figure EV2:**
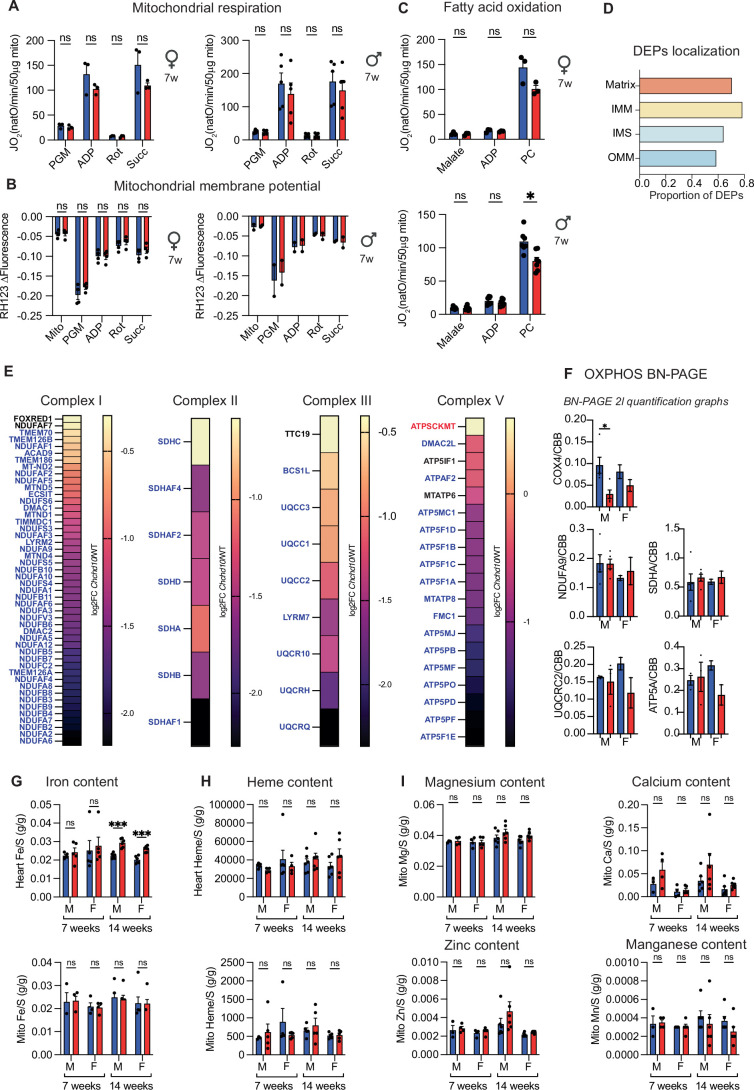
Impaired mitochondrial respiration in mutant *Chchd10* hearts. (**A**) Oxygen consumption rates (JO_2_) of cardiac mitochondria isolated from WT (*n* = 3–5) and *Chchd10* (*n* = 3–5) female (left) and male (right) mice at 7 weeks. JO_2_ measured sequentially in the presence of pyruvate, glutamate, malate (PGM), adenosine diphosphate (ADP), rotenone (Rot), and succinate (Succ). Data represent mean ± SEM; multiple unpaired *t* test, ns=not significant. (**B**) Mitochondrial membrane potential (ΔΨ) measured by quenching of Rhodamine 123 (RH123) fluorescence in cardiac mitochondria of WT and *Chchd10* male (*n* = 5) and female (*n* = 2) mice from Fig. EV2A. Data represent mean ± SEM; multiple unpaired *t* test, ns=not significant. (**C**) Oxygen consumption rates (JO_2_) of cardiac mitochondria isolated from WT (*n* = 3–7) and *Chchd10* (*n* = 3–7) female (top) and male (bottom) mice at 7 weeks. JO_2_ measured sequentially in the presence of malate, adenosine diphosphate (ADP), and palmitoyl carnitine (PC). Data represent mean ± SEM; multiple unpaired *t* test, *P = 0.003882, ns=not significant. (**D**) Mitochondrial localization of DEPs in Fig. 2G according to MitoCarta 3.0 represented as proportion of total quantified DEPs in each mitochondrion subcompartment. (**E**) Heatmap of Complex I, II, III, and V proteins quantified by proteomics in Fig. 2G and significantly upregulated (red), downregulated (blue) or unchanged (black) between WT (*n* = 5) and *Chchd10* (*n* = 5) male mice at 14 weeks (Dataset EV5). (**F**) Densitometric quantification of OXPHOS complexes in Fig. 2L is relative to Coomassie brilliant blue (CBB). Data are means ± SEM, one-way ANOVA. *P = 0.0252. (**G**) Iron content in total heart (top, *n* = 5–6) and cardiac mitochondria (bottom, *n* = 3–6) samples measured by inductively coupled plasma–optical emission spectrometry (ICP-OES) from WT and *Chchd10* male (M) and female (F) mice at 7 and 14 weeks of age. Data represent mean values normalized to sulfur (S) ± SEM, multiple unpaired *t* test, 14 weeks M; WT vs *Chchd10*, ***P < 0.000112, 14 weeks F; WT vs *Chchd10*, ***P < 0.000233, ns=not significant. (**H**) Heme content in total heart (top, *n* = 5–6) and cardiac mitochondria (bottom, *n* = 3–6) samples measured by HPLC from WT and *Chchd10* male (M) and female (F) mice at 7 and 14 weeks of age. Data represent mean values normalized to sulfur (S) ± SEM, multiple unpaired *t* test, ns=not significant. (**I**) Magnesium, Zinc, Calcium, and Manganese content in cardiac mitochondria measured by inductively coupled plasma–optical emission spectrometry (ICP-OES) from WT and *Chchd10* male (M) and female (F) mice at 7 and 14 weeks of age. Data represent mean values normalized to sulfur (S) ± SEM, multiple unpaired *t* test, ns=not significant. Source data are available online for this figure.

Mutant CHCHD10 triggers ISR induction and subsequently the metabolic rewiring of iron-dependent and iron-sulfur cluster (ISC) pathways that are required for OXPHOS function (Sayles et al, 2022). Since OXPHOS dysfunction was observed in late-stage *Chchd10* mice, this led to the notion that impaired mitochondrial respiration is a consequence rather than a cause of cardiomyopathy (Sayles et al, 2022). When we measured oxygen consumption rates by high-resolution respirometry in cardiac mitochondria isolated from *Chchd10* mutant mice beginning at 14 weeks of age, we observed oxygen consumption defects on both carbohydrate and fatty acid-derived substrates (Fig. 2A,B), which were equivalently reduced in both (symptomatic) male and (presymptomatic) female mutant mice. These defects were not accompanied by loss of mitochondrial membrane potential, arguing against mitochondrial uncoupling (Fig. 2C,D). We observed normal mitochondrial respiration from livers of *Chchd10* mice, in which CHCHD10 solubility is unaffected (Fig. 2E,F), further strengthening the association between CHCHD10 insolubility and bioenergetic dysfunction. Mitochondrial respiration and membrane potential were unaffected in *Chchd10* cardiac mitochondria isolated from presymptomatic male mice at 7 weeks of age (Fig. EV2A–C). These data argue that bioenergetic impairment of mitochondria is an early dysfunction associated with CHCHD10 insolubility, which can precede or manifest coincidently with cardiac dysfunction.

To understand why mitochondrial respiration was reduced in response to mutant CHCHD10, we compared the proteomes of cardiac mitochondria isolated from WT and *Chchd10* mice, which revealed a dramatic remodeling: 71% of the 940 quantified mitochondrial proteins belonging to MitoCarta 3.0 were differentially expressed, with most of them being downregulated (average log2 FC = -0.7) (Fig. 2G). Stratifying the MitoCarta 3.0 DEPs based on known submitochondrial localization (OMM, IMS, IMM, or matrix) did not reveal submitochondrial compartment biases, highlighting a generally uniform dysregulation of mitochondrial proteostasis (Fig. EV2D). One-dimensional enrichment analyses revealed several MitoPathways, notably those implicated in the maintenance and assembly of OXPHOS and MICOS complexes and copper metabolism (Figs. 2H–K and EV2E). Plotting quantified MitoCarta 3.0 proteins revealed significant reductions in the steady-state levels of components of Complex IV (Fig. 2I) as well as other OXPHOS complexes (Fig. EV2E). BN-PAGE analyses revealed normal levels of Complexes I, II, III, and V and a specific reduction in Complex IV assemblies (Figs. 2L and EV2F), which accompanied the reduced levels of subunits and assembly factors such as COA8, COX16, COX18, COX20, COX4L1, COX5A, COX6B1, COX6C, COX7A1, COX7A2L, COX7B, COX7C, COX8B, COX10, COX15, COX18, COX20, and the mtDNA-encoded proteins MT-CO1, MT-CO2 and MT-CO3. We observed a reduction in HIGD1A (Fig. 2I), which coordinates the assembly of COX-containing complexes (Timón-Gómez et al, 2020), and a shift in the balance between COX6A1 and COX6A2 isoforms, which were reported to impact the stability of COX-containing supercomplexes (Cogliati et al, 2016). High-resolution fluor-respirometry performed in isolated cardiac mitochondria in the presence of Antimycin A, TMPD, Ascorbate, and CCCP, which is typically used to measure Complex IV activity (Villani and Attardi, 1997), revealed a ~40% decrease in oxygen consumption rates (JO_2_) in both (symptomatic) male and (presymptomatic) female mice at 14 weeks (Fig. 2O), pointing to a defect in cytochrome *c* oxidation. We observed reductions in copper handling proteins COX11, COX19, SCO1, CHCHD7, and the IMM copper transporter SLC25A3 (Fig. 2J), all of which are required for the metalation of COX (Cobine et al, 2021). In addition, COX17, which supplies the Cu necessary via SCO1 to assemble both the COX1 and COX2 modules required to assemble the COX holoenzyme, was also reduced. In yeast, COX17 is involved in the assembly of COX as well as the MICOS complex (Chojnacka et al, 2015), an integral membrane complex that bridges outer and inner membranes and is required to maintain cristae structure (Anand et al, 2021). Proteomic profiling of isolated cardiac mitochondria also showed a reduction in MICOS subunits MIC60/IMMT, MIC13, CHCHD3, APOO, and APOOL and the MICOS-interactor OPA1 (Fig. 2K), which also plays a central role in the maintenance of cristae structure (Frezza et al, 2006). In line, BN-PAGE analysis of the MICOS complex revealed a reduction in MIC60 immunoreactivity at 14 weeks (but not 7 weeks) of age (Fig. 2M), which paralleled the disruption previously reported in *CHCHD10*^*S59L/+*^ patient-derived fibroblasts (Genin et al, 2022).

Copper is an essential redox cofactor for several mitochondrial enzymes, including Cytochrome *c* oxidase (Complex IV), whose metalation in the CuA and CuB sites is essential for assembly and enzymatic activity (Cobine et al, 2021). As genetic defects in disrupting the insertion of copper into Complex IV cause severe, multisystemic defects including heart failure in mice and humans (Papadopoulou et al, 1999; Stroud et al, 2015; Valnot et al, 2000b; Leary et al, 2007; Baker et al, 2017; Boulet et al, 2018), we measured copper levels in *Chchd10* mutant hearts by inductively coupled plasma optical emission spectroscopy (ICP-OES), which enables the direct, precise, sensitive, and accurate measurement of metals in biology (Cubadda, 2007). ICP-OES analyses uncovered a reduction in cardiac and mitochondrial (Fig. 2N) copper content in 14-week-old mutant male and female mice, which paralleled the observed reduction in cytochrome *c* oxidation rates (Fig. 2O). In contrast to previous studies (Sayles et al, 2022), no reductions in iron or heme were observed at either 7 or 14 weeks of age (Fig. EV2G,H). We observed an ~1.3-fold increase in total cardiac (but not mito) iron levels in male (1.31-fold) and female (1.29-fold) *Chchd10* mutant mice at 14 weeks. ICP-OES revealed other metals such as zinc (Zn), magnesium (Mg), or manganese (Mn) were unaltered in *Chchd10* hearts and cardiac mitochondria (Fig. EV2I). Altogether, our data reveal that a defect in CHCHD10 protein solubility is associated with impaired cytochrome *c* oxidation, which may contribute to the pathological cardiac remodeling in *Chchd10* mutant mice.

### Inhibition of OMA1 catalytic activity suppresses mtISR but not CHCHD10 insolubility

**Figure 3:**
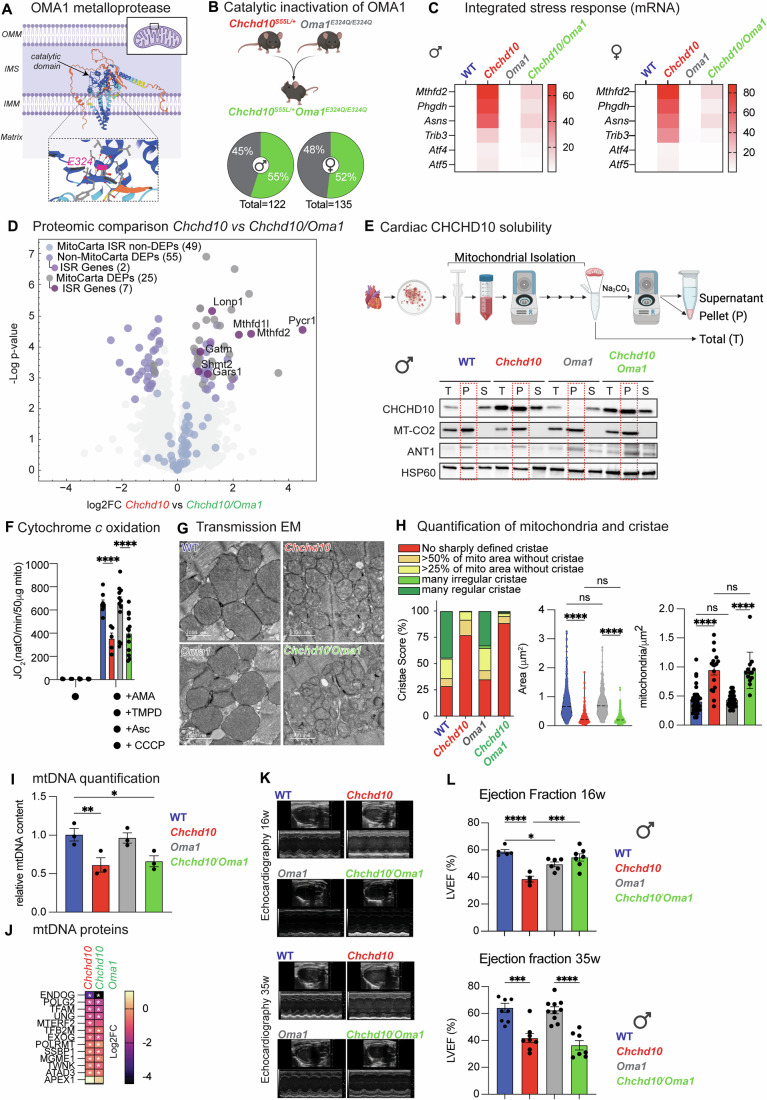
*Oma1*^*E324Q/E324Q*^ mice suppress mtISR and bypass mitochondrial dysfunction. (**A**) Structural representation of OMA1 metalloprotease with Alphafold 3.0. Catalytic core including the E324 glutamic acid residue within the conserved HEXXH motif that is essential for zinc binding and catalytic activity is indicated in the inset. Substitution to glutamine (E324Q) inhibits catalytic activity. (**B**) Generation of the *Chchd10*^*S55L/+*^*Oma1*^*E324Q/E324Q*^ (*Chchd10/Oma1*, green) mice by intercrossing *Chchd10*^*S55L/+*^ (*Chchd10*, red) with *Oma1*^*E324Q/E324Q*^ (*Oma1*, gray) mice. Generation of male (*n* = 122) and female (*n* = 135) offspring from *Chchd10/Oma1* intercrosses with *Oma1* mice according to Mendelian ratios: chi-squared for male 1.18 < 3.84 (*P* = 0.05, df = 1) and female 0.185 < 3.84 (*P* = 0.05, df = 1) mice. (**C**) Heatmap of integrated stress response (ISR) genes expression measured by qRT-PCR of total cardiac biopsies from wild-type (blue, *n* = 3), *Chchd10* (red, *n* = 3), *Oma1* (gray, *n* = 3), and *Chchd10/Oma1* (green, *n* = 3) male and female mice at 14 weeks (see Fig. EV3F,G for one-way ANOVA). (**D**) Volcano plot of cardiac proteomics performed on cardiac mitochondria isolated from male WT (*n* = 5) and *Chchd10* (*n* = 5) mutant male mice at 14 weeks of age. Proteins ascribed to MitoCarta 3.0 and integrated stress response (ISR) genes are indicated. Differentially expressed proteins (DEPs) plotted according to the log2 fold change of *Chchd10* vs *Chchd10/Oma1* versus the -log10 transformed *P* value of a two-sided *t* test. Significance was considered for a permutation-based FDR cutoff of 0.05. Mitochondrial (MitoCarta 3.0) and ISR Genes as previously defined (Labbé et al, 2024) are highlighted by color. (**E**) Alkaline carbonate (Na_2_CO_3_) extraction of cardiac mitochondria performed on cardiac mitochondria isolated from wild-type (WT), *Chchd10*, *Oma1*, and *Chchd10/Oma1* mice at 14 weeks of age. Total (T), insoluble pellet (P), and soluble supernatant (S) fractions were analyzed by immunoblotting with the indicated antibodies. Dotted outline of pellet fraction. (**F**) Oxygen consumption rates (JO_2_) of cardiac mitochondria from male WT (blue, *n* = 8), *Chchd10* (red, *n* = 5), *Oma1* (gray, *n* = 13), and *Chchd10/Oma1* (green, *n* = 13) male mice at 14 weeks incubated with antimycin A (AMA) to prevent electron transfer form Complex III followed by addition of carbonyl cyanide m-chlorophenyl hydrazine (CCCP), and N,N,N′,N′-Tetramethyl-p-phenylenediamine (TMPD) and ascorbate (Asc) to measure cytochrome *c* oxidation. Data represent mean ± SEM; unpaired Student’s *t* test. Two-way ANOVA, WT vs *Chchd10*; ****P < 0.0001, *Oma1* vs *Chchd10/Oma1*; ****P < 0.0001. (**G**) Representative transmission electron micrographs (EM) of cardiac posterior walls of WT (blue, *n* = 3) and *Chchd10* (red, *n* = 3) *Oma1* (gray, *n* = 3), and *Chchd10/Oma1* (green, *n* = 3) male mice at 14 weeks. Scale bar: 1000 nm. (**H**) Quantification of (**G**) performed with ImageJ to assess the area of manually segmented mitochondria area (mm^2^) and the number of mitochondria per image area (mitochondria/mm^2^) from WT (blue, *n* = 3), *Chchd10* (red, *n* = 3), *Oma1* (gray, *n* = 3), and *Chchd10/Oma1* (green, *n* = 3) mice at 14 weeks. In total, 315–440 mitochondria were quantified per condition. The Cristae score was assigned blindly according to a previously defined classification (Eisner et al, 2017). Data are means ± SEM, one-way ANOVA. Area (μm^2^): WT vs *Chchd10*; ****P < 0.0001, *Oma1* vs *Chchd10/Oma1*; ****P < 0.0001. Mitochondria/μm^2^: WT vs *Chchd10*; ****P < 0.0001, *Oma1* vs *Chchd10/Oma1*; ****P < 0.0001. Thick dotted lines represent median values. (**I**) Quantification of mitochondrial DNA (mtDNA) in cardiac biopsies from male WT (blue, *n* = 3), *Chchd10* (red, *n* = 3), *Oma1* (gray, *n* = 3), and *Chchd10/Oma1* (green, *n* = 3) mice at 14 weeks. Primers directed at mtDNA encoding 16 s rRNA and β-actin for nDNA were used. Data are means ± SEM, one-way ANOVA, *P = 0.0145, **P = 0.0076. (**J**) Heatmap of significantly downregulated (*) mtDNA maintenance proteins in cardiac mitochondria profiled from *Chchd10* (red, *n* = 5) versus WT (*n* = 5) and *Chchd10/Oma1* (green, *n* = 5) vs WT hearts by mass spectrometry (Dataset EV5). (**K**) Representative M-Mode echocardiographic images of the left ventricle of wild-type (WT), *Chchd10*, *Oma1*, and *Chchd10/Oma1* male mice at 16 weeks (top) and 35–37 weeks (bottom) of age. Wild-type (WT) and *Chchd10* images (bottom) are reused from Fig. 1B. (**L**) Left ventricular ejection fraction (% LVEF) of WT (blue, *n *= 5–8), *Chchd10* (red, *n* = 4–8), *Oma1* (gray, *n* = 6–10), and *Chchd10/Oma1* (green, *n* = 7) male mice at 16 weeks (top) and 35 weeks (bottom) of age. WT and *Chchd10* data at 35 weeks are those graphed in Fig. 1C. Data represent mean *± *SEM. One-way ANOVA, 16 weeks: WT vs *Chchd10*; ****P < 0.0001, WT vs *Oma1*; *P < 0.0383, *Chchd10* vs *Chchd10/Oma1* ***P = 0.0007. 35 weeks: WT vs *Chchd10*; ****P = 0.0001, *Chchd10* vs *Chchd10/Oma1* ****P < 0.0001. Source data are available online for this figure.

**Figure EV3:**
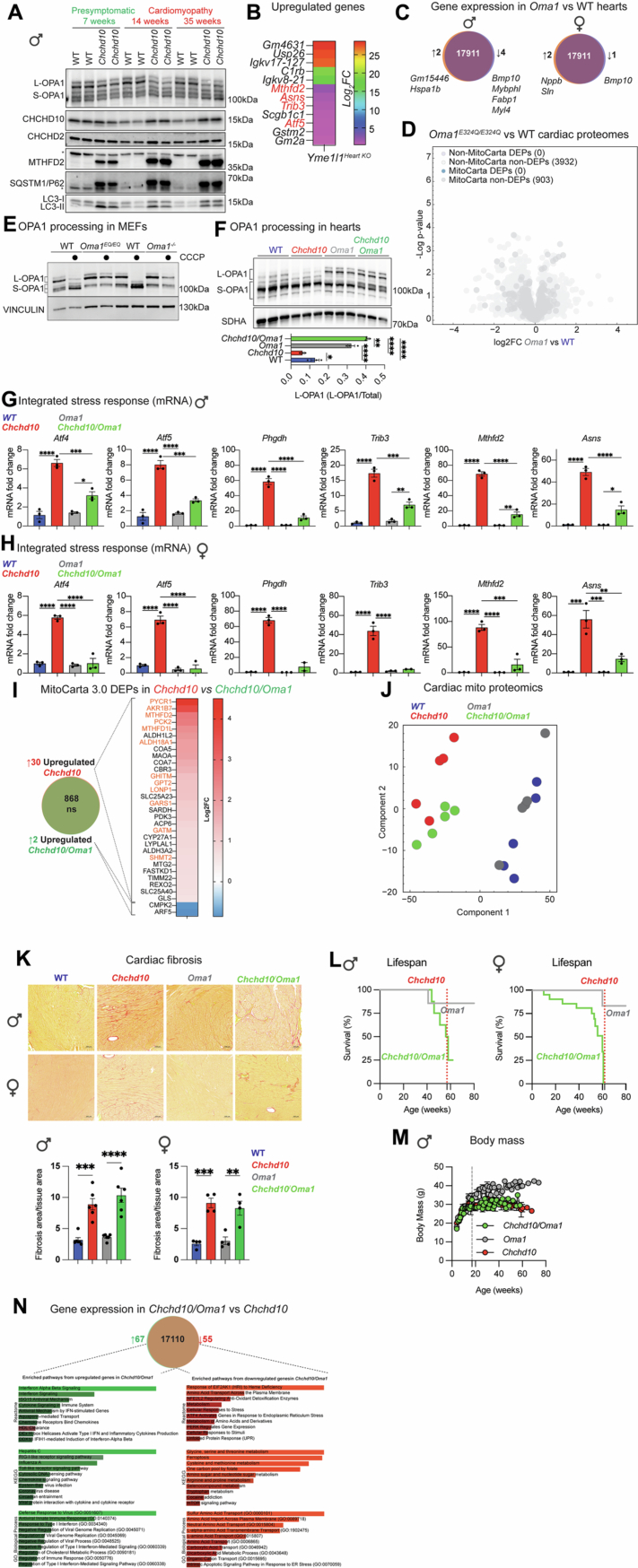
OMA1 and mtISR activation. (**A**) Immunoblots in cardiac lysates of presymptomatic (green) and symptomatic (red) WT and *Chchd10* mice. (**B**) Heatmap of upregulated differentially expressed genes (DEGs) in cardiomyocyte-specific *Yme1l* knockout mice (*Yme1l1*^*HeartKO*^). Bulk RNA-seq was performed on cardiac biopsies from male (*n* = 3) mice compared to sex-matched littermate controls at 35 weeks of age. Integrated stress response (ISR) genes are highlighted red. (**C**) Venn diagram of differentially expressed genes (DEGs) in *Oma1* mice. Bulk RNA-seq was performed on cardiac biopsies from male (*n* = 3) and female (*n* = 3) *Oma1* mice compared to sex-matched WT controls at 14 weeks of age. DEG is defined by Log2FC > 2 and padj <0.01 (Dataset EV1). (**D**) Volcano plot of cardiac proteomics WT vs Oma1 No differentially expressed proteins (DEPs) were identified. Two-sided unpaired *t* test followed by permutation-based FDR correction. Significance is considered a permutation-based FDR at 0.05 highlighted by color. (**E**) Immunoblots of OPA1 processing in mouse embryonic fibroblasts (MEFs) derived from wild-type (WT) and *Oma1*^*E324Q/E324Q*^ (*Oma1*^*EQ/EQ*^) mice. Carbonyl cyanide m-chlorophenyl hydrazine (CCCP) used to induce stress-induced L-OPA1 processing. WT and OMA1 knockout (*Oma1*^*-/-*^) MEFs were used as a control (Wai et al, 2015). (**F**) (Top) Immunoblot of L-OPA1 and S-OPA1 in cardiac mitochondria of WT (blue, *n* = 3), *Chchd10* (red, *n* = 3), *Oma1* (gray, *n* = 3), and *Chchd10/Oma1* (green, *n* = 3) mice between 17 and 19 weeks. SDHA used as loading control(Bottom) Densitometric quantification of L-OPA1/Total OPA1. Data are mean ± SEM, One-way ANOVA, *P < 0.05, **P < 0.01, ****P < 0.0001. (**G**) Analysis of integrated stress response (ISR) genes via qRT-PCR of total cardiac biopsies from wild-type (WT, blue, *n* = 3), *Chchd10* (red, *n* = 3), *Oma1* (gray, *n* = 3), and *Chchd10/Oma1* (green, *n* = 3) male mice at 14 weeks in Fig. 3C. Data are relative mean fold changes ± SEM, one-way ANOVA, *Atf4*; *P = 0.259, ***P < 0.0005,****P < 0.0001, *Atf5*; ***P = 0.0001,****P < 0.0001, *Phgdh*; ****P < 0.0001, *Trib3*; **P = 0.0098, ***P = 0.0001, ****P < 0.0001, *Mthfd2*; **P = 0.0071, ****P < 0.0001, *Asns*; *P = 0.0188, ****P < 0.0001. (**H**) Analysis of integrated stress response (ISR) genes via qRT-PCR of total cardiac biopsies from wild-type (WT, blue, *n* = 3), *Chchd10* (red, *n* = 3), *Oma1* (gray, *n* = 3), and *Chchd10/Oma1* (green, *n* = 2–3) female mice at 14 weeks in Fig. 3C. Data are relative mean fold changes ± SEM, one-way ANOVA, *Atf4*; ****P < 0.0001, *Atf5*; ****P < 0.0001, *Phgdh*; ****P < 0.0001, *Trib3*; ****P < 0.0001, *Mthfd2*; ***P = 0.0001, ****P < 0.0001, *Asns*; **P = 0.0018, ***P = 0.0002. (**I**) Heatmap of differentially expressed proteins (DEPs) upregulated in cardiac mitochondria profiled from *Chchd10* (red, *n* = 5) versus *Chchd10/Oma1* (green, *n* = 5) hearts by mass spectrometry (Dataset EV5). (**J**) Principal component analysis (PCA) of cardiac proteomics performed on wild-type (WT, *n* = 5, blue), *Chchd10* (*n* = 5, red), *Oma1* (*n *= 5, gray) and *Chchd10/Oma1* (*n* = 5, green) male mice at 14 weeks of age (Dataset EV5). (**K**) Cardiac histology of 4 genotypes and both sexes. Representative Sirius red myocardium staining of wild-type (WT), *Chchd10*, *Oma1*, and *Chchd10/Oma1* male (22 weeks of age, *n* = 6) and female (22 weeks of age, *n* = 4) mice. Tissue fibrosis analysis was performed using an automated macro developed in QuPath and quantified (bottom). Data represent mean *± *SEM. One-way ANOVA, Male; WT vs *Chchd10* ***P = 0.0002, *Oma1* vs *Chchd10/Oma1* ****P < 0.0001, Female; WT vs *Chchd10* ***P = 0.0003, *Oma1* vs *Chchd10/Oma1* **P < 0.0019, ns=not significant. (**L**) Kaplan–Meier survival curve (left) of *Oma1* (gray, *n* = 15), and *Chchd10/Oma1* (green, *n* = 15) male mice and (right) *Oma1* (gray, *n* = 13), and *Chchd10/Oma1* (green, *n* = 21) female mice. Dotted red line represents median lifespan of *Chchd10* mice. Log-rank test *Chchd10* vs *Chchd10/Oma1*; male *P* = 0.0844 and female *P* = 0.2520. (**M**) Body mass of *Oma1* (gray), and *Chchd10/Oma1* (green) male mice compared to data from male *Chchd10* mice. Dotted gray line represents the age after which body mass differences between WT and *Chchd10* male (17 weeks) mice are observed. (**N**) Venn diagram of differentially expressed genes (DEGs) in female *Chchd10/Oma1* versus *Chchd10* mice. Bulk RNA-seq was performed on cardiac biopsies (*n* = 3) at 14 weeks of age. Reactome, KEGG, and Gene Ontology (GO) pathway enrichment performed with Enrichr. Source data are available online for this figure.

Previous studies of *Chchd10* mice have intimated that the mtISR induction is responsible for OXPHOS dysfunction via the maladaptive rewiring of mitochondrial metabolism (Sayles et al, 2022; Southwell et al, 2024; Anderson et al, 2019). CHCHD10^S59L^ triggers the activation of the stress-induced metalloprotease OMA1, which proteolytically processes DELE1 in the IMS so that the cleaved form can be exported to the cytosol where it signals through the heme-regulated inhibitor (HRI) kinase to activate the ISR (Shammas et al, 2022; Guo et al, 2020; Fessler et al, 2020; Sekine et al, 2023; Fessler et al, 2022). Monitoring the proteolytic cleavage of another classical OMA1 substrate L-OPA1, revealed OMA1 activation in symptomatic *Chchd10* mutant mice (14 weeks of age) that was limited in presymptomatic hearts (7 weeks of age), which showed elevated levels of MTHFD2, SQSTM1/P62, and LC3-II (Fig. EV3A), indicating that stress-induced OPA1 processing occurs after ISR induction. Since OMA1 ablation can suppress cardiac dysfunction and mitochondrial fragmentation in the hearts of cardiomyocyte-specific *Yme1l1* knockout mice (*Yme1l1*^*Heart*^), which also show increased OMA1 activity (Wai et al, 2015) and ISR signaling according to bulk RNAseq studies performed at 35 weeks of age (Fig. EV3B), we wondered whether inactivation of OMA1 could confer cardioprotection to *Chchd10* mutant mice. Whole-body deletion of *Oma1* is synthetically lethal in *Chchd10* mutant mice carrying the G58R variant (Shammas et al, 2022), prompting us to generate a catalytic site mutant E324Q in OMA1 via Crispr/Cas9 genome editing of C57Bl/6 N mice (Fig. 3A,B), which is known to inhibit the proteolytic activity of OMA1 without disrupting putative, non-catalytic scaffolding functions in the IMM (Wai et al, 2016; Baker et al, 2014). *Oma1*^*E324Q/E324Q*^ (henceforth *Oma1*) mutant mice were outwardly normal, and cardiac transcriptomic profiling performed at 14 weeks of age revealed virtually no gene dysregulation: 6 and 3 out of 17,1911 genes were differentially expressed in *Oma1* male and female mice, respectively (Fig. EV3C; Dataset EV1). Similarly, proteomic comparisons of isolated cardiac mitochondria from WT and *Oma1* male mice revealed no differentially expressed proteins (DEPs) (Fig. EV3D; Dataset EV5), consistent with previous proteomics analyses of cardiomyocyte-specific *Oma1* knockout mice (Ahola et al, 2022). We confirmed the catalytic inactivation of OMA1 in mouse fibroblasts derived from *Oma1* embryos by examining constitutive and CCCP-induced OPA1 processing (Fig. EV3E) and cardiac mitochondria (Fig. EV3F). Next, we intercrossed *Oma1* and *Chchd10* mutant mice and successfully generated *Oma1*^*E324Q/E324Q*^*Chchd10*^*S55L/+*^ (*Chchd10/Oma1*) double mutant mice at Mendelian ratios (Fig. 3B). Double mutant male and female mice, which were outwardly normal, showed a marked reduction in cardiac ISR induction by RT-qPCR, and bulk RNAseq analyses (Figs. 3C and EV3G,H; Dataset EV1,6) and OMA1-dependent L-OPA1 processing (Fig. EV3F), further validating the suppression of OMA1 activity and the mtISR in these mice. Similarly, proteomic profiling in male mice revealed a reduction in ISR protein levels in *Chchd10/Oma1* isolated cardiac mitochondria in comparison to those isolated from *Chchd10* mice (Fig. 3D). Most of the upregulated DEPs that were suppressed in *Chchd10/Oma1* hearts (relative to *Chchd10*) were DELE1-dependent mtISR factors (Lin et al, 2024; Labbé et al, 2024), including PYCR1, AKR1B7, MTHFD2, PCK2, MTHFD1L, ALDH18A1, GHITM, GPT2, LONP1, GARS1, GATM, and SHMT2 (Fig. EV3I). However, suppression of mtISR signaling was not associated with rescued solubility of mutant CHCHD10: Na_2_CO_3_ extraction studies revealed CHCHD10 to be equally insoluble in *Chchd10* and *Chchd10/Oma1* relative to WT and *Oma1* cardiac mitochondria (Fig. 3E; Appendix Fig. S2), demonstrating that CHCHD10 insolubility can be uncoupled from mtISR induction.

### Inhibition of OMA1 delays cardiomyopathy in *Chchd10* mutant mice

Having generated viable *Chchd10* mutant mice with inactive OMA1 and blunted mtISR, we decided to explore whether OMA1 inactivation modulates mitochondrial dysfunction in *Chchd10/Oma1* hearts. High-resolution respirometry revealed that cytochrome *c* oxidation rates were equivalently diminished in male *Chchd10/Oma1* and *Chchd10* cardiac mitochondria relative to either WT and *Oma1* littermates (Fig. 3F). In line, principal component analyses (PCA) of mitochondrial proteomics showed that *Chchd10* and double mutant mice overlap substantially, indicating similarity in the mitochondrial proteomes of these two groups, which were clearly demarcated from both WT and *Oma1* cardiac proteomes (Fig. EV3J). These data suggest that bioenergetic defects observed at the onset of cardiomyopathy in *Chchd10* mice are not caused by mtISR activation, which is consistent with previous in vitro observations in HEK293T cells expressing insoluble, mutant CHCHD10^G58R^ in which *OMA1* silencing did not rescue mitochondrial respiration (Shammas et al, 2022). TEM analyses of mitochondrial size and cristae content showed that defects in mutant *Chchd10* hearts (Fig. 3G,H) were not rescued by OMA1 inactivation in *Chchd10/Oma1* hearts, despite an inhibition of stress-induced OPA1 processing. We observed a 36% reduction of mtDNA at 14 weeks in both male and female *Chchd10* mutant hearts by qPCR (Fig. 3I) that mirrored the reduction in mtDNA factors such as TFAM, the mtDNA nucleoid regulator whose abundance tracks and controls mtDNA content (Jiang et al, 2017; Kaufman et al, 2007; Larsson et al, 1998), as well as POLG2, POLRMT, TWINKLE, and mtSSB (Fig. 3J). These mtDNA defects were not rescued in *Chchd10/Oma1* mice at 14 weeks of age (Fig. 3I,J), arguing against a role of the mtISR in regulating mtDNA content at the onset of cardiac dysfunction (Sayles et al, 2022). Despite the persistence of structural and functional defects in mitochondria in the hearts of *Chchd10/Oma1* mice, echocardiography revealed an improvement of cardiac function in double mutant mice (Fig. 3K,L): reduced %LVEF was restored to levels indistinguishable from wild-type mice in male mutant mice at 16 weeks of age (Fig. 3L) pointing to a cardioprotective effect of OMA1 inactivation. OMA1 inactivation did not rescue cardiac fibrosis induced by mutant CHCHD10 (Fig. EV3K), and by 35 weeks of age, cardiac function in double mutant mice declined to levels observed in *Chchd10* mutant mice, indicating that OMA1-dependent cardioprotection is temporary (Fig. 3K,L). Of note, both male and female double mutant male and female mice had lifespans and weight gain curves that were similar to *Chchd10* mutant counterparts, suggesting that the molecular mechanisms through which OMA1 inhibition influences organ function may be tissue-specific (Lin et al, 2024; Shammas et al, 2022) (Fig. EV3L,M). Taken together, our data suggest that OMA1 inactivation delays the onset of cardiomyopathy independently of the modulation of cytochrome *c* oxidation, mtDNA content, and cristae structure defects in mutant *Chchd10* mice.

To gain insights into how OMA1 inactivation modulates downstream cardiac signaling, we analyzed cardiac transcriptomes by bulk RNAseq. First, we decided to compare female *Chchd10/Oma1* to female *Chchd10* mice since both have normal cardiac function at 14 weeks, enabling us to identify OMA1-specific pathways that can be modulated in response to mutant CHCHD10. In line with a reduction of ISR signaling, we observed 31 out 67 DEGs in *Chchd10/Oma1* double mutant hearts were involved in ATF-dependent signaling (Fig. EV3N; Dataset EV1), consistent with qRT-PCR studies revealing a reduction in ISR markers (Fig. 3C). Consequently, Enrichr pathway analyses of the 31 upregulated DEGs in *Chchd10* hearts relative to *Chchd10/Oma1* hearts revealed an implication of amino acid metabolism (glycine, serine, threonine, cysteine and methionine), ferroptosis, and folate and one-carbon metabolism (Fig. EV3N; Dataset EV6) and stress signaling pathways associated with PERK, GCN2, ATF and HRI signaling. On the other hand, 55 genes were upregulated in *Chchd10/Oma1* hearts relative to *Chchd10* hearts, which included factors involved in anti-viral and interferon signaling pathways such as *Cxcl9, Cxcl10*, *Cxcl12*, and *Cxcl21a* as well as *Irf7*, *Isg15*, *Oasl1*, *Irgm1*, and *Mx2*. Closer inspection revealed a peculiar albeit limited set of upregulated genes belonging to these clusters, which included IFIT family members (*Ifit1*, *Ifit2*, *Ifit3, and Ifit3b*), which have previously been identified as negative regulators of pathogen-induced NF-kappaB and TNF signaling (Li et al, 2009; John et al, 2018; Kimura et al, 2019). Analysis of Sirius red cardiac histology at 22 weeks of age revealed cardiac fibrosis in *Chchd10/Oma1* and *Chchd10* mice of both sexes. The degree of cardiac fibrosis in male and female *Chchd10* mice showed no sexual dimorphism, suggesting that female hearts may cope better than male hearts in response to mitochondrial dysfunction (Fig. EV3K–M). Indeed, the comparison of DEGs in WT male and female littermates at 14 weeks of age revealed a male-specific upregulation of inflammatory factors *C7* and *Ccl11*, as well as *Nppb* (Dataset EV1), which encodes the Natriuretic Peptide B associated with decreased cardiac output and increased cardiac damage (Goetze et al, 2020) that is consistent with an established, underlying sensitivity of male mice for cardiomyopathy (Lindsey et al, 2024). Altogether, our data indicate that inactivation of OMA1 confers cardioprotection via transcriptional remodeling associated with suppression of the mtISR.

### CHCHD10 insolubility disrupts IMS and IMM proteostasis

**Figure 4:**
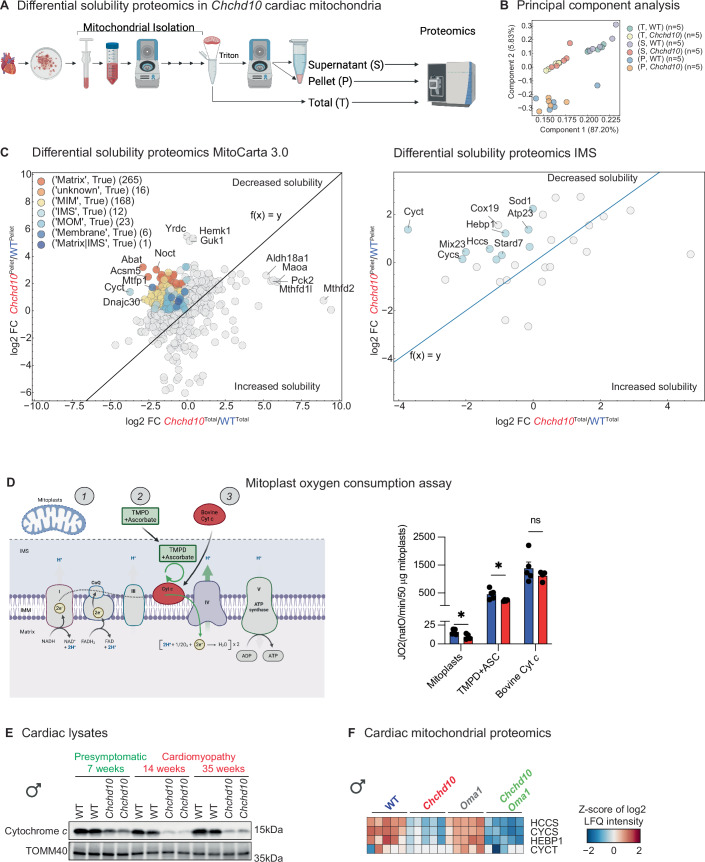
Differential solubility proteomics identifies cytochrome *c* defect. (**A**) Differential solubility proteomic analyses of supernatant (S) and pellet (P) fractions of detergent-solubilized cardiac mitochondria from WT and *Chchd10* male mice at 14 weeks of age. (**B**) Principal component analysis (PCA) of Total (T) input, soluble supernatant (S), and pellet (P) fractions. The experiment was performed on five biological replicates. (**C**) Mitochondrial protein insolubility assessed by measuring protein abundance (Log2FC) measured in the *Chchd10* mutant pellet (P) relative to the WT pellet (*y* axis) plotted against the abundance (Log2FC) measured in the *Chchd10* total input (T) relative to the WT total input (*x* axis) for all MitoCarta 3.0 proteins (left) or IMS proteins (right). The line represents the identity function (fx) = y (e.g., slope 1). Proteins belonging to the matrix, inner membrane (MIM), intermembrane space (IMS), and outer membrane (MOM) according to MitoCarta. Proteins on the left side of the diagonal reflect reduced relative solubility (Dataset EV7). (**D**) Oxygen consumption rates (JO_2_) of mitoplasts from WT (*n* = 5, blue) and *Chchd10* (*n* = 4, red) male mice at 14 weeks. The cartoon represents the workflow (left). JO_2_ measured sequentially in the presence of N,N,N′,N′-Tetramethyl-p-phenylenediamine (TMPD) plus ascorbate (ASC) and bovine cytochrome *c* (Cyt *c*) (right). Data represent mean ± SEM; unpaired Student’s *t* test, Mitoplasts; *P = 0.33667, TMPD + ASC; *P = 0.42028. ns not significant. (**E**) Immunoblots in cardiac lysates of presymptomatic (green) and symptomatic (red) WT and *Chchd10* male mice. Antibodies directed at cytochrome *c* and TOMM40. (**F**) Heatmaps representing cardiac proteomic measurements of Cytochrome c (CYTC and CYCS), HEBP1, and HCCS in the total fraction of wild-type (WT, *n* = 5), *Chchd10* (*n* = 5), *Oma1* (*n* = 5), and *Chchd10/Oma1* (*n* = 5) male mice at 14 weeks of age. The log2 LFQ intensities were Z-Score transformed. Rows were ordered using the complete method, applying Euclidean distance. The dendrogram is not shown. Source data are available online for this figure.

**Figure EV4:**
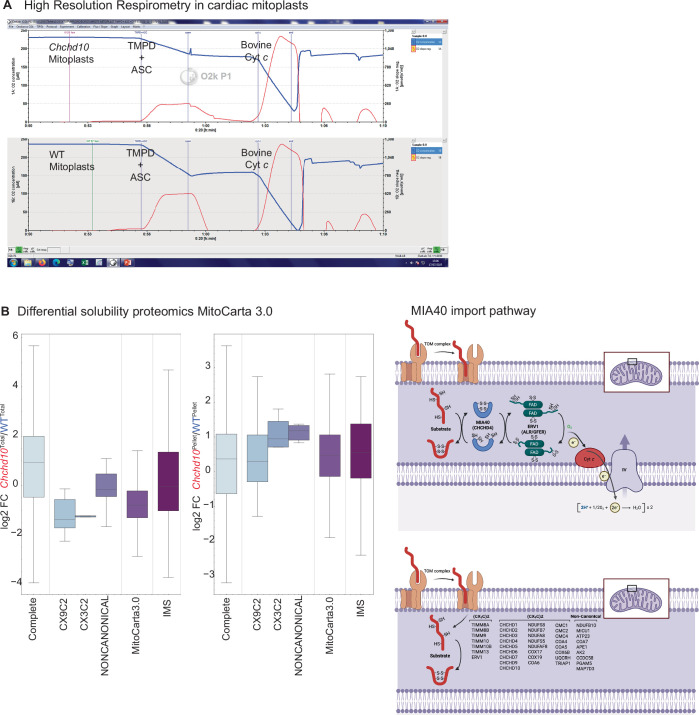
Differential solubility proteomics in *Chchd10* mice. (**A**) Representative traces of oxygen consumption rates (JO_2_) measured in *Chchd10* and wild-type (WT) mitoplasts using High-Resolution Respirometry (Oroboros). Blue trace represents chamber oxygen (O_2_) concentration (μM, left *y* axis) and red trace represents O_2_ consumption (pmol/(s*ml, right *y* axis). JO_2_ measured sequentially following addition of N,N,N′,N′-Tetramethyl-p-phenylenediamine (TMPD) plus ascorbate (ASC) and bovine cytochrome *c* (Cyt *c*) at indicated times (running time in hours: min (h:min), *x* axis). (**B**) Boxplots of MitoCarta, IMS, and MIA40 client types based on differential solubility proteomic analyses of total (T, left) and pellet (P, right) fractions of detergent-solubilized cardiac mitochondria from WT (*n* = 5) and *Chchd10* (*n* = 5) male mice at 14 weeks of age (left, Dataset EV7). Box edges display the 25% (lower) and 75% (upper) quartile. The whiskers display the highest and lowest non-outlier value. Outliers (1.5 x inter quartile range from box edge) are not shown. The black line in the box indicates the median (50%). Cartoon representation of MIA40/ERV1 import pathway (top) in which client proteins (Al-Habib and Ashcroft, 2021) are indicated (right).

Having identified downstream signaling pathways triggered by mitochondrial dysfunction in the hearts of *Chchd10* mutant mice, we focused our attention back to the molecular defects underpinning cytochrome *c* oxidation. At first, the most parsimonious explanation was an enzymatic defect in Complex IV, which was associated with reduced copper and Complex IV supercomplex levels (Fig. 2L). Yet, the approximately 50% reduction in cytochrome *c* oxidation rates measured by high-resolution respirometry was associated with a more modest 31% and 21% decrease in mitochondrial copper content in males and females, respectively (Fig. 2N). Similarly, recent studies have called into question the functional relevance of impaired respiratory chain supercomplex (RCS) assembly for cardiac homeostasis under basal conditions (Milenkovic et al, 2023). Cardiac mitochondrial proteomics revealed that most DEPs associated to Complex IV were reduced (Fig. 2G,I), yet pairwise comparisons of *Chchd10* and WT hearts revealed an unexpected increase in the levels of COA4 and COA7, both of which are twin Cx(9)C motif IMS proteins that are functionally linked to Complex IV via the handling of mitochondrial copper (Swaminathan et al, 2022; Formosa et al, 2022) (Fig. 2I). As the IMS import, folding, and activity of these proteins is reliant on MIA40/CHCHD4 pathway and given that MIA40/CHCHD4 levels were also found to be increased in *Chchd10* mutant mice (Fig. 2G; Dataset EV5), we wondered if CHCHD10 insolubility might influence the insolubility other mitochondrial proteins relevant for cardiac health. Therefore, we performed differential solubility proteomics on *Chchd10* cardiac mitochondria. Solubilizing cardiac mitochondrial lysates in detergent followed by differential centrifugation enabled us to separate detergent-soluble supernatant fractions from detergent-insoluble pellet fractions. These fractions along with the initial non-fractionated total input were subsequently analyzed by mass spectrometry (Fig. 4A). PCA distinguished WT and *Chchd10* total and supernatant fractions based on genotype, but not the pellet fraction (Fig. 4B). Therefore, to identify insoluble candidate proteins, we plotted mitochondrial proteins in the insoluble pellet fraction relative to their abundance measured in the total input of *Chchd10* mutant mice, which allowed us to correct for relative decreases of individual mitochondrial proteins and avoid that highly upregulated proteins be improperly ascribed to the insoluble pellet fraction simply based on their altered expression in the total input (Fig. 4C). In so doing, we observed a wide range of proteins from various mitochondrial subcompartments whose relative solubility were reduced using this metric (Fig. 4C), including MTFP1 and cytochrome *c*. MTFP1 is an IMM protein that regulates inner membrane integrity and bioenergetic efficiency of cardiomyocytes(Donnarumma et al, 2022). While it is essential for adult cardiac function, its ablation in cardiomyocytes does not phenocopy the bioenergetic mitochondrial defects nor the cardiac defects observed in *Chchd10* hearts (Donnarumma et al, 2022). Cytochrome *c* is a soluble, dual-function protein that acts as an electron donor to Complex IV at the IMM but also triggers caspase activation and apoptosis when it is released from mitochondria to the cytosol (Garrido et al, 2006). In cardiac mitochondria from *Chchd10* mutant mice, total cytochrome c levels were decreased by >fivefold (Log2FC = −2392, Dataset EV7), while the relative levels in the insoluble pellet fraction increased (Fig. 4C). To determine whether cytochrome *c* dysfunction contributed to the reduced oxygen consumption rates we observed in isolated cardiac mitochondria supplied with Antimycin A, TMPD, Ascorbate, and CCCP (Figs. 2O and 3F), we repeated high-resolution respirometry measurements with mitoplasts generated from wild-type and *Chchd10* cardiac mitochondria to which TMDP, and Ascorbate were added, observing once again a significant impairment of oxygen consumption rates. Subsequent addition of exogenous bovine cytochrome *c* to immediately increased oxygen consumption rates in wild-type and *Chchd10* mitoplasts alike and to statistically indistinguishable levels (Fig. 4D), arguing that cytochrome *c* deficiency in mitochondria from *Chchd10* mice is responsible for impaired cytochrome *c* oxidation, at least in isolated mitoplasts assayed in vitro. In this assay, increased oxygen flux occurred within a matter of seconds following injection of bovine cytochrome *c* into the respirometry chambers (Fig. EV4A), arguing against an indirect increase due to mtDNA gene expression indirectly stimulated by cytochrome *c*. To test whether *Chchd10* mice suffer from an intrinsic cytochrome *c* deficiency, we analyzed cardiac lysates by immunoblot, which revealed a profound decrease in cytochrome *c* in both symptomatic and presymptomatic mice (Fig. 4E) that paralleled the kinetics of CHCHD10 insolubility and accumulation and ISR signaling (Fig. EV3A). Transcriptomic analyses of *Chchd10* in mutant hearts did not reveal a decrease in cytochrome *c* mRNA levels (Dataset EV1) that could explain reduced steady-state protein levels, instead implying defects in the import and/or stability of cytochrome *c*.

Cytochrome *c* is synthesized in the cytoplasm as apocytochrome c, lacking heme, and its import into the IMS is inexorably linked to the covalent attachment of heme at the CXXCH motif by the heme lyase Holo-Cytochrome C-type Synthase (HCCS) (San Francisco et al, 2013). The steady-state levels of HCCS and the heme-binding protein (HEBP1), which has been associated with mitochondrial dysfunction in rodents (Yagensky et al, 2019), were reduced in *Chchd10* and *Chchd10/Oma1* cardiac mitochondria (Fig. 4F) that showed reduced mitochondrial respiration rates in the presence of Antimycin A, TMPD, Ascorbate, and CCCP (Fig. 3F). Filtering the differential solubility proteomic data on the basis of IMS proteins revealed that MIA40 clients such as COX19, SOD1, ATP23 and MIX23, and were disproportionally affected by mutant CHCHD10 (Fig. 4C), consistent with a defect in IMS proteostasis and/or biogenesis (Fig. EV4B). Altogether, our data implicate defects in cytochrome *c* biogenesis in the impairment of mitochondrial respiration caused by mutant CHCHD10, revealing an early bioenergetic defect contributing to the onset of cardiac dysfunction in *Chchd10* mutant mice.

## Discussion

A defining feature of the *CHCHD10*^*S59L*^ human mutation (*Chchd10*^*S55L*^ in mice) is its impact on the insolubility and accumulation of CHCHD10 within mitochondria, which is associated with a constellation of pleiotropic mitochondrial defects often observed in other mitochondrial disease (MD) animal models and patient-derived biopsies (Anderson et al, 2019; Genin et al, 2019; Southwell et al, 2024; Sayles et al, 2022; Shammas et al, 2022; Lin et al, 2024; Baek et al, 2021). These defects include mtDNA depletion, cristae dysmorphology, mitochondrial fragmentation, impaired proteostasis, and activation of the OMA1–DELE1–HRI axis of the mitochondrial integrated stress response (mtISR). The convergence of multiple, often overlapping mitochondrial defects is a recurrent feature across diverse MD preclinical animal models and patient-derived samples, complicating both molecular diagnosis and therapeutic development. Dissecting which abnormalities represent primary pathogenic drivers versus downstream adaptations is therefore essential to establish causal mechanisms and to guide the design of mechanism-based interventions. Our study provides new insights into the mechanisms through which insoluble CHCHD10 promotes mitochondrial and cardiac dysfunction in a *Chchd10*^*S55L/+*^ mutant mice by (i) defining the functional relevance of mtISR signaling through the catalytic inhibition of OMA1 and (ii) uncovering a novel, reversible early-onset defect in cytochrome *c* oxidation. These findings challenge prevailing models in which mitochondrial bioenergetic collapse is considered a downstream consequence of chronic mtISR signaling (Anderson et al, 2019; Sayles et al, 2022), instead positioning defective intermembrane space (IMS) proteostasis and cytochrome *c* deficiency as early and previously underappreciated triggers for pathogenic cardiac remodeling.

We adapted a strategy previously used to identify insoluble proteins resulting from the perturbation of mitochondrial proteostasis in yeast (Baker et al, 2012) that we coupled to mass spectrometry to perform differential solubility proteomic approach, which enabled us to identify a subset of mitochondrial proteins with increased insolubility in *Chchd10* mutant mitochondria, implicating disruption of redox-regulated IMS protein import machinery governed by the MIA40/ERV1 axis (Fig. 4). Among these, cytochrome *c* emerges as a critical node: it is insoluble in *Chchd10* female and male mutant hearts, and its levels are reduced in presymptomatic mutant mice as are HCCS, which is needed for cytochrome *c* biogenesis and stability (Lill et al, 1992; San Francisco et al, 2013; Babbitt et al, 2015). Despite the critical role of cytochrome *c* in programmed cell death, we observed negligible effects of the *Chchd10*^*S55L*^ allele on cell death sensitivity in mouse embryonic fibroblasts (MEFs, Appendix Figs. S3 and S4) nor in vivo (Appendix Fig. S5). Strikingly, supplementation with exogenous bovine cytochrome *c* rescues impaired oxygen consumption rates in cardiac mitoplasts isolated from *Chchd10*^*S55L/+*^ mutant mice (Fig. 4). This finding implicates disrupted cytochrome *c* biogenesis as a key contributor to mitochondrial respiratory collapse, which we show occurs at the onset of cardiomyopathy rather than as a late-stage event (Anderson et al, 2019; Sayles et al, 2022). It will be important to determine whether this paradigm is also relevant for the late-onset neuromuscular defects described in *Chchd10* mice (Genin et al, 2019, 2022, 2024).

The bioenergetic relevance of cytochrome *c* oxidation in Amyotrophic Lateral Sclerosis (ALS) was recently highlighted by studies revealing that the genetic disruption of Cytochrome *c* Oxidase (Complex IV) specifically in neurons is sufficient to recapitulate molecular, cellular, and neuromuscular features of sporadic ALS, including motor neuron death, neuroinflammation, and muscle wasting (Cheng et al, 2025). Convergent clinical and phenotypic manifestations of impaired cytochrome *c* oxidation due to independent cytochrome *c* or Complex IV defects have been documented in other forms of MD, such as microphthalmia with linear skin lesions (MLS)—a developmental syndrome characterized by defects of the neuromuscular, cardiovascular, and skin systems arising from mutations in either *HCCS* or *COX7B*, a structural subunit of Complex IV (Indrieri et al, 2012, 2013). We suspect that other examples of phenotypic overlap caused by such biochemical convergence have yet to be uncovered in other types of MD, which are notorious for their clinical and biochemical heterogeneity (Russell et al, 2020).

Beyond cytochrome *c*, we identify additional mitochondrial proteins with altered solubility that may contribute to the aforementioned mitochondrial dysfunctions. These include ABAT (Besse et al, 2015), YRDC (Lin et al, 2018), RMND1 (Janer et al, 2012), and MRPS16 (Miller et al, 2004)—factors involved in mtDNA stability, replication, and gene expression, and mutated in genetic diseases. Reduced levels of mtDNA replication and maintenance proteins coincide with reduced mtDNA content measured at the onset of cardiomyopathy in males (14 weeks). Neither of these defects is rescued by blunting the mtISR via OMA1 inactivation in *Chchd10*^*S55L/+*^*Oma1*^*E324Q/E324Q*^ mice (Fig. 3I,J), suggesting that mtISR induction is not the primary driver of altered mtDNA homeostasis at the early stages of cardiac dysfunction.

Not all identified proteins that exhibit reduced solubility are classical IMS residents, and thus their altered solubility may reflect broader proteostasis stress across mitochondrial compartments, which could be the consequence of altered mitochondrial ultrastructure and morphology (Wai, 2024) (Fig. 3G,H). CHCHD10^S55L^ aggregation may exert a ripple effect across mitochondrial homeostasis leading to the proteomic remodeling characterized by reduced levels of most proteins in isolated cardiac mitochondria. Alternatively, proteomic remodeling may directly contribute to mitochondrial membrane remodeling via the impaired assembly or maintenance of the MICOS complex (Fig. 2K,M), which was previously implicated in CHCHD10^S59L^-mediated cristae dysfunction (Ropert et al, 2024; Genin et al, 2018). Indeed, future studies are needed to dissect the individual contributions of mtDNA depletion, cristae dysmorphology, and impaired cytochrome *c* oxidation to cardiac function. This will be difficult given the interdependent nature of these mitochondrial processes (Daumke and van der Laan, 2025), yet emerging chemical approaches that target specific mitochondrial dysfunctions may prove to be useful in this endeavor (Bonekamp et al, 2020; Ropert et al, 2024; Wang et al, 2012; Franco et al, 2016).

At the outset, *Chchd10* mutant mice were developed to study the impact of the S59L pathogenic variant in *CHCHD10* patients suffering from autosomal dominant ALS and Frontotemporal Dementia (FTD) (Anderson et al, 2019; Genin et al, 2019). While there is little clinical evidence to support the existence of cardiac defects in the few *CHCHD10*^*S59L/+*^ patients reported thus far, it is possible that subclinical, underlying molecular defects similar to those we and others reported in presymptomatic *Chchd10*^*S55L/+*^ mutant cardiac mitochondria have yet to be discovered. Indeed, several MD nuclear genes (e.g., *SCO2, ANT1, SUCLA2, COX10, COX15*) that are mutated in patients with neurological abnormalities can also lead to cardiomyopathy (Tarnopolsky et al, 2004; Papadopoulou et al, 1999; Palmieri et al, 2005; Kaukonen et al, 2000; Jaberi et al, 2013; Carrozzo et al, 2007; Valnot et al, 2000a; Antonicka et al, 2003a; Oquendo et al, 2004; Antonicka et al, 2003b), underscoring the broader and evolving spectrum of clinical manifestations associated with MD. In *Chchd10*^*S55L/+*^ mice, our findings confirm several previously described mitochondrial and cardiac dysfunctions caused by the toxic gain-of-function CHCHD10^S55L^ protein. However, several discrepancies with previous studies stand out, which could be explained by differences in the mouse genetic backgrounds on which mutant mice were generated. In our study, we used heterozygous *Chchd10*^*S55L/+*^ mice maintained on a C57BL/6N (N) background, whereas studies from the Manfredi and Narendra labs used the C57BL/6J (J) strain (Sayles et al, 2022; Southwell et al, 2024; Anderson et al, 2019). Genetic variations between these two sub-strains of C57Bl/6 mice are known to influence mitochondrial metabolism and cardiac health (Nickel et al, 2015; Close et al, 2021). On the J-strain, cardiomyopathy onset occurs around 18 weeks in male *Chchd10*^*S55L/+*^ mice, with mtDNA depletion and OXPHOS defects reported at 1 year (Anderson et al, 2019; Sayles et al, 2022). In line, double deletion of *Chchd10* and *Chchd2*, which phenocopies mitochondrial defects observed in *Chchd10*^*S55L/+*^ mice, causes cardiac dysfunction on the J-strain with kinetics similar to that of *Chchd10*^*S55L/+*^ mice maintained on the same genetic background (Liu et al, 2020). By contrast, cardiomyopathy manifests in *Chchd10*^*S55L/+*^ mice on the N strain one month earlier (Genin et al, 2019) (Fig. 1B,C) concomitant with the depletion of mtDNA and impaired respiration. J-strain *Chchd10*^*S55L/+*^ mice exhibit iron deficiencies (Sayles et al, 2022) while N-strain *Chchd10*^*S55L/+*^ mice do not (Fig. EV2G), although it should be noted that the techniques used to measure biological metals (including iron) were different. Another divergence from prior studies is the emergence of sex-specific phenotypes in our model. Cardiac phenotyping studies on the J strain focused exclusively on females (Anderson et al, 2019) or males (Southwell et al, 2024), making it challenging to assess the influence of biological sex. However, in the single study of *Chchd10*^*S55L/+*^ mice that included both sexes, Genin and coworkers reported that female mutants had left ventricular ejection fraction (%LVEF) values similar to wild-type male littermates at 23 weeks (Genin et al, 2019). In line, we observe cardiac protection in *Chchd10* females (Fig. 1B,C) and sexually dimorphic effects in *Chchd10*^*S55L/+*^ mice on the N strain including a male-specific increase in cardiac inflammatory pathways. Environmental context may also contribute to differences observed between groups studying *Chchd10*^*S55L/+*^ mice. Animal housing conditions, diet, and microbiota composition are increasingly recognized as critical modifiers of phenotype onset and severity (Franklin and Ericsson, 2017), which is likely relevant for mitochondrial disorders (Zachos et al, 2024). Recent work from the Manfredi group identified high-fat diet feeding as a nutritional intervention capable of alleviating mtISR and cardiomyopathy in male mutant mice by restoring CHCHD10 solubility to the aggregation-prone S55L variant (Southwell et al, 2024), which can also suppress cardiomyopathy in cardiomyocyte-specific *Yme1l1* knockout mice (*Yme1l1*^*Heart*^) (Wai et al, 2015). These observations underscore the need to consider both genetic and non-genetic modifiers in interpreting preclinical disease models.

Finally, functional suppression of STING and OMA1 in *Chchd10*^*S55L/+*^ mutant mice has provided insights into the relevance of overactive innate immune signaling and mtISR, respectively. The beneficial effects of STING ablation we observe likely reflect the general protection against overactive innate immune signaling triggered by mitochondrial dysfunction, which has been previously documented in mouse models for *Polg*, *Tfam*, *Pink1*, *Parkin* (Lei et al, 2021; Chung et al, 2019; Sliter et al, 2018) and in mice subjected to chemically-induced mitochondrial dysfunction (Yu et al, 2020; Lei et al, 2023). For OMA1, our findings refine the understanding of the physiological relevance of the stress-induced metalloprotease in CHCHD10 pathogenesis. In the hearts of *Chchd10*^*S55L/+*^ mice, OMA1 inactivation transiently preserves cardiac function without ultimately rescuing OXPHOS defects, mtDNA levels, or CHCHD10 protein solubility. This suggests that ISR suppression alone is insufficient to correct the primary energetic defect in the heart and instead supports a paradigm in which CHCHD10 aggregation and proteostatic dysfunction are the proximal triggers of mitochondrial dysfunction in the heart. Transcriptomic analyses reveal that OMA1 inhibition does not suppress overactive innate immune signaling in *Chchd10*^*S55L/+*^ *Oma1*^*E324Q/E324Q*^ mutant hearts (Dataset EV6), which is consistent with the breadth of cardioprotection we observe. On the other hand, in skeletal muscle of mutant mice modeling the *CHCHD10*^*G58R*^ allele, OMA1 deletion is synthetically lethal and exacerbates myopathy (Lin et al, 2024). We posit that these discrepancies stem from the nature of the toxic, gain-of-function CHCHD10 mutation (G58R versus S59L) and the affected tissue (skeletal muscle versus heart), rather than the difference between the deletion of *Oma1* and the catalytic inactivation by the *Oma1*^*E324Q/E324Q*^ model, since the *Oma1*^*−/−*^ and *Oma1*^*E324Q/E324Q*^ cardiac proteomes are no different (Fig. EV3D) (Ahola et al, 2022). A previous study exploring the phenotypic effects of suppressing the mtISR through *Dele1* or *Oma1* ablation in *CHCHD10*^*G58R*^ mutant mice revealed that *Dele1* deletion shortens the lifespan of mutant mice more substantially than *Oma1* deletion. Differential effects between the aforementioned G58R versus S59L variants notwithstanding, it remains an open possibility that either *Dele1* has additional, non-redundant functions that are epistatic to the *CHCHD10*^*G58R*^ allele and/or that *Dele1* deletion more strongly suppresses the activation of the mtISR than does the deletion of *Oma1* (Lin et al, 2024). If the cardioprotection observed in *Chchd10*^*S55L/+*^ *Oma1*^*E324Q/E324Q*^ mutant hearts is mediated exclusively via mtISR suppression, one would predict that *Dele1* deletion in the *Chchd10*^*S55L/+*^ mutant mice should confer equivalent, or potentially greater, cardioprotection than that seen in *Chchd10*^*S55L/+*^ *Oma1*^*E324Q/E324Q*^ mutant mice. Indeed, future studies are needed to explore these questions as well as the breadth of protection offered by the *Oma1*^*E324Q/E324Q*^ model, notably in the neuromuscular system of *Chchd10*^*S55L/+*^ mice and other MD mouse models of intramitochondrial protein aggregation and proteostatic imbalance.

## Methods

**Table:** Reagents and tools table

Reagent/resource	Reference or source	Identifier or catalog number	
**Experimental models**			
C57Bl6/N	Charles River		
*Chchd10*^S55L/+^ (Chchd10tm1.1Vpf)	Genin et al, 2019		
*Oma1* ^*E324Q/E324Q*^	This study	N/A	
*Sting* ^*Gt/Gt*^	Jackson Lab	IMSR_JAX:017537	
*Yme1l*^*Heart*^ (Tg(Myh6-cre)2182Mds and Yme1l1^tm1Tlan^)	Wai et al, 2015		
Primary MEFs (wild-type or Chchd10S55L/+ (Chchd10tm1.1Vpf)	This study	N/A	
**Recombinant DNA**			
**Antibodies**			
OPA1	612607	BD Biosciences	
MTCO2	55070-1-AP	Proteintech	
LC3	12135-1-AP	Proteintech	
TOM40	18409-1-AP	Proteintech	
IMMT	10179-1-AP	Proteintech	
ANT1	ab110322	Abcam	
NPPA	27426-1-AP	Proteintech	
CHCHD2	66302-1-Ig	Proteintech	
P62	18420-1-AP	Proteintech	
MTHFD2	12270-1-AP	Proteintech	
Vinculin	26520-1-AP	Proteintech	
CHCHD10	25671-1-AP	Proteintech	
Anti-cytochrome c	556433	BD Pharmigen	
NDUFA9	ab14713	Abcam	
SDHA	459200	Invitrogen	
COX IV	ab14744	Abcam	
ATP5A	ab14748	Abcam	
UQCRC2	ab14745	Abcam	
HSP60	15282-1-AP	Proteintech	
Anti-rabbit HRP-Conjugated	a120-101p	Bethyl Laboratories	
Anti-mouse HRP-Conjugated	a90-116p	Bethyl Laboratories	
**Oligonucleotides and other sequence-based reagents**			
**Gene**	**Primers**	**Sequence**	**Target**
Gapdh	TW145	AGGTCGGTGTGAACGGAT	RT qPCR
Gapdh	TW146	GGGGTCGTTGATGGCAACA	
Cxcl10	TW791	CCAAGTGCTGCCGTCATTTTC	RT qPCR
Cxcl10	TW792	GGCTCGCAGGGATGATTTCAA	
Fgf21	TW1168	ctatcatcctgagactgggcag	RT qPCR
Fgf21	TW1169	TCGTCTTTGTAGTCcttcacgg	
*16 s rRNA*	TW764	CCGCAAGGGAAAGATGAAAGAC	qPCR mtDNA
*16 s rRNA*	TW765	TCGTTTGGTTTCGGGGTTTC	
*MT-ND1*	TW766	CTAGCAGAAACAAACCGGGC	qPCR mtDNA
*MT-ND1*	TW767	CCGGCTGCGTATTCTACGTT	
Beta Actin	TW392	GTGACGTTGACATCCGTAAAGA	qPCR nDNA
Beta Actin	TW393	cctcaccaagctaaggatgc	
Atf4	TW833	CTCATGGGTTCTCCAGCGACAAG	RT qPCR
Atf4	TW834	GTCAAGAGCTCATCTGGCATGG	
Atf5	TW835	GAGGGAGGTCTCGTGTACGTCTG	RT qPCR
Atf5	TW836	GCTTTCTCAGTTGCACTGAAGGGG	
Phgdh	TW837	GACCCCATCATCTCTCCTGA	RT qPCR
Phgdh	TW838	GCACACCTTTCTTGCACTGA	
Trib3	TW841	TCGCTTTGTCTTCAGCAACTGTGAG	RT qPCR
Trib3	TW842	CATCAGCCGCTTTGCCAGAGTAG	
Mthfd2	TW843	CATGGGGCATATGGGAGATAAT	RT qPCR
Mthfd2	TW844	CCGGGCCGTTCGTGAGC	
Asns	TW845	CTGCTTTGGCTTTCACCGCTTG	RT qPCR
Asns	TW846	TGCTGTAGCGCCTTGTGGTTG	
Aldh18a1	TW1170	AATCAGGGCCGAGAGATGATG	RT qPCR
Aldh18a1	TW1171	GGCCTCTAAGACCGGAATTGC	
Psat1	TW1172	AGTGGAGCGCCAGAATAGAA	RT qPCR
Psat1	TW1173	CTTCGGTTGTGACAGCGTTA	
**Chemicals, enzymes, and other reagents**			
NaCl	1112-A	Euromedex	
NaCl	Euromedex	1112-A	
KCl	Euromedex	P017	
KH_2_PO_4_	Sigma-Aldrich	P5379	
NaH_2_PO_4_	Thermo Scientific	10284010	
HEPES	Euromedex	10-110	
BDM (2,3-Butanedione monoxime)	Sigma-Aldrich	B0753	
Taurine	Sigma-Aldrich	T0625	
MgCl_2_	Sigma-Aldrich	M7506	
EDTA	Euromedex	EU0084-B	
Collagenase II	Thermo Scientific	17101015	
Collagenase IV	Thermo Scientific	17104019	
Protease XIV	Sigma-Aldrich	P5147	
FBS	Thermo Scientific	10270106	
Phosphate-buffered saline	Gibco	14190169	
Laminin	Thermo Scientific	23017-015	
M199 Medium	Sigma-Aldrich	M4530	
Bovine serum albumin, fatty acid-free	Sigma-Aldrich	A6003	
ITS supplement	Sigma-Aldrich	41400045	
Chemically defined lipid concentrate	Thermo Scientific	11905-031	
Penicillin–streptomycin	Thermo Scientific	15070063	
Glucose	Sigma-Aldrich	UG3050	
TMPD (N,N,N′,N′-Tetramethyl-p-phenylenediamine dihydrochloride)	Sigma-Aldrich	87890	
Carbonyl Cyanide m-chlorophenyl hydrazine	Sigma-Aldrich	C2759	
Rotenone	Sigma-Aldrich	R8875-1G	
Antimycin A	Sigma-Aldrich	A8674	
Potassium cyanide	Sigma-Aldrich	60178-100 G	
Oligomycin	Sigma-Aldrich	O-4876	
Seahorse XF base medium	Agilent Technologies	102353-100	
DMEM W/GLUTAMAX-I, PYR, 4.5 G	Life Technologies	31966047	
Triton X-100	EUROMEDEX	2000-C	
Sodium deoxycholate	Sigma-Aldrich	D6750-100G	
Sodium dodecyl sulfate	EUROMEDEX	1833	
EGTA	Sigma-Aldrich	E4378-10G	
Quick Start Bradford 1× Dye Reagent	BIORAD	5000205	
Bovine serum albumin	Dominique Dutscher	P06-1391100	
Laemmli sample buffer 4×	BIORAD	1610747	
2-mercaptoethanol	Sigma-Aldrich	M3148-100ML	
TBS 10x	Euromedex	ET220-B	
Clarity™ Western ECL Substrate	BIORAD	1705061	
Sucrose	Euromedex	200-301-b	
Trypsin-EDTA	Pasteur-Dutscher	X0930-100	
cOmplete™, EDTA-free Protease Inhibitor Cocktail	Sigma-Aldrich	4693132001	
Protease K			
TRICHLOROACETIC ACID BIOXTRA	Sigma-Aldrich	T9159-500G	
TRIzol™ Reagent	THERMO FISHER	15596026	
Chloroform	Sigma-Aldrich	372978-2 L	
iTaq™ Universal SYBR® Green Supermix, 2,500 ×20 µl rxns, 25 ml (5 ×5 ml)	BIORAD	1725124	
Sodium pyruvate	Thermo Fisher	11530396	
L-glutamine	Sigma-Aldrich	G8540-25G	
Lactobionic acid	Sigma-Aldrich	153516-25 G	
Rhodamine	Thermo Fisher	R302	
L-(−)-Malic acid	Sigma-Aldrich	M 1000	
Palmitoyl-L-carnitine chloride	Sigma-Aldrich	P1645-25MG	
Adenosine 5′-diphosphate monopotassium salt dihydrate	Sigma-Aldrich	A5285-1G	
Sodium succinate dibasic hexahydrate, ReagentPlus®, >=99%	Sigma-Aldrich	S2378-100G	
(+)-Sodium L-ascorbate	Sigma-Aldrich	A4034-100G	
Cytochrome c	Sigma-Aldrich	C7752-100MG	
PIPES sodium salt	Sigma-Aldrich	P2949	
Glutaraldehyde	Sigma-Aldrich	G5882-10X10ML	
MS 32% paraformaldehyde aqueous solution, EM Grade	VWR	100496-496	
H2O2 30%	Sigma-Aldrich	H1009-100ML	
Cyclosporin A	Sigma-Aldrich	30024-25MG	
Isoproterenol	Sigma-Aldrich	I6379	
Indomethacin	Sigma-Aldrich	57413	
MOPS	Sigma-Aldrich	M1254-25G	
Potassium acetate	Euromedex	1131-B	
Glycerol	Life Technologies Technologies	15514011	
6-aminohexanoic	Sigma-Aldrich	7260	
Digitonin	Sigma-Aldrich	D141-500MG	
NativePAGE™ 5% G-250 Sample Additive	Life Technologies	BN2004	
NativePAGE™ 3–12% Bis-Tris Protein Gels, 1.0 mm, 10-well	Life Technologies	BN1001BOX	
Cathode buffer additive	Life Technologies	BN2002	
mPAGE™ 8% Bis-Tris Protein Gels, 10×8, 15-well	Millipore	MP8W15	
Seahorse XFe96 FluxPak	Agilent Technologies	102601-100	
Trans-blot Turbo RTA Transfer kit, Nitrocellulose	BIORAD	1704271	
NucleoSpin RNA	Macherey-Nagel	740955.25	
Nucleospin Tissue	Macherey-Nagel	740952.250	
Pierce™ BCA® Protein Assay Kits and Reagents, Thermo Scientific, BCA Protein Assay Kit	Life Technologies	23225	
Cardiac Troponin I Elisa	Life Diagnostics	CTNI-1-US	
**Software**			
Vevo Lab	VisualSonics		
Fiji (ImageJ) v1.53	NIH		
QuPath	Bankhead et al, 2017		
Image Lab Software v.6.1.0	Bio-Rad		
Datlab v.7	Oroboros		
Harmony v.4.9	PerkinElmer Life Sciences		
Prism v10	GraphPad		
**Other**			
Vevo 3100 Imaging System	VisualSonics		
AxioScan.Z1	Zeiss		
ChemiDoc Gel Imaging System	Bio-Rad		
High-Resolution Respirometry system (O2k-Fluorespirometer)	Oroboros		
Easy-LC 1200	Thermo Fisher Scientific		
Exploris 480 mass spectrometer	Thermo Fisher Scientific		
ICP-OES; Optima 7300DV	PerkinElmer Life Sciences		
Illumina NextSeq 500	llumina		
Illumina NextSeq 2000	Illumina		
NanoQuant Plate (Infinite M200)	Tecan		
TECNAI T12 Transmission Electron Microscope (FEI)	Thermo Fisher Scientific		
Operetta CLS High-Content Analysis system	PerkinElmer Life Sciences		

### Animals

Animal care was conducted in accordance with European animal welfare laws (Directive 2010/63/EU). The French Ministry of Research and local Animal Ethics Committees reviewed and approved all experiments under the authorized protocol APAFIS #32988-2021091417302194. Mice were kept in a specific pathogen-free environment with standard conditions, including a 14-h light/10-h dark cycle, 50-70% humidity, and a temperature range of 19–21 °C. They had unlimited access to food and water in cages equipped with bedding and gnawing sticks for enrichment. The principles of the 3Rs were followed to meet animal welfare standards. Animals were checked weekly, and euthanasia was performed to minimize pain and distress if body weight loss exceeded 20%. The generation of *Chchd10*^*S55L/+*^ mice was described previously (Genin et al, 2019). *Oma1*^*E324Q/E324Q*^ mice were generated using CRISPR/Cas9 endonuclease-mediated genome editing on the C57Bl6/N background. The sgRNA 5’-GTGCGATCTCATGGCCCAGG *AGG*-3’ was designed using the CRISPOR Web tool (http://crispor.tefor.net/) (Brownstein, 2003).

The donor matrix chosen was a 161-nucleotide ssODN:

(GAATGGACAAGTGTTTATTTTCACCGGGCTTCTGAATAGTGTGACGGACGTGCACCACTGTCCTTtCTCCTGGGCCATcAGATCGCACACGCAGTCCTGGGGCACGCCGTGAGTACCGGGATGTGCAGCTGCTGAATGTTTGCTTTGATATTAGCAAGTG).

For transgenesis experiments, 3-week-old C57BL/6N females were superovulated and mated with C57BL/6N stud males to recover one-cell embryos. Pronuclei of fertilized embryos were co-microinjected with RNP CRISPR/Cas9 complex (20 ng/μL Cas9 protein, 20 ng/μL sgRNA) together with 20 ng/μl of ssODN (all reagents from Integrated DNA Technologies, IDT). Microinjected embryos were then implanted into the oviducts of C57BL/6 J×CBA F1 foster mothers following standard procedures (Brownstein, 2003).

F0 Mutant mice were screened by PCR and confirmed by direct sequencing (Haeussler et al, 2016), followed by germline transmission analysis and confirmation of the descendants for mutational insertions and off-target effects. *Chchd10*^*S55L*^ *Oma1*^*E324Q/E324Q*^ were backcrossed on C57Bl6/N background. *Sting*^*Gt/Gt*^ mice (IMSR_JAX:017537) and *Chchd10*^*S55L*^ *Sting*^*Gt/Gt*^ mice were intercrossed and backcrossed on the C57Bl6/N background. Cardiomyocyte-specific *Yme1l1* knockout mice (*Yme1l*^*Heart*^) (Wai et al, 2015) were rederived on a C57Bl6/N background.

### Echocardiography

Transthoracic echocardiography was conducted using a Vevo 3100 Imaging System paired with a 25–55 MHz linear-frequency transducer (MX550D, FUJIFILM VisualSonics). Randomized WT, *Chchd10*^*S55L/+*^, *Oma1*^*E324Q/E324Q*^, *Chchd10*^*S55L/+*^*Oma1*^*E324Q/E324Q*^, *Sting*^*Gt/Gt*^, and *Chchd10*^*S55L/+*^*Sting*^*Gt/Gt*^ mice were anesthetized with 2% isoflurane in oxygen and positioned supine on a 37 °C heated pad. Limb electrodes and a rectal probe monitored the ECG and body temperature. Prior to echocardiography and applying ultrasound gel, the fur on the thorax was removed with hair-removal cream. To evaluate left ventricle (LV) size and function, B- and M-Mode images were captured in the parasternal long-axis view (PLAX) at a heart rate of 400–500 bpm. Measurements of the systolic and diastolic LV dimensions, including interventricular septum thickness (IVS; mm), LV diameter (LVD; mm), LV posterior wall thickness (LVPW; mm), and cardiac output [ejection fraction (% LVEF)], were obtained by analyzing at least three independent cardiac cycles across at least three M-Mode images using Vevo Lab (VisualSonics), as previously described (Donnarumma et al, 2022).

### Histology

Mice were euthanized through cervical dislocation, and their hearts were removed post-mortem. The entire hearts were fixed in 4% formaldehyde (VWR chemicals) overnight and then fully dehydrated using a series of ethanol gradients. The tissues were then embedded in paraffin and sectioned into 4-μm-thick slices using a microtome. These sections were deparaffinized in xylene, rehydrated, and subsequently stained with hematoxylin and eosin (H&E) or Picrosirius Red (ABCAM, ab150681) according to standard protocols. The images were captured using a slide scanner AxioScan.Z1 (ZEISS). Histopathological evaluation was performed using digitized whole-slide images and the QuPath software (Bankhead et al, 2017). Images were analyzed using Fiji (ImageJ) by quantifying the area in red as fibrosis area and normalized by the total area of the tissue section using a macro. All analysis settings and annotations were stored within the same project. The annotation tool was used to segment the tissue area for each image. To determine the extent of fibrosis, we used QuPath’s machine learning-based object classifier. Annotation classes were defined for both fibrosis and background areas. For model training, three representative cropped regions were selected from each image included in the analysis, and a composite training set was created. Hundreds of annotations were added for each class to ensure robust classifier performance. Particles smaller than 2 µm were excluded from the fibrosis area analysis. Finally, the ratio of the fibrosis area to the total tissue area was calculated and represented.

### SDS-PAGE

Immunoblot analysis was conducted to evaluate the steady-state protein levels in cardiac tissue. For tissue lysates, mice were euthanized via cervical dislocation, followed by opening the chest, excising the hearts, weighing them, flash-freezing them in liquid nitrogen, and storing them at 80 °C. Tissues were prepared by homogenizing them in cold RIPA buffer [1 mg/20 µL, 1% Triton X-100, 1% sodium deoxycholate, 0.1% SDS, 150 mM NaCl, 50 mM Tris·HCl (pH 7.8), 1 mM EDTA, and 1 mM EGTA] with protease and phosphatase inhibitors, and kept on ice for 30 min. The homogenate was then centrifuged for 15 min at 16,000× *g*, 4 °C. Protein concentration was measured by the Bradford assay (Bio-Rad) using a BSA standard curve, with absorbance read at 595 nm using an Infinite M2000 microplate reader (Tecan). Equal amounts of protein were mixed with 4× Laemmli Sample Buffer [355 mM, 2-mercaptoethanol, 62.5 mM Tris-HCl pH 6.8, 10% (v/v) glycerol, 1% (w/v) SDS, 0.005% (v/v) Bromophenol Blue], then heated at 70 °C for 5 min. Samples (10 µg) were separated on 4–20% polyacrylamide gels (Mini Protean TGX Stain-Free gels, Bio-Rad) and transferred to nitrocellulose membranes using the Trans-Blot Turbo Transfer system (Bio-Rad). Consistent protein loading across lanes was verified using Ponceau S staining or Stain-free detection. Membranes were blocked for 2 h with 5% (w/v) semi-skimmed dry milk in Tris-buffered saline with 0.1% Tween (TBST), then incubated overnight at 4 °C with primary antibodies (Dataset EV4) diluted 1:1000 in 2% (w/v) Bovine Serum Albumin (BSA) in 0.1% TBST. The following day, membranes were incubated with HRP-conjugated secondary antibodies at room temperature for 2 h (diluted 1:10,000 in 2% BSA TBST 0.1%). Finally, membranes were treated with Clarity Western ECL Substrate (Bio-Rad) for 2 min, and luminescence was detected using the ChemiDoc Gel Imaging System. Densitometric analysis of the immunoblots was performed using Image Lab Software v.6.1.0 (Bio-Rad).

### Mitochondrial isolation

Freshly isolated cardiac and liver mitochondria were prepared as previously detailed (Donnarumma et al, 2022; Patitucci et al, 2023). In summary, ventricles were separated from atria and non-cardiac tissues, chopped into small pieces, and then manually homogenized in an ice-cold 2-ml homogenizer with IB buffer (275 mM sucrose, 20 mM Tris, 1 mM EGTA-KOH, pH 7.2) containing Trypsin-EDTA (0.05%). To inhibit trypsin activity, bovine serum albumin (BSA) fatty acid-free (0.25 mg/mL), and protease inhibitor cocktail (PIC, Roche) were added. The liver was manually homogenized in an ice-cold 2-ml homogenizer with MIB buffer (275 mM sucrose, 20 mM Tris, 1 mM EGTA-KOH, pH 7.2) containing bovine serum albumin (BSA), fatty acid-free (0.25 mg/mL), and protease inhibitor cocktail (PIC, Roche). The cardiac and liver homogenates were first centrifuged at low speed (1000× *g*, 10 min, 4 °C) to eliminate nuclei and debris, then centrifuged again at a higher speed (3200× *g*, 15 min, 4 °C) to isolate the crude mitochondrial fraction. The crude mitochondrial pellet was resuspended in MIB buffer, and the protein concentration was measured using the Bradford assay.

### High-resolution (Fluo)respirometry

Oxygen consumption and mitochondrial membrane potential were measured in cardiac and liver mitochondria using the High-Resolution Respirometry system (O2k-Fluorespirometer, Oroboros, AT). For this, freshly isolated cardiac and liver mitochondria from adult WT, Chchd10^*S55L*^, *Oma1*^*E324Q/E324Q*^, and Chchd10^*S55L*^*/Oma1*^*E324Q/E324Q*^ mice (7 or 14 weeks of age) were used, as described above. The respiration and membrane potential (Δψ) of the mitochondria were analyzed using O2K-Fluorometry with the O2K-Fluorescence LED2-Module connected via the amperometric channel of the O2K. Briefly, 50 µg of cardiac or liver mitochondria were suspended in Mir05 buffer [MgCl_2_-6H_2_O 3 mM, Lactobionic Acid 60 mM, Taurine 20 mM, KH_2_PO_4_ 10 mM, Hepes-KOH 20 mM, Sucrose 110 mM, EGTA-KOH 0.5 mM, BSA (1 g/L)]. Rhodamine 123 (RH-123) (0.66 µM) fluorescence quenching (Δ fluorescence) was used to measure the membrane potential (Δψ) in energized mitochondria. Maximal mitochondrial respiration capacity (OXPHOS) was determined by adding PGM [10 mM pyruvate, 5 mM glutamate, 5 mM malate, state 2] or malate (2 mM) and palmitoyl-carnitine (10 µM) in the presence of ADP (1 mM, state 3) to measure complex I-driven respiration; rotenone (0.5 µM) and succinate (10 mM, state 2) to measure complex II-driven respiration; and antimycin A (2.5 µM), ascorbate (2 mM), *N,N,N’,N’-tetramethyl-p-phenylenediamine* (TMPD, 0.5 mM), and carbonyl cyanide m-chlorophenyl hydrazone (CCCP, 2 µM) to measure complex IV-driven respiration. Oxygen consumption was assessed using 50 µg of mitoplasts— cardiac mitochondria subjected to a freeze–thaw cycle—resuspended in Mir05 buffer, following the sequential addition of antimycin A (2.5 µM), ascorbate (2 mM), *N,N,N’,N’-tetramethyl-p-phenylenediamine* (TMPD, 0.5 mM), and bovine cytochrome c (10 µM). The results were analyzed using Datlab v.7.

### Alkaline carbonate extraction

Alkaline carbonate extraction of membrane proteins was carried out as previously reported (Anand et al, 2014). Crude mitochondria, isolated from mouse hearts, were resuspended and treated with 0.1 M sodium carbonate (Na₂CO₃) at pH levels of 12.5 or 9.5, and incubated on ice for 30 min. The mixtures were then ultracentrifuged at 45,000 rpm for 30 min at 4 °C using Beckman polycarbonate tubes and a TLA 55 rotor. Both the supernatants and pellets were treated with a trichloroacetic acid buffer (17.5% TCA, 40 mM HEPES, 0.02% Triton X-100) on ice for 30 min, followed by centrifugation at 21,130× *g* for 20 min at 4 °C. The resulting samples were washed three times with cold 100% acetone and left to air-dry at room temperature for 30 min. Finally, the dried pellets were resuspended in 1× Laemmli sample buffer (Bio-Rad) for analysis via SDS-PAGE and western blot.

### Differential solubility proteomics

To obtain soluble and insoluble mitochondrial protein fractions, 150 µg of mitochondria were pelleted at 20,000× *g* for 5 min at 4 °C in two separate tubes. One pellet was resuspended in 45 µL 2× Laemmli buffer (total fraction). The second pellet was resuspended in 37.5 µL Fractionation Buffer A (10 mM TRIS-HCl, pH=8; 1 mM EDTA; 1% Triton X-100 (1% v/v); cOmplete protease inhibitor (Roche) and incubated at 4 °C for 10 min. After centrifugation at 20,000× *g* for 10 min at 4 °C. 37.5 µL of the supernatant was transferred to a fresh tube (soluble fraction) and supplemented with 7.5 µL 4× Laemmli buffer. The insoluble pellet was washed twice with 150 µL Fractionation Buffer A and subsequently centrifuged at 20,000× *g* for 10 min at 4 °C. The supernatant was discarded, and the pellet was resuspended in 45 µL Urea sample buffer (125 mM TRIS-HCl, pH=6.8; 6 M Urea, 6%SDS; 0.5% beta-mercaptoethanol; 0.01% bromphenol blue). The individual fractions, total (mitochondria), pellet, and supernatant, were subjected for protein digestion and LC-MS/MS measurement.

### Protein digestion

One-third of each fraction (total, pellet, supernatant) was subjected for trypsin digestion. In detail, proteins were reduced (10 mM TCEP) and alkylated (20 mM CAA) in the dark for 45 min at 45 °C. Samples were subjected to an SP3-based digestion (Hughes et al, 2014). Washed SP3 beads (SP3 beads (Sera-Mag(TM) Magnetic Carboxylate Modified Particles (Hydrophobic, GE44152105050250), Sera-Mag(TM) Magnetic Carboxylate Modified Particles (Hydrophilic, GE24152105050250) from Sigma-Aldrich) were mixed equally, and 3 µL of bead slurry were added to each sample. Acetonitrile was added to a final concentration of 50% and washed twice using 70% ethanol (V = 200 µL) on an in-house manufactured magnet. After an additional acetonitrile wash (V = 200 µL), 5 µL digestion solution (10 mM HEPES pH = 8.5 containing 0.5 µg Trypsin (Sigma) and 0.5 µg LysC (Wako)) was added to each sample and incubated overnight at 37 °C. Peptides were desalted on a magnet using 2 ×200 µL acetonitrile. Peptides were eluted in 10 µL 5% DMSO in LC-MS water (Sigma-Aldrich) in an ultrasonic bath for 10 min. Eluted tryptic peptides were subjected to further peptide purification using the StageTip technique using the SDB material. Samples were stored at −20 °C, and 10 µL of 2.5% formic acid and 2% acetonitrile were added. In total, 3 µL were used for a LC-MS/MS run.

### Liquid chromatography and mass spectrometry

LC-MS/MS instrumentation consisted of an Easy-LC 1200 (Thermo Fisher Scientific) coupled via a nano-electrospray ionization source to an Exploris 480 mass spectrometer (Thermo Fisher Scientific, Bremen, Germany). An Aurora Frontier column (60 cm length, 1.7 µm particle diameter, 75 µm inner diameter, Ionopticks). A binary buffer system (A: 0.1% formic acid and B: 0.1% formic acid in 80% acetonitrile) based gradient was utilized as follows at a flow rate of 185 nL/min; a linear increase of buffer B from 4% to 28% within 100 min, followed by a linear increase to 40% within 10 min. The buffer B content was further ramped to 50% within 4 min and then to 65% within 3 min. 95% buffer B was kept for a further 3 min to wash the column. The RF Lens amplitude was set to 45%, the capillary temperature was 275 °C, and the polarity was set to positive. MS1 profile spectra were acquired using a resolution of 30,000 (at 200 *m/z*) at a mass range of 450–850 *m/z* and an AGC target of 1 × 10^6^. For MS/MS independent spectra acquisition, 34 equally spaced windows were acquired at an isolation *m/z* range of 7 Th, and the isolation windows overlapped by 1 Th. The fixed first mass was 200 *m/z*. The isolation center range covered a mass range of 500–740 *m/z*. Fragmentation spectra were acquired at a resolution of 30,000 at 200 *m/z* using a maximal injection time setting of “auto” and stepped normalized collision energies (NCE) of 24, 28, and 30. The default charge state was set to 3. The AGC target was set to 3e6 (900% - Exploris 480). MS2 spectra were acquired in centroid mode. FAIMS was enabled using an inner electrode temperature of 100 °C and an outer electrode temperature of 90 °C. The compensation voltage was set to −45 V.

Raw files were analyzed using Spectronaut 19.3.241023.62635 in direct DIA mode using the Uniprot Mus musculus (Mouse) UP000005640 proteome—one fasta sequence per gene, reviewed, 21,984 protein sequences. Trypsin/P was selected as the cleavage rule using a specific digest type. The minimal peptide length was set to seven, and a total of two missed cleavages was allowed. The peptide spectrum match (PSM), peptide, and protein group FDR were controlled to 0.01. The mass tolerances were used with default settings (Dynamic, 1). The directDIA + (deep) workflow was selected, and cross-run normalization (only for the whole proteome analysis) was enabled.

For whole-proteome analysis (total fraction), the protein group file was exported, and LFQ intensities (MaxLFQ algorithm) (Cox et al, 2014) were log2-transformed. Statistically significantly different proteins were identified using a two-sided *t* test followed by a permutation-based FDR calculation (s0 = 0.1, number permutations=500, FDR < 0.05) using Instant Clue (Nolte et al, 2018).

ISR genes were annotated according to Class 1, and ATF-dependent upregulated genes were defined previously (Labbé et al, 2024).

For solubility proteome analysis, the iBAQ (intensity-based absolute quantification) intensity was used. The individual fractions (total, pellet, supernatant) were median normalized within the groups. Then the pellet fraction was calculated as iBAQ total/iBAQ pellet.

### Inductively coupled plasma optical emission spectroscopy

Heart tissue samples (9–30 mg) and mitochondrial preparations (equivalent to 100 µg protein) were analyzed for elemental content using inductively coupled plasma–optical emission spectrometry (ICP-OES; Optima 7300DV, PerkinElmer Life Sciences). Samples were digested in 40% nitric acid by boiling for 1 h in acid-washed, semi-sealed tubes. After digestion, samples were diluted with ultrapure, metal-free water (18.2 MΩ·cm) prior to analysis. Emission intensities for Ca, Cu, Fe, K, Mg, Mn, P, S, and Zn were measured simultaneously, with acid matrix blanks prepared identically to the samples for background correction. Each biological replicate (*n* = 5–6 per genotype and sex) was measured in technical triplicate, and the average intensity calculated by area under the curve was used for quantification. Elemental concentrations were calculated using three-point standard curves generated from serial dilutions of two commercially available mixed-metal standards (Optima). Blanks of the nitric acid matrix, with and without metal spikes, were included to confirm the reproducibility and accuracy of measurements.

### Heme quantification by HPLC

Mitochondria equivalent to 100 µg of protein were used for heme analysis by HPLC. Heme was extracted as previously described (Pierrel et al, 2007). Mitochondrial pellets were resuspended in 100 µL of 97.5% acetone/2.5% hydrochloric acid (v/v), vortexed for 1 min, and centrifuged at 12,000×*g* for 5 min at room temperature. The supernatant was carefully transferred to a new tube to avoid disturbing the pellet. This acid-acetone extract was mixed with 100 µL acetonitrile, 1 µL formic acid, and 7 µL ammonium hydroxide, then vortexed briefly and centrifuged again at 12,000×*g* for 2 min. An aliquot of 100 µL of the final solution was injected onto a Sonoma C18 reversed-phase HPLC column (C18(2) 10 µ 100 Å 25 cm × 4.6 mm), equilibrated in 0.05% trifluoroacetic acid with 30% acetonitrile. Chromatographic separation was achieved using a 5-min linear gradient from 30% to 50% acetonitrile, followed by a 20-min gradient from 50% to 75%, at a flow rate of 1 mL/min. The column was subsequently washed with 99% acetonitrile 0.05% trifluoroacetic acid then re-equilibrated with the starting buffer. Heme elution was monitored at 405 nm via the Soret band. Retention times were determined using purified standards of heme b, heme o, and heme a. Quantification was based on the area under the curve, with only heme b concentrations reported.

### Cardiac RT-qPCR and bulk RNAseq

Total RNA was isolated from snap-frozen left ventricles by the NucleoSpin RNA kit (Macherey-Nagel, 740955). Quality control was performed on an Agilent BioAnalyzer. Libraries were built using a TruSeq Stranded mRNA library Preparation Kit (Illumina, USA) following the manufacturer’s protocol. For the Control (*Yme1l1*^*LoxP/LoxP*^) and Y*me1l1*^*Heart*^ mice samples, two runs of RNA sequencing were performed for each library on an Illumina NextSeq 500 platform using single-end 75 bp. For the *Chchd10*^*S55L/+*^ and *Oma1*^*E324Q/E324Q*^ mutant mice, a single run was performed for all libraries on an Illumina NextSeq 2000 platform using paired-end 52b reads. The RNA-seq analysis was performed with Sequana 0.18.1. In particular, we used the RNA-seq pipeline (version 0.20.0) (https://github.com/sequana/sequana_rnaseq) built on top of Snakemake 7.32.4 (Köster and Rahmann, 2012). Reads were trimmed from adapters using Fastp 0.23.2, then mapped to the mouse reference genome GRCm39 using STAR 2.7.10a (Dobin et al, 2013). FeatureCounts 2.0.1 was used to produce the count matrix, assigning reads to features using annotation from Ensembl GRCm39_113 with strand-specificity information (Liao et al, 2014). Quality control statistics were summarized using MultiQC 1.16.0 (Ewels et al, 2016). Clustering of transcriptomic profiles was assessed using a Principal Component Analysis (PCA). Differential expression testing was conducted using DESeq2 library 1.38.3.0 (Love et al, 2014) scripts, indicating the significance (Benjamini-Hochberg adjusted *P* values, false discovery rate FDR < 0.05) and the effect size (fold change) for each comparison. Over-representation analysis (ORA) was performed to determine if genes modulated by genotype or biological sex are more present in specific pathways. ORA was performed on WebGestalt (https://www.webgestalt.org/). RNAseq data have been deposited at ENA with the dataset identifiers E-MTAB-15304 and E-MTAB-15305. For RT-qPCR, 1 µg of total RNA was converted into cDNA using the iScript Reverse Transcription Supermix (Bio-Rad). RT-qPCR was performed using the CFX384 Touch Real-Time PCR Detection System (Bio-Rad) and SYBR® Green Master Mix (Bio-Rad) using the primers listed in the Reagents and Tools Table. *Gapdh* was amplified as an internal standard. Data were analyzed according to the 2 − ΔΔCT method (Livak and Schmittgen, 2001a).

### mtDNA quantification

Genomic DNA was isolated using the NucleoSpin Tissue kit (MACHEREY-NAGEL) and quantified with a NanoQuant Plate (Infinite M200, TECAN). Quantitative PCR (qPCR) was carried out using the Real-Time PCR Detection System (Applied Biosystems StepOnePlus), with 20 ng of total DNA and SYBR Green Master Mix (Bio-Rad). β-*Actin* was amplified as an internal nuclear gene control, as previously described (Nargund et al, 2012), using primers listed in the “Reagents and Tools Table”. Data were analyzed according to the 2^ − ΔΔCT method (Livak and Schmittgen, 2001b).

### Transmission electron microscopy

Transmission electron microscopy was conducted on cardiac tissue from mice at 14 weeks of age, as previously described(Donnarumma et al, 2022). Small tissue samples (1 × 1 ×  1 mm) from the posterior wall of the left ventricle were fixed initially in a 37 °C pre-warmed solution containing 1× PHEM buffer (60 mM PIPES, 25 mM HEPES, 10 mM EGTA, 2 mM MgCl₂, pH 7.3), 2.5% glutaraldehyde, and 2% paraformaldehyde (PFA) for 30 min, followed by overnight fixation at 4 °C. The specimens were rinsed three times with 3x PHEM buffer, and then were processed following an adapted OTO protocol (Seligman et al, 1966). First, samples were incubated in a mixture of 1% osmium tetroxide (OsO₄) and 1.5% potassium ferrocyanide (K₄Fe(CN)₆) to enhance membrane contrast. Then, samples were treated with 1% tannic acid to further increase contrast. A second post-fixation step was performed with 1% OsO₄ alone. Samples were dehydrated through a graded ethanol series and embedded in SPURR resin (Electron Microscopy Sciences) at 60 °C for 48 h. Thin sections (70 nm) were then cut with a Leica UCT microtome and collected on carbon and formvar-coated copper grids. The sections were contrasted with 4% aqueous uranyl acetate and Reynold’s lead citrate. The images were acquired using a TECNAI T12 Transmission Electron Microscope (FEI), operated at 120 kV with a RIO16 camera (Gatan), controlled by Digital Micrograph software. Mitochondrial ultrastructure quantification was performed using NIH ImageJ software, as previously described (Lam et al, 2021). Mitochondrial area and number were quantified from electron micrographs, with 315–440 mitochondria analyzed per experimental group. The same set of mitochondria was used to assess cristae morphology using a standardized scoring system, as described previously (Eisner et al, 2017).

### 1D BN-PAGE of cardiac mitochondria

One-dimensional blue native PAGE (1D BN-PAGE) was performed following a previously described (Wittig et al, 2006) with some modifications. In summary, heart mitochondria (50 μg, with protein concentration determined using the DC Protein Assay from BIO-RAD) were isolated from WT, *Chchd10*^*S55L*^, *Oma1*^*E324Q/E324Q*^, *Chchd10*^*S55L*^*Oma1*^*E324Q/E324Q*^ mice. These mitochondria were incubated with a digitonin extraction buffer composed of HEPES (30 mM), potassium acetate (150 mM), glycerol (12%), 6-aminocaproic acid (2 mM), EDTA (1 mM), and high-purity digitonin (10%), with a pH of 7.2. The mitochondria were vortexed for 1 h at 4 °C to solubilize the membranes, followed by centrifugation at 21,130× *g* for 30 min. The supernatant was collected and mixed with loading dye containing Coomassie Brilliant Blue G-250 (0.0125% w/v, InvitrogenTM, BN2004). The solubilized mitochondria were loaded onto a 4–16% Bis-Tris acrylamide gel (1 mm, InvitrogenTM NovexTM NativePAGETM, BN2111BX10), using an anode buffer (Invitrogen, BN2001) with Cathode Buffer Additive (0.5%) mixed into the anode buffer (Invitrogen, BN2002). Electrophoresis was carried out at 80 V and 20 mA for 45 min, then at 150 V and 20 mA for 13 h. Afterward, the gel was incubated in transfer buffer (0.304% w/v Tris, 1.44% w/v glycine) containing 0.2% SDS and 0.2% β-mercaptoethanol for 30 min at room temperature to denature the proteins. Following this, proteins were transferred to a polyvinylidene difluoride (PVDF) membrane in transfer buffer (0.304% w/v Tris, 1.44% w/v glycine, and 10% v/v ethanol) at 400 mA and 20 V for 3 h and 30 min. The membrane was washed with methanol to remove the Coomassie stain. For immunodetection, the membrane was blocked with 5% milk in TBST (Tween-Tris-buffered saline) for 2 h at room temperature, then incubated overnight with a specific primary antibody diluted in the blocking solution. The next day, the membrane was washed three times in TBST, then incubated with an HRP-conjugated secondary antibody for 2 h at room temperature. Finally, the membrane was treated with Tris-HCl (0.1 M, pH 8.5) containing luminol and p-coumaric acid for 3 min, and luminescence was detected using a ChemiDoc TM XRS+ Imaging System. Band intensities were quantified using Image Lab Software.

### Cell lines

WT (*Chchd10*^*+/+*^) and mutant (*Chchd10*^*S55L/+*^) embryos were isolated at E13.5 following F1 heterozygous intercrosses of Wt and *Chchd10*^*S55L/+*^ mice. Immortalization of primary mouse embryonic fibroblasts (MEFs) was performed as previously described (Cretin et al, 2021) using a plasmid encoding SV40 large T antigen. MEFs cells were maintained in Dulbecco’s modified Eagle’s medium (DMEM + GlutaMAX, 4.5 g/L D-Glucose, pyruvate) supplemented with 5% FBS and 1% penicillin/streptomycin (P/S, 50 µg/ml) in a 5% CO_2_ atmosphere at 37 °C. MEFs were plated in Cell Carrier Ultra-96 well (PerkinElmer) and incubated for 24 h with media containing glucose (4.5 g/L) or galactose (4.5 g/L). Cells were then stained with NucBlue™ Live ReadyProbes™ to visualize nuclei. Propidium Iodide (PI, 1:500) was added to visualize dead cells. Cell death was induced with actinomycin D (1.5 µM) in association with ABT-737 (10 µM), an inhibitor of the Bcl-2 family proteins, or staurosporine (1 µM) or etoposide (500 µM).

Image acquisition was performed every hour using the Operetta CLS High-Content Analysis system (PerkinElmer) with a ×20 air objective. CE or PI-positive nuclei number were automatically counted by using Harmony v.4.9 software.

### Determination of serum levels of cardiac troponin I

A mouse ELISA kit was used to compare serum levels of cardiac troponin I (cTnI, Life Diagnostics). Blood was collected via submandibular vein puncture from non-anesthetized mice, left for 30 min at RT, and then centrifuged at 5000× *g* at 4 °C for 10 min, then snap-frozen in liquid nitrogen. Serum was stored at −80 °C until next use. The assays to determine cTnI levels were performed following the exact manufacturer’s instructions.

### Statistical analyses

Experiments were conducted at least three times, with quantitative analyses performed in a blinded manner. Group randomization (e.g., by genotype) was applied when simultaneous, parallel measurements were not feasible (e.g., Oroboros, cardiac isolation). For high-throughput assessments (e.g., proteomics, immunoblots, qRT-PCR), all groups were measured in parallel to minimize experimental bias. Statistical analyses were conducted using GraphPad Prism v10 software, and data are presented as mean ± SD or SEM, as specified. Statistical tests and replicate numbers are detailed in the figure legends. Comparisons between two groups were made using an unpaired two-tailed T-test, while one-way or two-way ANOVA was used to compare more than two groups or groups across multiple time points. Significance was set at *P* < 0.05, with levels denoted as *P < 0.05, **P < 0.01, ***P < 0.001, and ****P < 0.0001.

### Graphics

Figures 1A, 3A, 3B, 4D, and EV4B, and synopsis graphics were created with Biorender.com.

## Supplementary information

Appendix

Peer Review File

Dataset EV1

Dataset EV2

Dataset EV3

Dataset EV4

Dataset EV5

Dataset EV6

Dataset EV7

Source data Fig. 1

Source data Fig. 2

Source data Fig. 3

Source data Fig. 4

Figure EV1 Source Data

Figure EV2 Source Data

Figure EV3 Source Data

Expanded View Figures

## Data Availability

All source data for the experiments are available with this manuscript. The datasets generated in this study have been deposited in the Proteomics Identification Database (PRIDE). Accession numbers are as follows: Differential solubility proteomics and proteome (PRIDE identifier: PXD064057 and PXD064045) and transcriptomics (ENA E-MTAB-15304 and E-MTAB-15305). The processed and analyzed data for proteomics are included as Supplementary Datasets. Complete datasets for echocardiographic measurements analyzed in this study are not publicly available due to the limitations in exporting understandable file names from Vevo 3100 software (Visualsonics). However, these datasets are available upon request. Source data are included with this paper. The source data of this paper are collected in the following database record: biostudies:S-SCDT-10_1038-S44321-025-00358-5.

## References

[CR1] Ahola S, Rivera Mejías P, Hermans S, Chandragiri S, Giavalisco P, Nolte H, Langer T (2022) OMA1-mediated integrated stress response protects against ferroptosis in mitochondrial cardiomyopathy. Cell Metab 34:1875–1891 36113464 10.1016/j.cmet.2022.08.017

[CR2] Al-Habib H, Ashcroft M (2021) CHCHD4 (MIA40) and the mitochondrial disulfide relay system. Biochem Soc Trans 49:17–27 33599699 10.1042/BST20190232 PMC7925007

[CR3] Alici H, Uversky VN, Kang DE, Woo JA, Coskuner-Weber O (2022) Structures of the wild-type and S59L mutant CHCHD10 proteins important in amyotrophic lateral sclerosis-frontotemporal dementia. ACS Chem Neurosci 13:1273–1280 35349255 10.1021/acschemneuro.2c00011

[CR4] Anand R, Reichert AS, Kondadi AK (2021) Emerging roles of the MICOS complex in cristae dynamics and biogenesis. Biology 10:600 10.3390/biology10070600 PMC8301002 34209580

[CR5] Anand R, Wai T, Baker MJ, Kladt N, Schauss AC, Rugarli E, Langer T (2014) The i-AAA protease YME1L and OMA1 cleave OPA1 to balance mitochondrial fusion and fission. J Cell Biol 204:919–929 24616225 10.1083/jcb.201308006 PMC3998800

[CR6] Anderson CJ, Bredvik K, Burstein SR, Davis C, Meadows SM, Dash J, Case L, Milner TA, Kawamata H, Zuberi A et al (2019) ALS/FTD mutant CHCHD10 mice reveal a tissue-specific toxic gain-of-function and mitochondrial stress response. Acta Neuropathol 138:103–121 30877432 10.1007/s00401-019-01989-y PMC6571048

[CR7] Antonicka H, Leary SC, Guercin G-H, Agar JN, Horvath R, Kennaway NG, Harding CO, Jaksch M, Shoubridge EA (2003a) Mutations in COX10 result in a defect in mitochondrial heme A biosynthesis and account for multiple, early-onset clinical phenotypes associated with isolated COX deficiency. Hum Mol Genet 12:2693–2702 12928484 10.1093/hmg/ddg284

[CR8] Antonicka H, Mattman A, Carlson CG, Glerum DM, Hoffbuhr KC, Leary SC, Kennaway NG, Shoubridge EA (2003b) Mutations in COX15 produce a defect in the mitochondrial heme biosynthetic pathway, causing early-onset fatal hypertrophic cardiomyopathy. Am J Hum Genet 72:101–114 12474143 10.1086/345489 PMC378614

[CR9] Auranen M, Ylikallio E, Shcherbii M, Paetau A, Kiuru-Enari S, Toppila JP, Tyynismaa H (2015) CHCHD10 variant p.(Gly66Val) causes axonal Charcot-Marie-Tooth disease. Neurol Genet 1:e1 27066538 10.1212/NXG.0000000000000003 PMC4821082

[CR10] Babbitt SE, Sutherland MC, San Francisco B, Mendez DL, Kranz RG (2015) Mitochondrial cytochrome c biogenesis: no longer an enigma. Trends Biochem Sci 40:446–455 26073510 10.1016/j.tibs.2015.05.006 PMC4509832

[CR11] Baek M, Choe Y-J, Bannwarth S, Kim J, Maitra S, Dorn 2ndGW, Taylor JP, Paquis-Flucklinger V, Kim NC (2021) TDP-43 and PINK1 mediate CHCHD10S59L mutation-induced defects in Drosophila and in vitro. Nat Commun 12:1924 33772006 10.1038/s41467-021-22145-9 PMC7997989

[CR12] Baker MJ, Lampe PA, Stojanovski D, Korwitz A, Anand R, Tatsuta T, Langer T (2014) Stress-induced OMA1 activation and autocatalytic turnover regulate OPA1-dependent mitochondrial dynamics. EMBO J 33:578–593 24550258 10.1002/embj.201386474 PMC3989652

[CR13] Baker MJ, Mooga VP, Guiard B, Langer T, Ryan MT, Stojanovski D (2012) Impaired folding of the mitochondrial small TIM chaperones induces clearance by the i-AAA protease. J Mol Biol 424:227–239 23036860 10.1016/j.jmb.2012.09.019

[CR14] Baker ZN, Jett K, Boulet A, Hossain A, Cobine PA, Kim B-E, El Zawily AM, Lee L, Tibbits GF, Petris MJ et al (2017) The mitochondrial metallochaperone SCO1 maintains CTR1 at the plasma membrane to preserve copper homeostasis in the murine heart. Hum Mol Genet 26:4617–4628 28973536 10.1093/hmg/ddx344 PMC5886179

[CR15] Bankhead P, Loughrey MB, Fernández JA, Dombrowski Y, McArt DG, Dunne PD, McQuaid S, Gray RT, Murray LJ, Coleman HG et al (2017) QuPath: open source software for digital pathology image analysis. Sci Rep 7:16878 29203879 10.1038/s41598-017-17204-5 PMC5715110

[CR16] Bannwarth S, Ait-El-Mkadem S, Chaussenot A, Genin EC, Lacas-Gervais S, Fragaki K, Berg-Alonso L, Kageyama Y, Serre V, Moore DG et al (2014) A mitochondrial origin for frontotemporal dementia and amyotrophic lateral sclerosis through CHCHD10 involvement. Brain 137:2329–2345 24934289 10.1093/brain/awu138 PMC4107737

[CR17] Besse A, Wu P, Bruni F, Donti T, Graham BH, Craigen WJ, McFarland R, Moretti P, Lalani S, Scott KL et al (2015) The GABA transaminase, ABAT, is essential for mitochondrial nucleoside metabolism. Cell Metab 21:417–427 25738457 10.1016/j.cmet.2015.02.008 PMC4757431

[CR18] Bonekamp NA, Peter B, Hillen HS, Felser A, Bergbrede T, Choidas A, Horn M, Unger A, Di Lucrezia R, Atanassov I et al (2020) Small-molecule inhibitors of human mitochondrial DNA transcription. Nature 588:712–716 33328633 10.1038/s41586-020-03048-z

[CR19] Boulet A, Vest KE, Maynard MK, Gammon MG, Russell AC, Mathews AT, Cole SE, Zhu X, Phillips CB, Kwong JQ et al (2018) The mammalian phosphate carrier SLC25A3 is a mitochondrial copper transporter required for cytochrome c oxidase biogenesis. J Biol Chem 293:1887–1896 29237729 10.1074/jbc.RA117.000265 PMC5808751

[CR20] Brownstein DG (2003) Manipulating the mouse embryo: a laboratory manual. Q Rev Biol 78:365–365

[CR21] Carrozzo R, Dionisi-Vici C, Steuerwald U, Lucioli S, Deodato F, Di Giandomenico S, Bertini E, Franke B, Kluijtmans LAJ, Meschini MC et al (2007) SUCLA2 mutations are associated with mild methylmalonic aciduria, Leigh-like encephalomyopathy, dystonia and deafness. Brain 130:862–874 17301081 10.1093/brain/awl389

[CR22] Cheng M, Lu D, Li K, Wang Y, Tong X, Qi X, Yan C, Ji K, Wang J, Wang W et al (2025) Mitochondrial respiratory complex IV deficiency recapitulates amyotrophic lateral sclerosis. Nat Neurosci 28:748–756 40069360 10.1038/s41593-025-01896-4

[CR23] Chojnacka M, Gornicka A, Oeljeklaus S, Warscheid B, Chacinska A (2015) Cox17 protein is an auxiliary factor involved in the control of the mitochondrial contact site and cristae organizing system. J Biol Chem 290:15304–15312 25918166 10.1074/jbc.M115.645069 PMC4463469

[CR24] Chung KW, Dhillon P, Huang S, Sheng X, Shrestha R, Qiu C, Kaufman BA, Park J, Pei L, Baur J et al (2019) Mitochondrial damage and activation of the STING pathway lead to renal inflammation and fibrosis. Cell Metab 30:784–799.e5 31474566 10.1016/j.cmet.2019.08.003 PMC7054893

[CR25] Close A-F, Chae H, Jonas J-C (2021) The lack of functional nicotinamide nucleotide transhydrogenase only moderately contributes to the impairment of glucose tolerance and glucose-stimulated insulin secretion in C57BL/6 J vs C57BL/6 N mice. Diabetologia 64:2550–2561 34448880 10.1007/s00125-021-05548-7

[CR26] Cobine PA, Moore SA, Leary SC (2021) Getting out what you put in: copper in mitochondria and its impacts on human disease. Biochim Biophys Acta Mol Cell Res 1868:118867 32979421 10.1016/j.bbamcr.2020.118867 PMC7680424

[CR27] Cogliati S, Calvo E, Loureiro M, Guaras AM, Nieto-Arellano R, Garcia-Poyatos C, Ezkurdia I, Mercader N, Vázquez J, Enriquez JA (2016) Mechanism of super-assembly of respiratory complexes III and IV. Nature 539:579–582 27775717 10.1038/nature20157

[CR28] Cox J, Hein MY, Luber CA, Paron I, Nagaraj N, Mann M (2014) Accurate proteome-wide label-free quantification by delayed normalization and maximal peptide ratio extraction, termed MaxLFQ. Mol Cell Proteom 13:2513–2526 10.1074/mcp.M113.031591 PMC4159666 24942700

[CR29] Cretin E, Lopes P, Vimont E, Tatsuta T, Langer T, Gazi A, Sachse M, Yu-Wai-Man P, Reynier P, Wai T (2021) High-throughput screening identifies suppressors of mitochondrial fragmentation in OPA1 fibroblasts. EMBO Mol Med 13:e13579 34014035 10.15252/emmm.202013579 PMC8185549

[CR30] Croon M, Szczepanowska K, Popovic M, Lienkamp C, Senft K, Brandscheid CP, Bock T, Gnatzy-Feik L, Ashurov A, Acton RJ et al (2022) FGF21 modulates mitochondrial stress response in cardiomyocytes only under mild mitochondrial dysfunction. Sci Adv 8:eabn7105 35385313 10.1126/sciadv.abn7105 PMC8986112

[CR31] Cubadda F (2007) Inductively coupled plasma mass spectrometry. In: Picó Y (ed) Food toxicants analysis. Elsevier, pp 697–751

[CR32] Daumke O, van der Laan M (2025) Molecular machineries shaping the mitochondrial inner membrane. Nat Rev Mol Cell Biol 26:706–724 10.1038/s41580-025-00854-z 40369159

[CR33] Di Fonzo A, Ronchi D, Lodi T, Fassone E, Tigano M, Lamperti C, Corti S, Bordoni A, Fortunato F, Nizzardo M et al (2009) The mitochondrial disulfide relay system protein GFER is mutated in autosomal-recessive myopathy with cataract and combined respiratory-chain deficiency. Am J Hum Genet 84:594–604 19409522 10.1016/j.ajhg.2009.04.004 PMC2681006

[CR34] Dickson-Murray E, Nedara K, Modjtahedi N, Tokatlidis K (2021) The Mia40/CHCHD4 oxidative folding system: redox regulation and signaling in the mitochondrial intermembrane space. Antioxidants 10:592 10.3390/antiox10040592 PMC8069373 33921425

[CR35] Dobin A, Davis CA, Schlesinger F, Drenkow J, Zaleski C, Jha S, Batut P, Chaisson M, Gingeras TR (2013) STAR: ultrafast universal RNA-seq aligner. Bioinformatics 29:15–21 23104886 10.1093/bioinformatics/bts635 PMC3530905

[CR36] Donnarumma E, Kohlhaas M, Vimont E, Kornobis E, Chaze T, Gianetto QG, Matondo M, Moya-Nilges M, Maack C, Wai T (2022) Mitochondrial fission process 1 controls inner membrane integrity and protects against heart failure. Nat Commun 13:6634 36333300 10.1038/s41467-022-34316-3 PMC9636241

[CR37] Eisner V, Cupo RR, Gao E, Csordás G, Slovinsky WS, Paillard M, Cheng L, Ibetti J, Chen SRW, Chuprun JK et al (2017) Mitochondrial fusion dynamics is robust in the heart and depends on calcium oscillations and contractile activity. Proc Natl Acad Sci USA 114:E859–E868 28096338 10.1073/pnas.1617288114 PMC5293028

[CR38] Ewels P, Magnusson M, Lundin S, Käller M (2016) MultiQC: summarize analysis results for multiple tools and samples in a single report. Bioinformatics 32:3047–3048 27312411 10.1093/bioinformatics/btw354 PMC5039924

[CR39] Fessler E, Eckl E-M, Schmitt S, Mancilla IA, Meyer-Bender MF, Hanf M, Philippou-Massier J, Krebs S, Zischka H, Jae LT (2020) A pathway coordinated by DELE1 relays mitochondrial stress to the cytosol. Nature 579:433–437 32132706 10.1038/s41586-020-2076-4 PMC7116715

[CR40] Fessler E, Krumwiede L, Jae LT (2022) DELE1 tracks perturbed protein import and processing in human mitochondria. Nat Commun 13:1–15 35388015 10.1038/s41467-022-29479-y PMC8986780

[CR41] Formosa LE, Maghool S, Sharpe AJ, Reljic B, Muellner-Wong L, Stroud DA, Ryan MT, Maher MJ (2022) Mitochondrial COA7 is a heme-binding protein with disulfide reductase activity, which acts in the early stages of complex IV assembly. Proc Natl Acad Sci USA 119:e2110357119 35210360 10.1073/pnas.2110357119 PMC8892353

[CR42] Franco A, Kitsis RN, Fleischer JA, Gavathiotis E, Kornfeld OS, Gong G, Biris N, Benz A, Qvit N, Donnelly SK et al (2016) Correcting mitochondrial fusion by manipulating mitofusin conformations. Nature 540:74–79 27775718 10.1038/nature20156 PMC5315023

[CR43] Franklin CL, Ericsson AC (2017) Microbiota and reproducibility of rodent models. Lab Anim 46:114–122 10.1038/laban.1222 PMC5762113 28328896

[CR44] Frezza C, Cipolat S, Martins de Brito O, Micaroni M, Beznoussenko GV, Rudka T, Bartoli D, Polishuck RS, Danial NN, De Strooper B et al (2006) OPA1 controls apoptotic cristae remodeling independently from mitochondrial fusion. Cell 126:177–189 16839885 10.1016/j.cell.2006.06.025

[CR45] Garrido C, Galluzzi L, Brunet M, Puig PE, Didelot C, Kroemer G (2006) Mechanisms of cytochrome c release from mitochondria. Cell Death Differ 13:1423–1433 16676004 10.1038/sj.cdd.4401950

[CR46] Genin EC, Bannwarth S, Lespinasse F, Ortega-Vila B, Fragaki K, Itoh K, Villa E, Lacas-Gervais S, Jokela M, Auranen M et al (2018) Loss of MICOS complex integrity and mitochondrial damage, but not TDP-43 mitochondrial localisation, are likely associated with severity of CHCHD10-related diseases. Neurobiol Dis 119:159–171 30092269 10.1016/j.nbd.2018.07.027 PMC7015038

[CR47] Genin EC, Bannwarth S, Ropert B, Lespinasse F, Mauri-Crouzet A, Augé G, Fragaki K, Cochaud C, Donnarumma E, Lacas-Gervais S et al (2022) CHCHD10 and SLP2 control the stability of the PHB complex: a key factor for motor neuron viability. Brain 145:3415–3430 35656794 10.1093/brain/awac197

[CR48] Genin EC, di Borgo PP, Lorivel T, Hugues S, Farinelli M, Mauri-Crouzet A, Lespinasse F, Godin L, Paquis-Flucklinger V, Petit-Paitel A (2024) CHCHD10S59L/+ mouse model: behavioral and neuropathological features of frontotemporal dementia. Neurobiol Dis 195:106498 38583639 10.1016/j.nbd.2024.106498

[CR49] Genin EC, Madji Hounoum B, Bannwarth S, Fragaki K, Lacas-Gervais S, Mauri-Crouzet A, Lespinasse F, Neveu J, Ropert B, Augé G et al (2019) Mitochondrial defect in muscle precedes neuromuscular junction degeneration and motor neuron death in CHCHD10S59L/+ mouse. Acta Neuropathol 138:123–145 30874923 10.1007/s00401-019-01988-z

[CR50] Genin EC, Plutino M, Bannwarth S, Villa E, Cisneros-Barroso E, Roy M, Ortega-Vila B, Fragaki K, Lespinasse F, Pinero-Martos E et al (2016) CHCHD10 mutations promote loss of mitochondrial cristae junctions with impaired mitochondrial genome maintenance and inhibition of apoptosis. EMBO Mol Med 8:58–72 26666268 10.15252/emmm.201505496 PMC4718158

[CR51] Goetze JP, Bruneau BG, Ramos HR, Ogawa T, de Bold MK, de Bold AJ (2020) Cardiac natriuretic peptides. Nat Rev Cardiol 17:698–717 32444692 10.1038/s41569-020-0381-0

[CR52] Guo X, Aviles G, Liu Y, Tian R, Unger BA, Lin Y-HT, Wiita AP, Xu K, Correia MA, Kampmann M (2020) Mitochondrial stress is relayed to the cytosol by an OMA1-DELE1-HRI pathway. Nature 579:427–432 32132707 10.1038/s41586-020-2078-2 PMC7147832

[CR53] Haeussler M, Schönig K, Eckert H, Eschstruth A, Mianné J, Renaud J-B, Schneider-Maunoury S, Shkumatava A, Teboul L, Kent J et al (2016) Evaluation of off-target and on-target scoring algorithms and integration into the guide RNA selection tool CRISPOR. Genome Biol 17:148 27380939 10.1186/s13059-016-1012-2 PMC4934014

[CR54] Han S, Lee M, Shin Y, Giovanni R, Chakrabarty RP, Herrerias MM, Dada LA, Flozak AS, Reyfman PA, Khuder B et al (2023) Mitochondrial integrated stress response controls lung epithelial cell fate. Nature 620:890–897 37558881 10.1038/s41586-023-06423-8 PMC10447247

[CR55] Hughes CS, Foehr S, Garfield DA, Furlong EE, Steinmetz LM, Krijgsveld J (2014) Ultrasensitive proteome analysis using paramagnetic bead technology. Mol Syst Biol 10:757 10.15252/msb.20145625 PMC4299378 25358341

[CR56] Indrieri A, Conte I, Chesi G, Romano A, Quartararo J, Tatè R, Ghezzi D, Zeviani M, Goffrini P, Ferrero I et al (2013) The impairment of HCCS leads to MLS syndrome by activating a non-canonical cell death pathway in the brain and eyes: hccsand noncanonical cell death. EMBO Mol Med 5:280–293 23239471 10.1002/emmm.201201739 PMC3569643

[CR57] Indrieri A, van Rahden VA, Tiranti V, Morleo M, Iaconis D, Tammaro R, D’Amato I, Conte I, Maystadt I, Demuth S et al (2012) Mutations in COX7B cause microphthalmia with linear skin lesions, an unconventional mitochondrial disease. Am J Hum Genet 91:942–949 23122588 10.1016/j.ajhg.2012.09.016 PMC3487127

[CR58] Jaberi E, Chitsazian F, Ali Shahidi G, Rohani M, Sina F, Safari I, Malakouti Nejad M, Houshmand M, Klotzle B, Elahi E (2013) The novel mutation p.Asp251Asn in the β-subunit of succinate-CoA ligase causes encephalomyopathy and elevated succinylcarnitine. J Hum Genet 58:526–530 23759946 10.1038/jhg.2013.45

[CR59] Jackson CB, Marmyleva A, Monteuuis G, Awadhpersad R, Mito T, Zamboni N, Tatsuta T, Vincent AE, Wang L, Khan NA et al (2025) De novo serine biosynthesis is protective in mitochondrial disease. Cell Rep 44:115710 40381195 10.1016/j.celrep.2025.115710

[CR60] Janer A, Antonicka H, Lalonde E, Nishimura T, Sasarman F, Brown GK, Brown RM, Majewski J, Shoubridge EA (2012) An RMND1 Mutation causes encephalopathy associated with multiple oxidative phosphorylation complex deficiencies and a mitochondrial translation defect. Am J Hum Genet 91:737–743 23022098 10.1016/j.ajhg.2012.08.020 PMC3484649

[CR61] Jiang M, Kauppila TES, Motori E, Li X, Atanassov I, Folz-Donahue K, Bonekamp NA, Albarran-Gutierrez S, Stewart JB, Larsson N-G (2017) Increased total mtDNA copy number cures male infertility despite unaltered mtDNA mutation load. Cell Metab 26:429–436.e4 28768180 10.1016/j.cmet.2017.07.003

[CR62] Jiang Y, Feng Y-P, Tang L-X, Yan Y-L, Bai J-W (2019) The protective role of NR4A3 in acute myocardial infarction by suppressing inflammatory responses via JAK2-STAT3/NF-κB pathway. Biochem Biophys Res Commun 517:697–702 31399192 10.1016/j.bbrc.2019.07.116

[CR63] John SP, Sun J, Carlson RJ, Cao B, Bradfield CJ, Song J, Smelkinson M, Fraser IDC (2018) IFIT1 exerts opposing regulatory effects on the inflammatory and interferon gene programs in LPS-activated human macrophages. Cell Rep 25:95–106.e6 30282041 10.1016/j.celrep.2018.09.002 PMC6492923

[CR64] Johnson JO, Glynn SM, Gibbs JR, Nalls MA, Sabatelli M, Restagno G, Drory VE, Chiò A, Rogaeva E, Traynor BJ (2014) Mutations in the CHCHD10 gene are a common cause of familial amyotrophic lateral sclerosis. Brain 137:e311 25261972 10.1093/brain/awu265 PMC4240285

[CR65] Kaspar S, Oertlin C, Szczepanowska K, Kukat A, Senft K, Lucas C, Brodesser S, Hatzoglou M, Larsson O, Topisirovic I et al (2021) Adaptation to mitochondrial stress requires CHOP-directed tuning of ISR. Sci Adv 7:eabf0971 34039602 10.1126/sciadv.abf0971 PMC8153728

[CR66] Kaufman BA, Durisic N, Mativetsky JM, Costantino S, Hancock MA, Grutter P, Shoubridge EA (2007) The mitochondrial transcription factor TFAM coordinates the assembly of multiple DNA molecules into nucleoid-like structures. Mol Biol Cell 18:3225–3236 17581862 10.1091/mbc.E07-05-0404 PMC1951767

[CR67] Kaukonen J, Juselius JK, Tiranti V, Kyttälä A, Zeviani M, Comi GP, Keränen S, Peltonen L, Suomalainen A (2000) Role of adenine nucleotide translocator 1 in mtDNA maintenance. Science 289:782–785 10926541 10.1126/science.289.5480.782

[CR68] Kimura T, Flynn CT, Alirezaei M, Sen GC, Whitton JL (2019) Biphasic and cardiomyocyte-specific IFIT activity protects cardiomyocytes from enteroviral infection. PLoS Pathog 15:e1007674 30958867 10.1371/journal.ppat.1007674 PMC6453442

[CR69] Köster J, Rahmann S (2012) Snakemake—a scalable bioinformatics workflow engine. Bioinformatics 28:2520–2522 22908215 10.1093/bioinformatics/bts480

[CR70] Labbé K, LeBon L, King B, Vu N, Stoops EH, Ly N, Lefebvre AEYT, Seitzer P, Krishnan S, Heo J-M et al (2024) Specific activation of the integrated stress response uncovers regulation of central carbon metabolism and lipid droplet biogenesis. Nat Commun 15:8301 39333061 10.1038/s41467-024-52538-5 PMC11436933

[CR71] Lam J, Katti P, Biete M, Mungai M, AshShareef S, Neikirk K, Garza Lopez E, Vue Z, Christensen TA, Beasley HK et al (2021) A universal approach to analyzing transmission electron microscopy with ImageJ. Cells 10:2177 34571826 10.3390/cells10092177 PMC8465115

[CR72] Larsson NG, Wang J, Wilhelmsson H, Oldfors A, Rustin P, Lewandoski M, Barsh GS, Clayton DA (1998) Mitochondrial transcription factor A is necessary for mtDNA maintenance and embryogenesis in mice. Nat Genet 18:231–236 9500544 10.1038/ng0398-231

[CR73] Leary SC, Cobine PA, Kaufman BA, Guercin G-H, Mattman A, Palaty J, Lockitch G, Winge DR, Rustin P, Horvath R et al (2007) The human cytochrome c oxidase assembly factors SCO1 and SCO2 have regulatory roles in the maintenance of cellular copper homeostasis. Cell Metab 5:9–20 17189203 10.1016/j.cmet.2006.12.001

[CR74] Lehtonen JM, Forsström S, Bottani E, Viscomi C, Baris OR, Isoniemi H, Höckerstedt K, Österlund P, Hurme M, Jylhävä J et al (2016) FGF21 is a biomarker for mitochondrial translation and mtDNA maintenance disorders. Neurology 87:2290–2299 27794108 10.1212/WNL.0000000000003374 PMC5270510

[CR75] Lei Y, Guerra Martinez C, Torres-Odio S, Bell SL, Birdwell CE, Bryant JD, Tong CW, Watson RO, West LC, West AP (2021) Elevated type I interferon responses potentiate metabolic dysfunction, inflammation, and accelerated aging in mtDNA mutator mice. Sci Adv 7:eabe7548 34039599 10.1126/sciadv.abe7548 PMC8153723

[CR76] Lei Y, VanPortfliet JJ, Chen Y-F, Bryant JD, Li Y, Fails D, Torres-Odio S, Ragan KB, Deng J, Mohan A et al (2023) Cooperative sensing of mitochondrial DNA by ZBP1 and cGAS promotes cardiotoxicity. Cell 186:3013–3032.e22 37352855 10.1016/j.cell.2023.05.039 PMC10330843

[CR77] Lepelley A, Wai T, Crow YJ (2021) Mitochondrial nucleic acid as a driver of pathogenic type I interferon induction in Mendelian disease. Front Immunol 12:729763 34512665 10.3389/fimmu.2021.729763 PMC8428523

[CR78] Li Y, Li C, Xue P, Zhong B, Mao A-P, Ran Y, Chen H, Wang Y-Y, Yang F, Shu H-B (2009) ISG56 is a negative-feedback regulator of virus-triggered signaling and cellular antiviral response. Proc Natl Acad Sci USA 106:7945–7950 19416887 10.1073/pnas.0900818106 PMC2683125

[CR79] Liao Y, Smyth GK, Shi W (2014) featureCounts: an efficient general purpose program for assigning sequence reads to genomic features. Bioinformatics 30:923–930 24227677 10.1093/bioinformatics/btt656

[CR80] Lill R, Stuart RA, Drygas ME, Nargang FE, Neupert W (1992) Import of cytochrome c heme lyase into mitochondria: a novel pathway into the intermembrane space. EMBO J 11:449–456 1371459 10.1002/j.1460-2075.1992.tb05074.x PMC556474

[CR81] Lin H, Miyauchi K, Harada T, Okita R, Takeshita E, Komaki H, Fujioka K, Yagasaki H, Goto Y-I, Yanaka K et al (2018) CO2-sensitive tRNA modification associated with human mitochondrial disease. Nat Commun 9:1875 29760464 10.1038/s41467-018-04250-4 PMC5951830

[CR82] Lin H-P, Petersen JD, Gilsrud AJ, Madruga A, D’Silva TM, Huang X, Shammas MK, Randolph NP, Johnson KR, Li Y et al (2024) DELE1 maintains muscle proteostasis to promote growth and survival in mitochondrial myopathy. EMBO J 43:5548–5585 39379554 10.1038/s44318-024-00242-x PMC11574132

[CR83] Lindsey ML, Usselman CW, Ripplinger CM, Carter JR, DeLeon-Pennell KY (2024) Sex as a biological variable for cardiovascular physiology. Am J Physiol Heart Circ Physiol 326:H459–H469 38099847 10.1152/ajpheart.00727.2023 PMC11219053

[CR84] Liu Y-T, Huang X, Nguyen D, Shammas MK, Wu BP, Dombi E, Springer DA, Poulton J, Sekine S, Narendra DP (2020) Loss of CHCHD2 and CHCHD10 activates OMA1 peptidase to disrupt mitochondrial cristae phenocopying patient mutations. Hum Mol Genet 29:1547–1567 32338760 10.1093/hmg/ddaa077 PMC7268789

[CR85] Livak KJ, Schmittgen TD (2001a) Analysis of relative gene expression data using real-time quantitative PCR and the 2 − ΔΔCT method. Methods 25:402–408 11846609 10.1006/meth.2001.1262

[CR86] Livak KJ, Schmittgen TD (2001b) Analysis of relative gene expression data using real-time quantitative PCR and the 2(-Delta Delta C(T)) method. Methods 25:402–408 11846609 10.1006/meth.2001.1262

[CR87] Love MI, Huber W, Anders S (2014) Moderated estimation of fold change and dispersion for RNA-seq data with DESeq2. Genome Biol 15:550 25516281 10.1186/s13059-014-0550-8 PMC4302049

[CR88] Lv G, Sayles NM, Huang Y, Mancinelli C, McAvoy K, Shneider NA, Manfredi G, Kawamata H, Eliezer D (2025) Amyloid fibril structures link CHCHD10 and CHCHD2 to neurodegeneration. Nat Commun 16:7121 40753073 10.1038/s41467-025-62149-3 PMC12318133

[CR89] Milenkovic D, Misic J, Hevler JF, Molinié T, Chung I, Atanassov I, Li X, Filograna R, Mesaros A, Mourier A et al (2023) Preserved respiratory chain capacity and physiology in mice with profoundly reduced levels of mitochondrial respirasomes. Cell Metab 35:1799–1813.e7 37633273 10.1016/j.cmet.2023.07.015

[CR90] Miller C, Saada A, Shaul N, Shabtai N, Ben-Shalom E, Shaag A, Hershkovitz E, Elpeleg O (2004) Defective mitochondrial translation caused by a ribosomal protein (MRPS16) mutation. Ann Neurol 56:734–738 15505824 10.1002/ana.20282

[CR91] Monzel AS, Enríquez JA, Picard M (2023) Multifaceted mitochondria: moving mitochondrial science beyond function and dysfunction. Nat Metab 5:546–562 37100996 10.1038/s42255-023-00783-1 PMC10427836

[CR92] Müller K, Andersen PM, Hübers A, Marroquin N, Volk AE, Danzer KM, Meitinger T, Ludolph AC, Strom TM, Weishaupt JH (2014) Two novel mutations in conserved codons indicate that CHCHD10 is a gene associated with motor neuron disease. Brain 137:e309 25113787 10.1093/brain/awu227

[CR93] Nambot S, Gavrilov D, Thevenon J, Bruel AL, Bainbridge M, Rio M, Goizet C, Rötig A, Jaeken J, Niu N et al (2017) Further delineation of a rare recessive encephalomyopathy linked to mutations in GFER thanks to data sharing of whole exome sequencing data. Clin Genet 92:188–198 28155230 10.1111/cge.12985

[CR94] Nargund AM, Pellegrino MW, Fiorese CJ, Baker BM, Haynes CM (2012) Mitochondrial import efficiency of ATFS-1 regulates mitochondrial UPR activation. Science 337:587–590 22700657 10.1126/science.1223560 PMC3518298

[CR95] Ng MYW, Wai T, Simonsen A (2021) Quality control of the mitochondrion. Dev Cell 56:881–905 33662258 10.1016/j.devcel.2021.02.009

[CR96] Nickel AG, von Hardenberg A, Hohl M, Löffler JR, Kohlhaas M, Becker J, Reil J-C, Kazakov A, Bonnekoh J, Stadelmaier M et al (2015) Reversal of mitochondrial transhydrogenase causes oxidative stress in heart failure. Cell Metab 22:472–484 26256392 10.1016/j.cmet.2015.07.008

[CR97] Nolte H, MacVicar TD, Tellkamp F, Krüger M (2018) Instant Clue: a software suite for interactive data visualization and analysis. Sci Rep 8:12648 10.1038/s41598-018-31154-6 PMC6107636 30140043

[CR98] Oka T, Hikoso S, Yamaguchi O, Taneike M, Takeda T, Tamai T, Oyabu J, Murakawa T, Nakayama H, Nishida K et al (2012) Mitochondrial DNA that escapes from autophagy causes inflammation and heart failure. Nature 485:251–255 22535248 10.1038/nature10992 PMC3378041

[CR99] Oquendo CE, Antonicka H, Shoubridge EA, Reardon W, Brown GK (2004) Functional and genetic studies demonstrate that mutation in the COX15 gene can cause Leigh syndrome. J Med Genet 41:540–544 15235026 10.1136/jmg.2003.017426 PMC1735852

[CR100] Palmieri L, Alberio S, Pisano I, Lodi T, Meznaric-Petrusa M, Zidar J, Santoro A, Scarcia P, Fontanesi F, Lamantea E et al (2005) Complete loss-of-function of the heart/muscle-specific adenine nucleotide translocator is associated with mitochondrial myopathy and cardiomyopathy. Hum Mol Genet 14:3079–3088 16155110 10.1093/hmg/ddi341

[CR101] Papadopoulou LC, Sue CM, Davidson MM, Tanji K, Nishino I, Sadlock JE, Krishna S, Walker W, Selby J, Glerum DM et al (1999) Fatal infantile cardioencephalomyopathy with COX deficiency and mutations in SCO2, a COX assembly gene. Nat Genet 23:333–337 10545952 10.1038/15513

[CR102] Patitucci C, Hernández-Camacho JD, Vimont E, Yde S, Cokelaer T, Chaze T, Giai Gianetto Q, Matondo M, Gazi A, Nemazanyy I et al (2023) Mtfp1 ablation enhances mitochondrial respiration and protects against hepatic steatosis. Nat Commun 14:8474 38123539 10.1038/s41467-023-44143-9 PMC10733382

[CR103] Penttilä S, Jokela M, Bouquin H, Saukkonen AM, Toivanen J, Udd B (2015) Late onset spinal motor neuronopathy is caused by mutation in CHCHD10. Ann Neurol 77:163–172 25428574 10.1002/ana.24319

[CR104] Picard M, Shirihai OS (2022) Mitochondrial signal transduction. Cell Metab 34:1620–1653 36323233 10.1016/j.cmet.2022.10.008 PMC9692202

[CR105] Pierrel F, Bestwick ML, Cobine PA, Khalimonchuk O, Cricco JA, Winge DR (2007) Coa1 links the Mss51 post-translational function to Cox1 cofactor insertion in cytochrome c oxidase assembly. EMBO J 26:4335–4346 17882260 10.1038/sj.emboj.7601861 PMC2034670

[CR106] Qu X, Liu Y, Cao D, Chen J, Liu Z, Ji H, Chen Y, Zhang W, Zhu P, Xiao D et al (2019) BMP10 preserves cardiac function through its dual activation of SMAD-mediated and STAT3-mediated pathways. J Biol Chem 294:19877–19888 31712309 10.1074/jbc.RA119.010943 PMC6937579

[CR107] Rath S, Sharma R, Gupta R, Ast T, Chan C, Durham TJ, Goodman RP, Grabarek Z, Haas ME, Hung WHW et al (2021) MitoCarta3.0: an updated mitochondrial proteome now with sub-organelle localization and pathway annotations. Nucleic Acids Res 49:D1541–D1547 33174596 10.1093/nar/gkaa1011 PMC7778944

[CR108] Ropert B, Bannwarth S, Genin EC, Vaillant-Beuchot L, Lacas-Gervais S, Hounoum BM, Bernardin A, Dinh N, Mauri-Crouzet A, D’Elia M-A et al (2024) Nifuroxazide rescues the deleterious effects due to CHCHD10-associated MICOS defects in disease models. Brain 148:1665–1679 10.1093/brain/awae348 39478664

[CR109] Russell OM, Gorman GS, Lightowlers RN, Turnbull DM (2020) Mitochondrial diseases: hope for the future. Cell 181:168–188 32220313 10.1016/j.cell.2020.02.051

[CR110] San Francisco B, Bretsnyder EC, Kranz RG (2013) Human mitochondrial holocytochrome c synthase’s heme binding, maturation determinants, and complex formation with cytochrome c. Proc Natl Acad Sci USA 110:E788–E797 23150584 10.1073/pnas.1213897109 PMC3587199

[CR111] Sauer J-D, Sotelo-Troha K, von Moltke J, Monroe KM, Rae CS, Brubaker SW, Hyodo M, Hayakawa Y, Woodward JJ, Portnoy DA et al (2011) The N-ethyl-N-nitrosourea-induced Goldenticket mouse mutant reveals an essential function of Sting in the in vivo interferon response to Listeria monocytogenes and cyclic dinucleotides. Infect Immun 79:688–694 21098106 10.1128/IAI.00999-10 PMC3028833

[CR112] Sayles NM, Southwell N, McAvoy K, Kim K, Pesini A, Anderson CJ, Quinzii C, Cloonan S, Kawamata H, Manfredi G (2022) Mutant CHCHD10 causes an extensive metabolic rewiring that precedes OXPHOS dysfunction in a murine model of mitochondrial cardiomyopathy. Cell Rep 38:110475 35263592 10.1016/j.celrep.2022.110475 PMC9013208

[CR113] Sekine Y, Houston R, Eckl E-M, Fessler E, Narendra DP, Jae LT, Sekine S (2023) A mitochondrial iron-responsive pathway regulated by DELE1. Mol Cell 83:2059–2076.e6 37327776 10.1016/j.molcel.2023.05.031 PMC10329284

[CR114] Seligman AM, Wasserkrug HL, Hanker JS (1966) A new staining method (OTO) for enhancing contrast of lipid-containing membranes and droplets in osmium tetroxide-fixed tissue with osmiophilic thiocarbohydrazide(TCH). J Cell Biol 30:424–432 4165523 10.1083/jcb.30.2.424 PMC2106998

[CR115] Shammas MK, Huang X, Wu BP, Fessler E, Song IY, Randolph NP, Li Y, Bleck CK, Springer DA, Fratter C et al (2022) OMA1 mediates local and global stress responses against protein misfolding in CHCHD10 mitochondrial myopathy. J Clin Invest 132:e157504 35700042 10.1172/JCI157504 PMC9282932

[CR116] Shammas MK, Nie Y, Gilsrud A, Huang X, Narendra DP, Chinnery PF (2023) CHCHD10 mutations induce tissue-specific mitochondrial DNA deletions with a distinct signature. Hum Mol Genet 33:91–101 10.1093/hmg/ddad161 PMC10729859 37815936

[CR117] Sliter DA, Martinez J, Hao L, Chen X, Sun N, Fischer TD, Burman JL, Li Y, Zhang Z, Narendra DP et al (2018) Parkin and PINK1 mitigate STING-induced inflammation. Nature 561:258–262 30135585 10.1038/s41586-018-0448-9 PMC7362342

[CR118] Southwell N, Manzo O, Bacman S, Zhao D, Sayles NM, Dash J, Fujita K, D’Aurelio M, Di Lorenzo A, Manfredi G et al (2024) High fat diet ameliorates mitochondrial cardiomyopathy in CHCHD10 mutant mice. EMBO Mol Med 16:1352–1378 38724625 10.1038/s44321-024-00067-5 PMC11178915

[CR119] Stroud DA, Maher MJ, Lindau C, Vögtle F-N, Frazier AE, Surgenor E, Mountford H, Singh AP, Bonas M, Oeljeklaus S et al (2015) COA6 is a mitochondrial complex IV assembly factor critical for biogenesis of mtDNA-encoded COX2. Hum Mol Genet 24:5404–5415 26160915 10.1093/hmg/ddv265

[CR120] Swaminathan AB, Soma S, Vicary AC, Zulkifli M, Kaur H, Gohil VM (2022) A yeast suppressor screen links Coa4 to the mitochondrial copper delivery pathway for cytochrome c oxidase. Genetics 221:iyac090 10.1093/genetics/iyac090 PMC9339329 35666203

[CR121] Tarnopolsky MA, Bourgeois JM, Fu M-H, Kataeva G, Shah J, Simon DK, Mahoney D, Johns D, MacKay N, Robinson BH (2004) Novel SCO2 mutation (G1521A) presenting as a spinal muscular atrophy type I phenotype. Am J Med Genet A 125A:310–314 14994243 10.1002/ajmg.a.20466

[CR122] Timón-Gómez A, Garlich J, Stuart RA, Ugalde C, Barrientos A (2020) Distinct roles of mitochondrial HIGD1A and HIGD2A in respiratory complex and supercomplex biogenesis. Cell Rep 31:107607 32375044 10.1016/j.celrep.2020.107607

[CR123] Valnot I, Osmond S, Gigarel N, Mehaye B, Amiel J, Cormier-Daire V, Munnich A, Bonnefont JP, Rustin P, Rötig A (2000b) Mutations of the SCO1 gene in mitochondrial cytochrome c oxidase deficiency with neonatal-onset hepatic failure and encephalopathy. Am J Hum Genet 67:1104–1109 11013136 10.1016/s0002-9297(07)62940-1 PMC1288552

[CR124] Valnot I, von, Kleist-Retzow JC, Barrientos A, Gorbatyuk M, Taanman JW, Mehaye B, Rustin P, Tzagoloff A, Munnich A, Rötig A (2000a) A mutation in the human heme A: farnesyltransferase gene (COX10) causes cytochrome c oxidase deficiency. Hum Mol Genet 9:1245–1249 10767350 10.1093/hmg/9.8.1245

[CR125] Vela-Sebastián A, Bayona-Bafaluy P, Pacheu-Grau D (2024) ISR pathway contribution to tissue specificity of mitochondrial diseases. Trends Endocrinol Metab 35:851–853 10.1016/j.tem.2024.05.001 38806299

[CR126] Villani G, Attardi G (1997) In vivo control of respiration by cytochrome c oxidase in wild-type and mitochondrial DNA mutation-carrying human cells. Proc Natl Acad Sci USA 94:1166–1171 9037024 10.1073/pnas.94.4.1166 PMC19762

[CR127] Wai T (2024) Is mitochondrial morphology important for cellular physiology?. Trends Endocrinol Metab 35:854–871 38866638 10.1016/j.tem.2024.05.005

[CR128] Wai T, García-Prieto J, Baker MJ, Merkwirth C, Benit P, Rustin P, Rupérez FJ, Barbas C, Ibañez B, Langer T (2015) Imbalanced OPA1 processing and mitochondrial fragmentation cause heart failure in mice. Science 350:aad0116 26785494 10.1126/science.aad0116

[CR129] Wai T, Saita S, Nolte H, Müller S, König T, Richter-Dennerlein R, Sprenger H-G, Madrenas J, Mühlmeister M, Brandt U et al (2016) The membrane scaffold SLP2 anchors a proteolytic hub in mitochondria containing PARL and the i-AAA protease YME1L. EMBO Rep 17:1844–1856 27737933 10.15252/embr.201642698 PMC5283581

[CR130] Wallace DC, Fan W, Procaccio V (2010) Mitochondrial energetics and therapeutics. Annu Rev Pathol 5:297–348 20078222 10.1146/annurev.pathol.4.110807.092314 PMC3245719

[CR131] Wang D, Wang J, Bonamy GMC, Meeusen S, Brusch RG, Turk C, Yang P, Schultz PG (2012) A small molecule promotes mitochondrial fusion in mammalian cells. Angew Chem Int Ed Engl 51:9302–9305 22907892 10.1002/anie.201204589

[CR132] Wittig I, Braun H-P, Schägger H (2006) Blue native PAGE. Nat Protoc 1:418–428 17406264 10.1038/nprot.2006.62

[CR133] Yagensky O, Kohansal-Nodehi M, Gunaseelan S, Rabe T, Zafar S, Zerr I, Härtig W, Urlaub H, Chua JJ (2019) Increased expression of heme-binding protein 1 early in Alzheimer’s disease is linked to neurotoxicity. eLife 8:e47498 10.7554/eLife.47498 PMC6739868 31453805

[CR134] Yu C-H, Davidson S, Harapas CR, Hilton JB, Mlodzianoski MJ, Laohamonthonkul P, Louis C, Low RRJ, Moecking J, De Nardo D et al (2020) TDP-43 triggers mitochondrial DNA release via mPTP to activate cGAS/STING in ALS. Cell 183:636–649.e18 33031745 10.1016/j.cell.2020.09.020 PMC7599077

[CR135] Zachos KA, Gamboa JA, Dewji AS, Lee J, Brijbassi S, Andreazza AC (2024) The interplay between mitochondria, the gut microbiome and metabolites and their therapeutic potential in primary mitochondrial disease. Front Pharm 15:1428242 10.3389/fphar.2024.1428242 PMC11306032 39119601

