# Supplementary material for: Mutant CHCHD10 disrupts cytochrome c oxidation and activates mitochondrial retrograde signaling
Source: EMBO Mol Med. 2025 Dec 19;18(2):542–74. doi: 10.1038/s44321-025-00358-5 (PMC12905356; doi:10.1038/s44321-025-00358-5)
Supplement: Supplementary file 1 — Appendix [file 44321_2025_358_MOESM1_ESM.pdf]

Title

Mutant CHCHD10 disrupts cytochrome *c* oxidation and activates mitochondrial retrograde signaling.

Authors

Márcio Augusto Campos-Ribeiro[1], Erminia Donnarumma[1], Hendrik Nolte[2], Paul Cobine[3], Elodie Vimont[1], Dusanka Milenkovic[2], Juan Diego Hernandez-Camacho[1], Francina Langa Vives[4], Etienne Kornobis[5], Esthel Pénard[6], Sonny Yde[1], Thomas Langer[2], Véronique Paquis-Flucklinger[7], Timothy Wai[1*]

Appendix

Appendix Figures S1 – Page 2
Appendix Figures S2 – Page 3
Appendix Figures S3 – Page 4
Appendix Figures S4 – Page 5
Appendix Figures S5 – Page 6

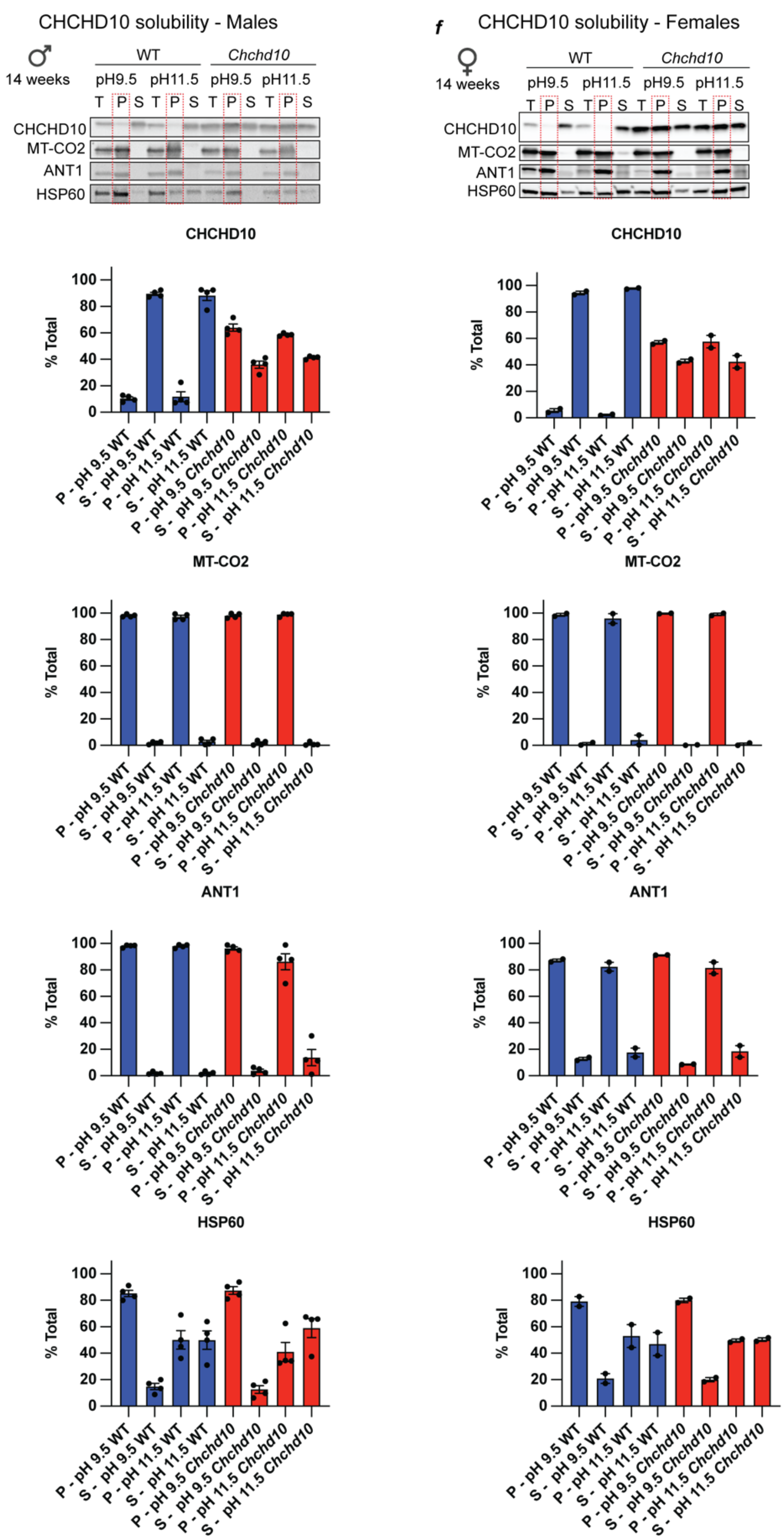

Appendix Figure S1 – Alkaline carbonate (Na_2CO_3) extraction of cardiac mitochondria
Alkaline carbonate (Na_2CO_3) extraction of cardiac mitochondria (corresponding to Figure 1f) performed on WT (blue) and *Chchd10* (red) mutant male (n=4, left) and female (n=2, right) mice at 14 weeks of age were analyzed by immunoblotting with indicated antibodies. Insoluble pellet (P), and soluble supernatant (S) fractions were quantified by densitometry relative to Total (T). Data represent mean ± SEM.

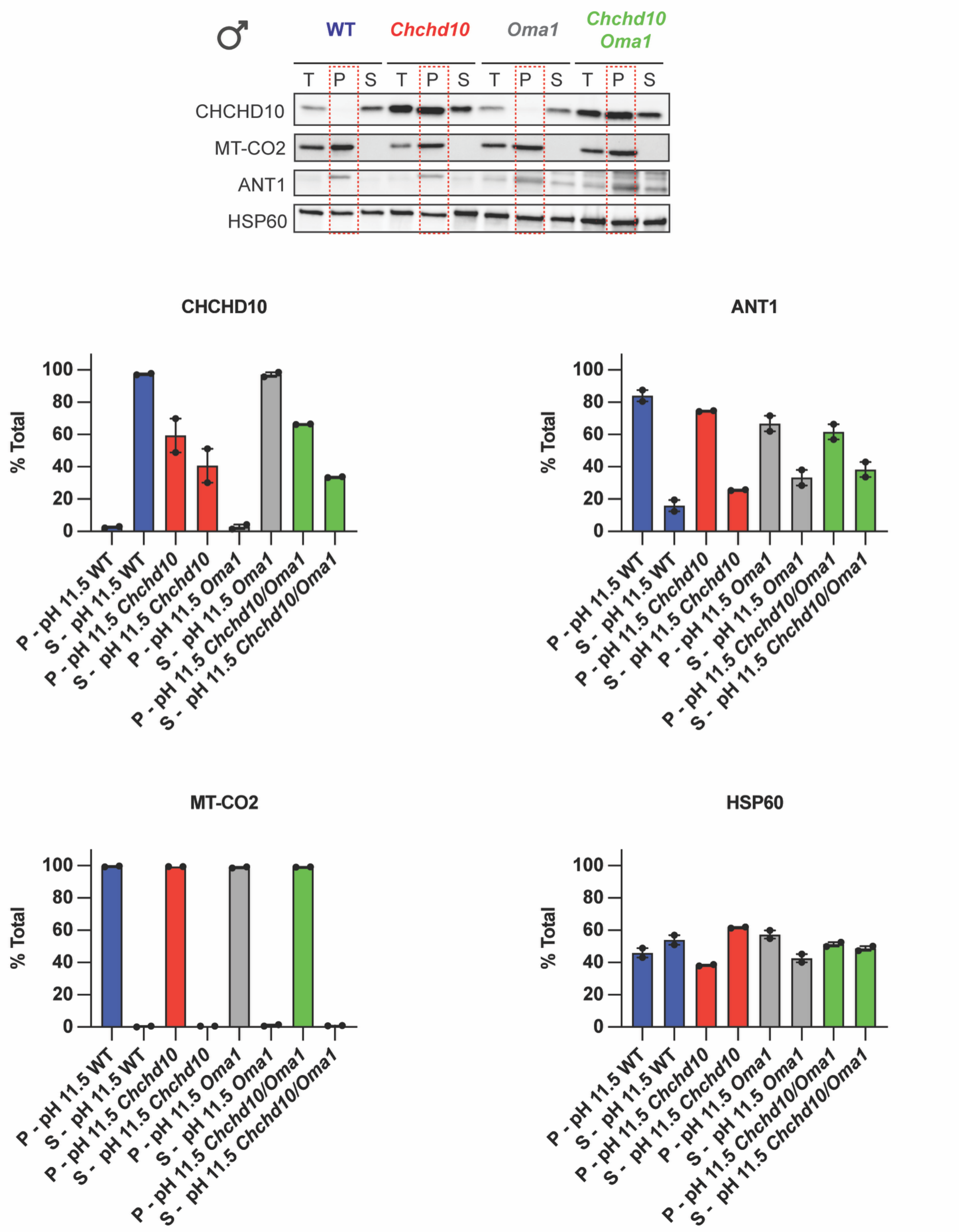

Appendix Figure S2 – Alkaline carbonate (Na_2CO_3) extraction of cardiac mitochondria

Alkaline carbonate (Na_2CO_3) extraction of cardiac mitochondria (corresponding to Figure 3e performed on WT (blue, n=2), *Chchd10* (red, n=2), Oma1 (grey, n=2), and Chchd10/Oma1 (green, n=2) mutant male mice at 14 weeks of age were analyzed by immunoblotting with indicated antibodies. Insoluble pellet (P), and soluble supernatant (S) fractions were quantified by densitometry relative to Total (T).

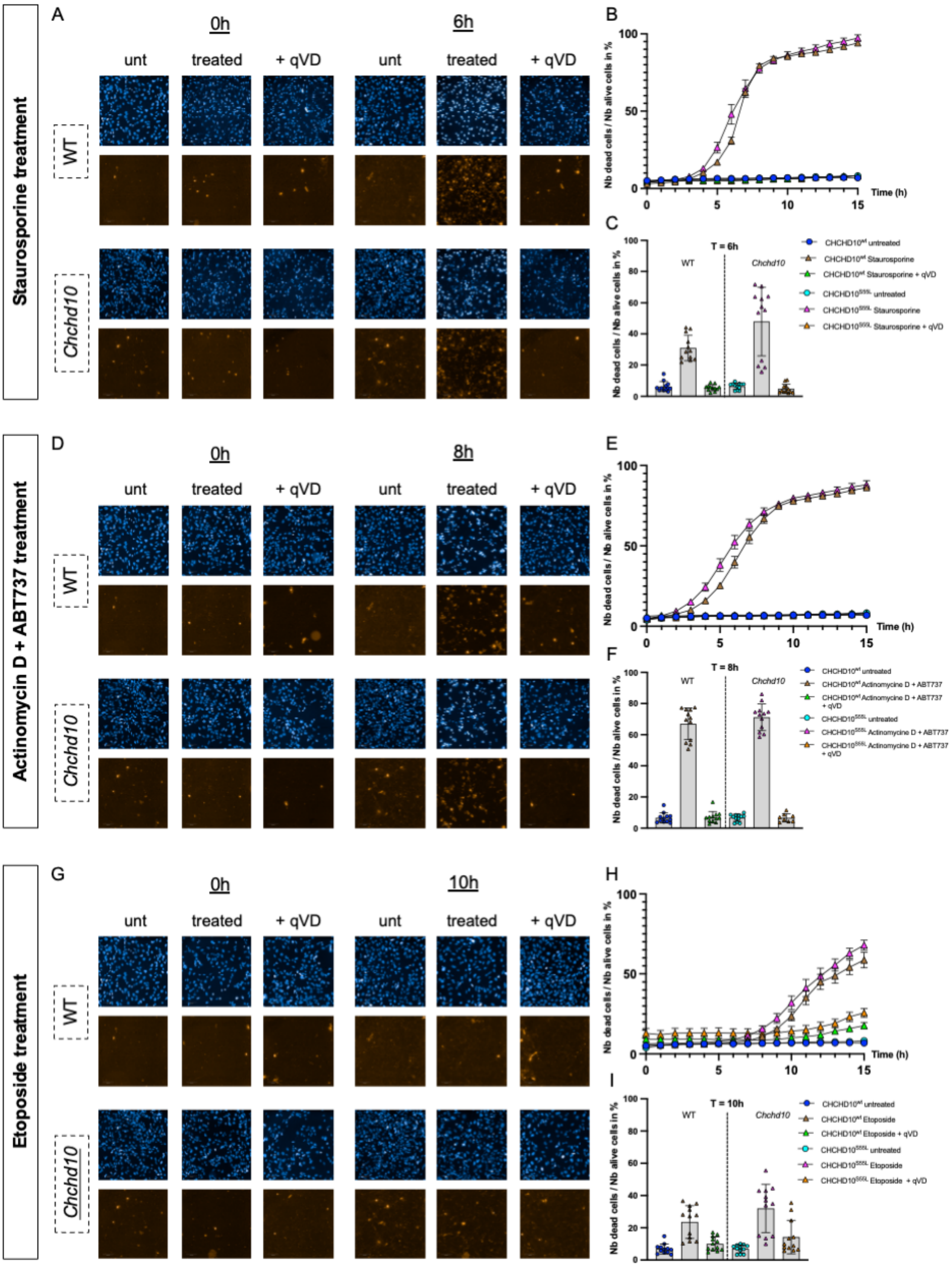

Appendix

Figure S3 – Cell death sensitivity in MEFs cultured in glucose media
3 independent WT and 3 independent *Chchd10*$^{S55L/+}$ mouse embryonic fibroblasts (MEF) immortalized lines were grown in glucose-containing media either untreated or treated with 1uM staurosporine (A-C), 1 μM actinomycin D + 10μM ABT737 (D-F) or 500μM etoposide (G-I) to induce cell death. The caspase inhibitor qVD (20μM) was also added to prevent caspase-dependent cell death. All treatments were performed simultaneously in a 96-well plate and the untreated (unt) representative images in A for 0h are reused in D, and G. The number of dead cells is represented as a percentage of dead, propidium iodide (PI)-positive cells relative to total cells (NucBlue+) over a 15-h period. Each biological replicate was performed in technical duplicates to calculate the mean value for each time point. Each technical replicate represents the measurement of 482-3734 cells per well. B, E, and H, data represent mean of 3 biological replicates ± SEM. All technical replicates are shown in C, F, and I, for the indicated time point representing mean value ± SEM. The untreated CHCHD10^{WT} (blue circles) values in B and C are reused in E, F, H, and I.

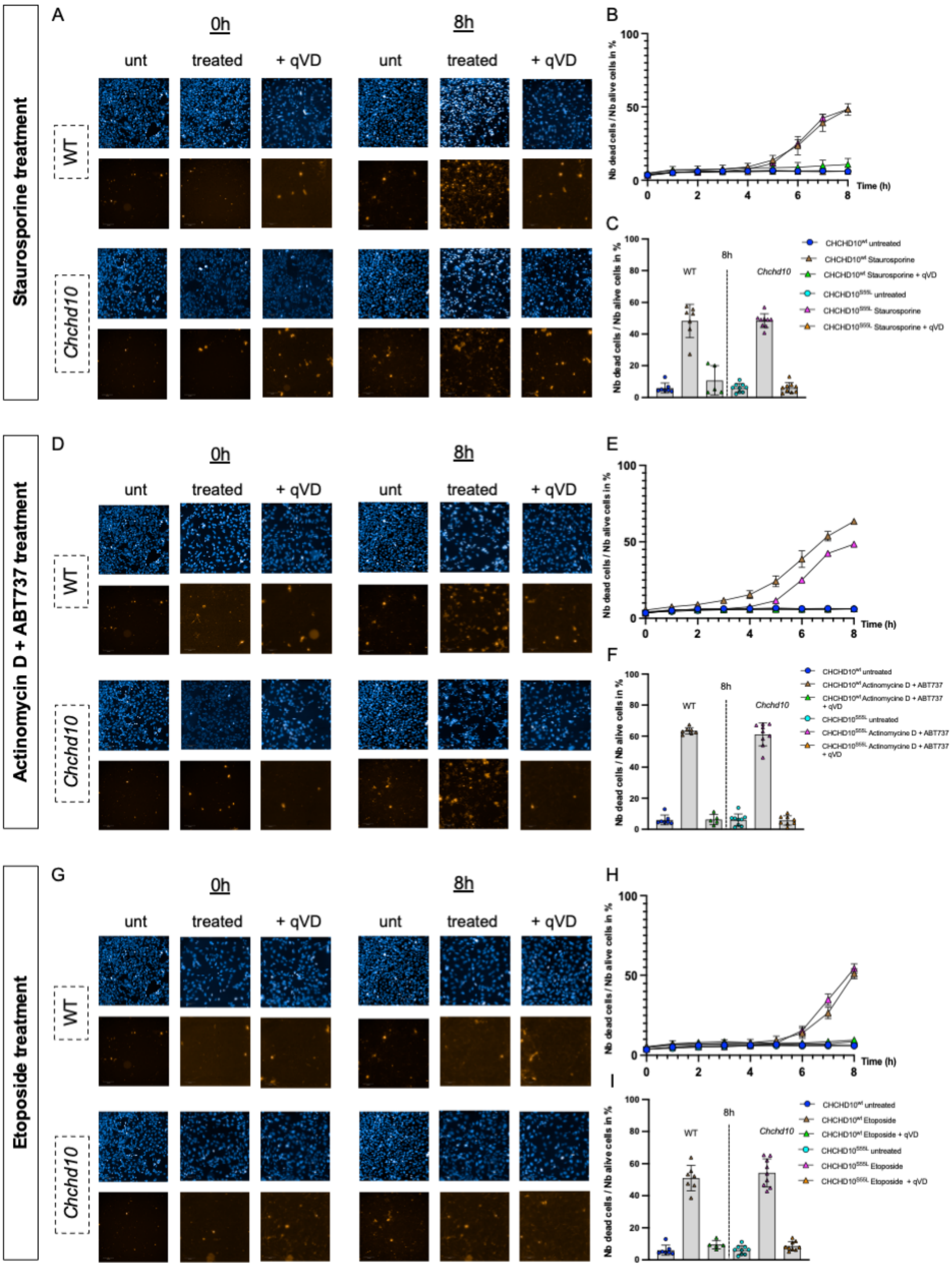

Appendix

Figure S4 – Cell death sensitivity in MEFs cultured in galactose media

3 independent WT and 3 independent *Chchd10*$^{S55L/+}$ mouse embryonic fibroblasts (MEF) immortalized lines were grown in galactose-containing media either untreated or treated with 1uM staurosporine (A-C), 1 μM actinomycin D + 10μM ABT737 (D-F) or 500μM etoposide (G-I) to induce cell death. The caspase inhibitor qVD (20μM) was also added to prevent caspase-dependent cell death. All treatments were performed simultaneously in a 96-well plate and the untreated (unt) representative images in A for 0h are reused in D, and G. The number of dead cells is represented as a percentage of dead, propidium iodide (PI)-positive cells relative to total cells (NucBlue+) over a 15-h period. Each biological replicate was performed in at least technical duplicates to calculate the mean value for each time point. Each technical replicate represents the measurement of 965-2776 cells per well. B, E, and H, data represent mean of 3 biological replicates ± SEM. All technical replicates are shown in C, F, and I, for the indicated time point representing mean value ± SEM. The untreated CHCHD10^{WT} (blue circles) values in B and C are reused in E, F, H, and I.

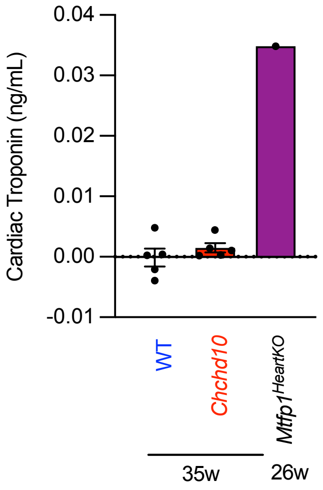

Appendix Figure S5 – Cardiac troponin levels in wild type and Chchd10 mutant mice
Circulating levels of cardiac troponin measured by ELISA in serum isolated by submandibular sampling of wild type (WT, blue, n=5) and *Chchd10*$^{S55L/+}$ (Chchd10, red, n=5) mutant male mice. Data represent mean ± SEM. Serum from cardiomyocyte-specific Mtfp1 knockout (*Mtfp1*^{HeartKO}) mouse suffering from dilated cardiomyopathy from a previous study(Donnarumma *et al*, 2022) was used as a positive control (purple).

References

Donnarumma E, Kohlhaas M, Vimont E, Kornobis E, Chaze T, Gianetto QG, Matondo M, Moya-Nilges M, Maack C & Wai T (2022) Mitochondrial Fission Process 1 controls inner membrane integrity and protects against heart failure. *Nat Commun* 13: 1–24
