# Supplementary material for: Mutant CHCHD10 disrupts cytochrome c oxidation and activates mitochondrial retrograde signaling
Source: EMBO Mol Med. 2025 Dec 19;18(2):542–74. doi: 10.1038/s44321-025-00358-5 (PMC12905356; doi:10.1038/s44321-025-00358-5)
Supplement: Supplementary file 17 — Expanded View Figures [file 44321_2025_358_MOESM17_ESM.pdf]

Expanded View Figures

Figure EV1. Cardiac remodeling in *Chchd10* mutant hearts.

(**A**) Diastolic (IVSd, mm) and Systolic interventricular septum thickness (IVSs, mm) and Diastolic (LVPWd, mm) and Systolic left ventricle posterior wall thickness (LVPWs, mm) of WT (blue, *n* = 3–9) and *Chchd10* (red, *n* = 3–8) male mice in Fig. 1C. Data represent mean ± SEM. One-way ANOVA, *$P = 0.046910$. (**B**) Diastolic (IVSd, mm) and Systolic interventricular septum thickness (IVSs, mm) and Diastolic (LVPWd, mm) and Systolic left ventricle posterior wall thickness (LVPWs, mm) of WT (blue, *n* = 4–9) and *Chchd10^{S55L}* (red, *n* = 6–7) female mice in Fig. 1C. Data represent mean ± SEM. One-way ANOVA, IVSs; *$P = 0.023457$, LVPWd; *$P = 0.008310$. (**C**) Kaplan–Meier survival curve of WT (blue, *n* = 10) and *Chchd10* (red, *n* = 9) male mice (left) and WT (blue, *n* = 13) and *Chchd10* (red, *n* = 12) female mice (right). Dotted line (gray) represents median lifespan of *Chchd10* mice (male; 62 weeks, Log-rank test, $P = 0.0006$ female 57 weeks, Log-rank test, $P < 0.0001$). (**D**) Alkaline carbonate (Na_2CO_3) extraction of cardiac mitochondria performed on WT and *Chchd10* mutant male mice at 7 weeks of age. Total (T), insoluble pellet (P), and soluble supernatant (S) fractions were analyzed by immunoblotting with the indicated antibodies. (**E**) Analysis of integrated stress response (ISR) genes *Atf4*, *Atf5*, *Trib3*, *Mthfd2*, *Phgdh*, *Asns*, *Aldh18a1*, and *Fgf21* via qRT-PCR of total cardiac biopsies from wild-type (blue, *n* = 3) and *Chchd10* (red, *n* = 3) male and female mice at the indicated ages. Data are relative mean fold changes ± SEM, one-way ANOVA. *Atf4*: WT vs *Chchd10* male ****$P < 0.0001$, WT vs *Chchd10* female ****$P < 0.0001$, *Atf5*: WT vs *Chchd10* male ****$P < 0.0001$, WT vs *Chchd10* female ****$P < 0.0001$, *Trib3*: WT vs *Chchd10* male **$P = 0.0030$, WT vs *Chchd10* female ****$P < 0.0001$, Chchd10 male vs *Chchd10* female ***$P = 0.0008$, *Mthfd2*; WT vs *Chchd10* male ****$P < 0.0001$, WT vs *Chchd10* female ****$P < 0.0001$, Chchd10 male vs *Chchd10* female ****$P < 0.0001$, *Phgdh*; WT vs *Chchd10* male ****$P < 0.0001$, WT vs *Chchd10* female ****$P < 0.0001$, *Asns*; WT vs *Chchd10* male ***$P = 0.003$, WT vs *Chchd10* female ****$P = 0.0002$, *Alsh18a1*; WT vs *Chchd10* male ****$P < 0.0001$, WT vs *Chchd10* female ****$P < 0.0001$, *Fgf21*; WT vs *Chchd10* male **$P = 0.0016$, WT vs *Chchd10* female **$P < 0.0031$. (**F**) Venn diagram of differentially expressed genes (DEGs) in male *Chchd10* versus WT mice. Bulk RNA-seq was performed on cardiac biopsies (*n* = 3) at 14 weeks of age. 955 genes were upregulated in male *Chchd10* vs wild-type (WT) hearts and 655 genes were downregulated. Reactome, KEGG, and Gene Ontology (GO) pathway enrichment performed with Enrichr. (**G**) Venn diagram of differentially expressed genes (DEGs) in female *Chchd10* versus WT mice. Bulk RNA-seq was performed on cardiac biopsies (*n* = 3) at 14 weeks of age. 681 genes were upregulated in female *Chchd10* vs wild-type (WT) hearts and 567 genes were downregulated. Reactome, KEGG, and Gene Ontology (GO) pathway enrichment performed with Enrichr. (**H**) Quantification of mitochondrial DNA (mtDNA) in cardiac biopsies from male WT (blue, *n* = 3), *Chchd10* (red, *n* = 3) mice at 7, 14, and 35 weeks. Primers directed at mtDNA encoding 16 s rRNA and β-actin for nDNA were used. Data are means ± SEM, multiple unpaired *t* test *P* values indicated. (**I**) Whole-body deletion of *Sting* in *Chchd10* mice. Generation of the *Chchd10$^{S55L/+}$Sting$^{Gt/Gt}$* mice were generated by intercrossing *Chchd10$^{S55L/+}$* (*Chchd10*) with *Sting$^{Gt/Gt}$* mice lacking functional STING (*Sting*). (**J**) Left ventricular ejection fraction (% LVEF) of *Sting* (*Sting$^{Gt/Gt}$*, orange, *n* = 6) and *Chchd10/Sting* (*Chchd10$^{S55L/+}$Sting$^{Gt/Gt}$*, purple, *n* = 8) male mice at 38 weeks (left) and 48 weeks (right) of age. Data represent mean ± SEM. Student's *t* test, **$P < 0.01$, ns=not significant. Dotted line represents *Chchd10*% LVEF at 35 weeks. (**K**) Body mass of *Sting* (*Sting$^{Gt/Gt}$*, orange, *n* = 3–12), *Chchd10/Sting* (*Chchd10$^{S55L/+}$Sting$^{Gt/Gt}$*, purple= *n* = 3–14) male mice. Data are means ± SEM, 2-tailed unpaired Student's *t* test used to identify significant differences ($P < 0.05$). Dotted gray line represents the age after which body mass differences between WT and *Chchd10* male (17 weeks) mice. (**L**) Kaplan–Meier survival curve of *Sting* (orange, *n* = 14), *Chchd10/Sting* (purple = *n* = 10) male mice. Dotted line (gray) represents median lifespan of *Chchd10* mice Log-rank test *Chchd10* vs *Chchd10/Sting*, $P = 0.002$. Source data are available online for this figure.

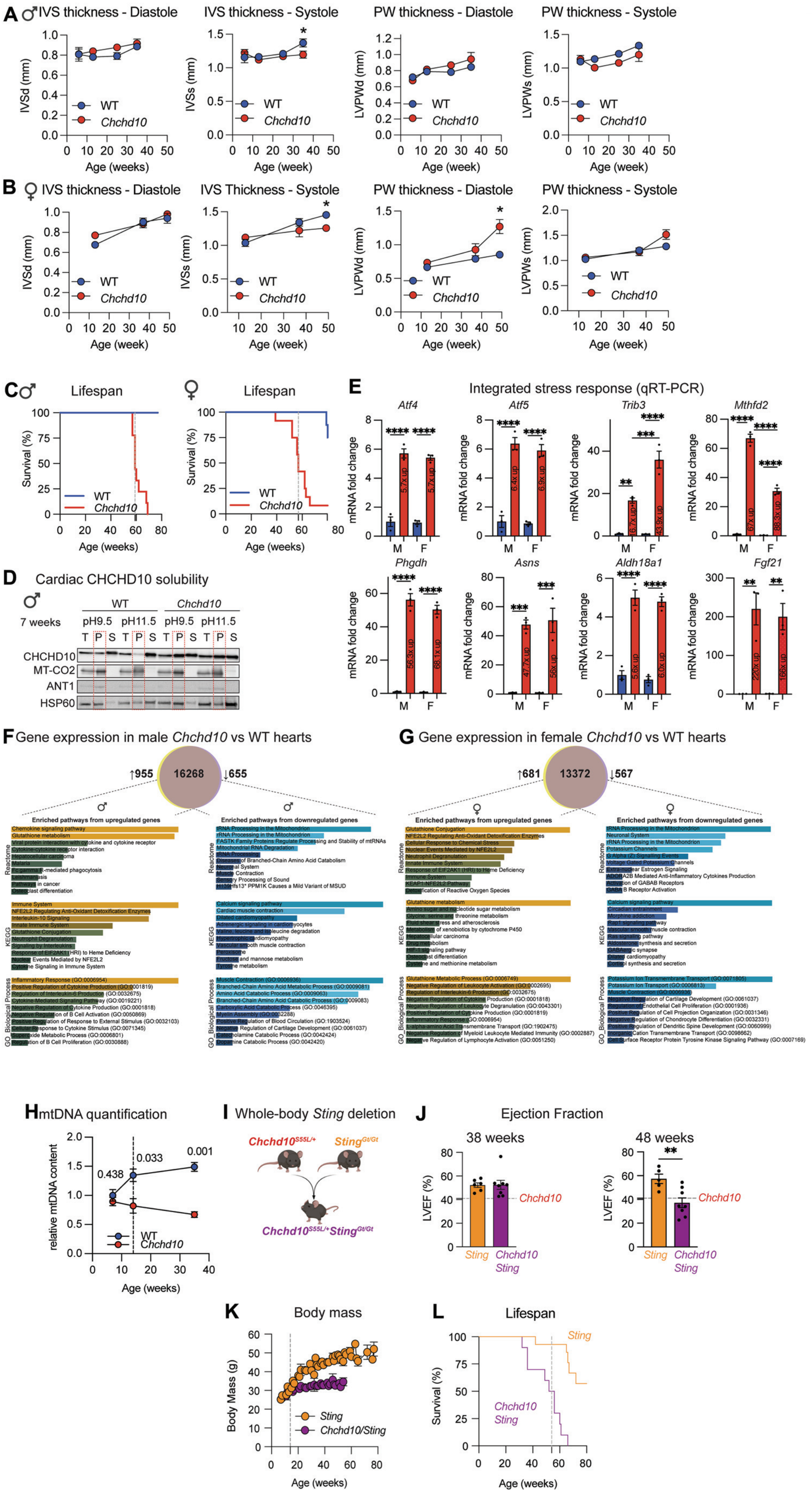
A ♂ IVS thickness - Diastole
IVS thickness - Systole
PW thickness - Diastole
PW thickness - Systole
B ♀ IVS thickness - Diastole
IVS Thickness - Systole
PW thickness - Diastole
PW Thickness - Systole
IVSd (mm)
IVSs (mm)
LVPWd (mm)
LVPWs (mm)
WT
Chchd10
Age (weeks)
Age (week)
C ♂ Lifespan
♀ Lifespan
Survival (%)
E Integrated stress response (qRT-PCR)
Atf4
Atf5
Trib3
Mthfd2
Phgdh
Asns
Aldh18a1
Fgf21
mRNA fold change
M F
D Cardiac CHCHD10 solubility
7 weeks
pH9.5 pH11.5
T P S
CHCHD10
MT-CO2
ANT1
HSP60
F Gene expression in male Chchd10 vs WT hearts
↑955 16268 ↓655
G Gene expression in female Chchd10 vs WT hearts
↑681 13372 ↓567
Enriched pathways from upregulated genes
Enriched pathways from downregulated genes
Reactome
KEGG
GO_Biological Process
H mtDNA quantification
relative mtDNA content
0.438
0.033
0.001
I Whole-body Sting deletion
Chchd10^S55L/+
Sting^Gt/Gt
Chchd10^S55L/+Sting^Gt/Gt
J Ejection Fraction
38 weeks
48 weeks
LVEF (%)
Sting
Chchd10 Sting
K Body mass
Body Mass (g)
Chchd10/Sting
L Lifespan
Age (weeks)

 © The Author(s)

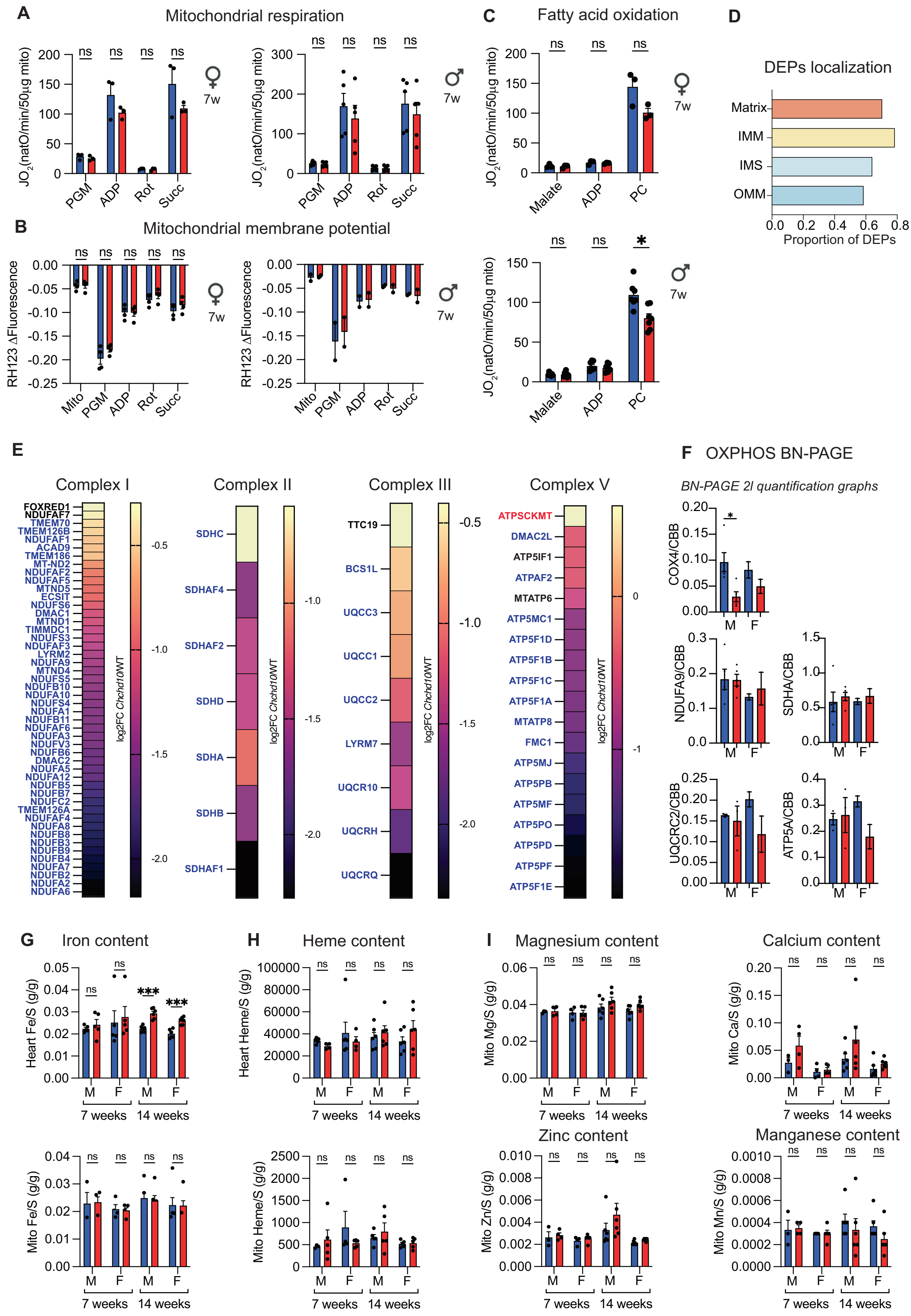
A
Mitochondrial respiration
B
Mitochondrial membrane potential
C
Fatty acid oxidation
D
DEPs localization
Matrix
IMM
IMS
OMM
Proportion of DEPs
E
Complex I
Complex II
Complex III
Complex V
log2FC Chchd10/WT
F
OXPHOS BN-PAGE
BN-PAGE 2I quantification graphs
G
Iron content
H
Heme content
I
Magnesium content
Calcium content
Zinc content
Manganese content
7 weeks
14 weeks

Figure EV2. Impaired mitochondrial respiration in mutant *Chchd10* hearts.

(**A**) Oxygen consumption rates (JO_2) of cardiac mitochondria isolated from WT ($n = 3$–5) and *Chchd10* ($n = 3$–5) female (left) and male (right) mice at 7 weeks. JO_2 measured sequentially in the presence of pyruvate, glutamate, malate (PGM), adenosine diphosphate (ADP), rotenone (Rot), and succinate (Succ). Data represent mean ± SEM; multiple unpaired *t* test, ns=not significant. (**B**) Mitochondrial membrane potential (ΔΨ) measured by quenching of Rhodamine 123 (RH123) fluorescence in cardiac mitochondria of WT and *Chchd10* male ($n = 5$) and female ($n = 2$) mice from Fig. EV2A. Data represent mean ± SEM; multiple unpaired *t* test, ns=not significant. (**C**) Oxygen consumption rates (JO_2) of cardiac mitochondria isolated from WT ($n = 3$–7) and *Chchd10* ($n = 3$–7) female (top) and male (bottom) mice at 7 weeks. JO_2 measured sequentially in the presence of malate, adenosine diphosphate (ADP), and palmitoyl carnitine (PC). Data represent mean ± SEM; multiple unpaired *t* test, *$P = 0.003882$, ns=not significant. (**D**) Mitochondrial localization of DEPs in Fig. 2G according to MitoCarta 3.0 represented as proportion of total quantified DEPs in each mitochondrion subcompartment. (**E**) Heatmap of Complex I, II, III, and V proteins quantified by proteomics in Fig. 2G and significantly upregulated (red), downregulated (blue) or unchanged (black) between WT ($n = 5$) and *Chchd10* ($n = 5$) male mice at 14 weeks (Dataset EV5). (**F**) Densitometric quantification of OXPHOS complexes in Fig. 2L is relative to Coomassie brilliant blue (CBB). Data are means ± SEM, one-way ANOVA. *$P = 0.0252$. (**G**) Iron content in total heart (top, $n = 5$–6) and cardiac mitochondria (bottom, $n = 3$–6) samples measured by inductively coupled plasma–optical emission spectrometry (ICP-OES) from WT and *Chchd10* male (M) and female (F) mice at 7 and 14 weeks of age. Data represent mean values normalized to sulfur (S) ± SEM, multiple unpaired *t* test, 14 weeks M; WT vs *Chchd10*, ***$P < 0.000112$, 14 weeks F; WT vs *Chchd10*, ***$P < 0.000233$, ns=not significant. (**H**) Heme content in total heart (top, $n = 5$–6) and cardiac mitochondria (bottom, $n = 3$–6) samples measured by HPLC from WT and *Chchd10* male (M) and female (F) mice at 7 and 14 weeks of age. Data represent mean values normalized to sulfur (S) ± SEM, multiple unpaired *t* test, ns=not significant. (**I**) Magnesium, Zinc, Calcium, and Manganese content in cardiac mitochondria measured by inductively coupled plasma–optical emission spectrometry (ICP-OES) from WT and *Chchd10* male (M) and female (F) mice at 7 and 14 weeks of age. Data represent mean values normalized to sulfur (S) ± SEM, multiple unpaired *t* test, ns=not significant. Source data are available online for this figure.

 © The Author(s)

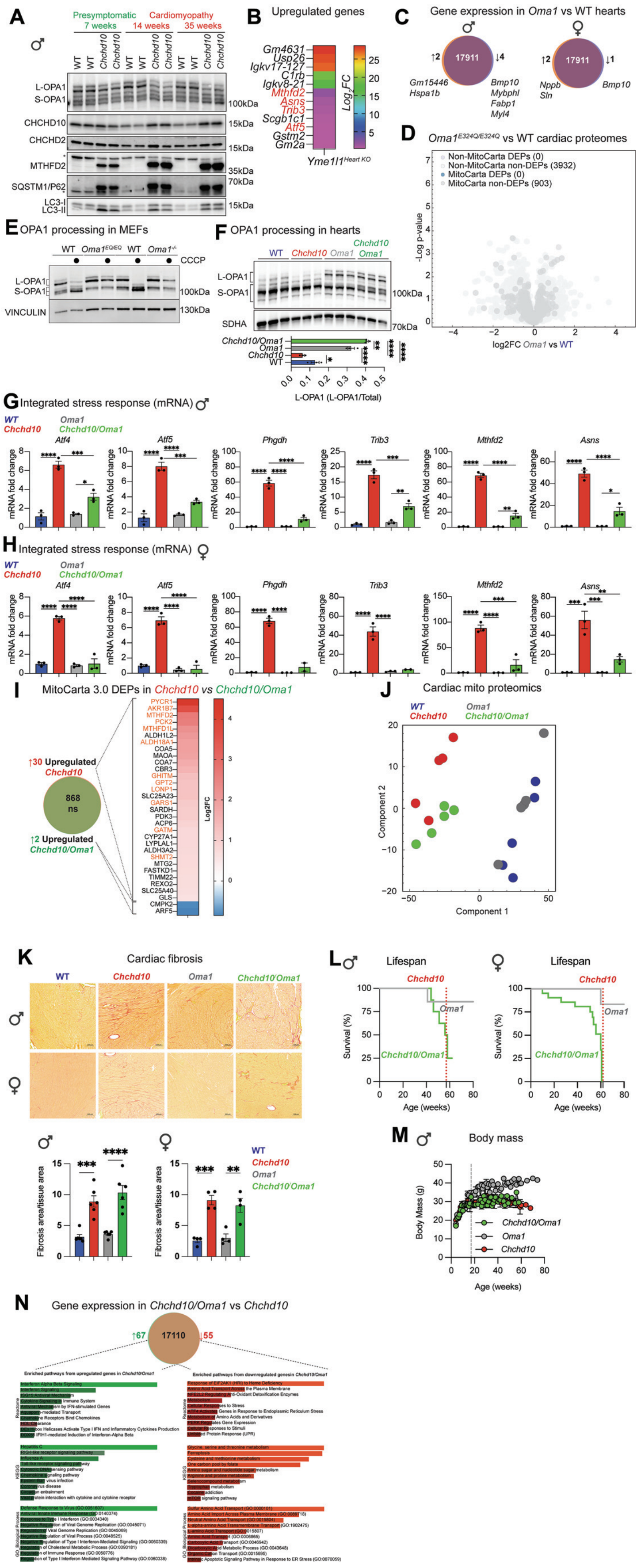
A
Presymptomatic 7 weeks
Cardiomyopathy 14 weeks 35 weeks
L-OPA1
S-OPA1
CHCHD10
CHCHD2
MTHFD2
SQSTM1/P62
LC3-I
LC3-II
B Upregulated genes
Yme1l1 Heart KO
C Gene expression in Oma1 vs WT hearts
D Oma1 E324Q/E324Q vs WT cardiac proteomes
E OPA1 processing in MEFs
F OPA1 processing in hearts
G Integrated stress response (mRNA)
H Integrated stress response (mRNA)
I MitoCarta 3.0 DEPs in Chchd10 vs Chchd10/Oma1
J Cardiac mito proteomics
K Cardiac fibrosis
L Lifespan
M Body mass
N Gene expression in Chchd10/Oma1 vs Chchd10

Figure EV3. OMA1 and mtISR activation.

(**A**) Immunoblots in cardiac lysates of presymptomatic (green) and symptomatic (red) WT and *Chchd10* mice. (**B**) Heatmap of upregulated differentially expressed genes (DEGs) in cardiomyocyte-specific *Yme1l* knockout mice (*Yme1l1*^{HeartKO}). Bulk RNA-seq was performed on cardiac biopsies from male ($n = 3$) mice compared to sex-matched littermate controls at 35 weeks of age. Integrated stress response (ISR) genes are highlighted red. (**C**) Venn diagram of differentially expressed genes (DEGs) in *Oma1* mice. Bulk RNA-seq was performed on cardiac biopsies from male ($n = 3$) and female ($n = 3$) *Oma1* mice compared to sex-matched WT controls at 14 weeks of age. DEG is defined by Log2FC > 2 and padj <0.01 (Dataset EV1). (**D**) Volcano plot of cardiac proteomics WT vs Oma1 No differentially expressed proteins (DEPs) were identified. Two-sided unpaired *t* test followed by permutation-based FDR correction. Significance is considered a permutation-based FDR at 0.05 highlighted by color. (**E**) Immunoblots of OPA1 processing in mouse embryonic fibroblasts (MEFs) derived from wild-type (WT) and *Oma1*$^{E324Q/E324Q}$ (*Oma1*$^{EQ/EQ}$) mice. Carbonyl cyanide m-chlorophenyl hydrazine (CCCP) used to induce stress-induced L-OPA1 processing. WT and OMA1 knockout (*Oma1*$^{-/-}$) MEFs were used as a control (Wai et al, 2015). (**F**) (Top) Immunoblot of L-OPA1 and S-OPA1 in cardiac mitochondria of WT (blue, $n = 3$), *Chchd10* (red, $n = 3$), *Oma1* (gray, $n = 3$), and *Chchd10/Oma1* (green, $n = 3$) mice between 17 and 19 weeks. SDHA used as loading control(Bottom) Densitometric quantification of L-OPA1/Total OPA1. Data are mean ± SEM, One-way ANOVA, *$P < 0.05$, **$P < 0.01$, ****$P < 0.0001$. (**G**) Analysis of integrated stress response (ISR) genes via qRT-PCR of total cardiac biopsies from wild-type (WT, blue, $n = 3$), *Chchd10* (red, $n = 3$), *Oma1* (gray, $n = 3$), and *Chchd10/Oma1* (green, $n = 3$) male mice at 14 weeks in Fig. 3C. Data are relative mean fold changes ± SEM, one-way ANOVA, *Atf4*; *$P = 0.259$, ***$P < 0.0005$,****$P < 0.0001$, *Atf5*; ***$P = 0.0001$,****$P < 0.0001$, *Phgdh*; ****$P < 0.0001$, *Trib3*; **$P = 0.0098$, ***$P = 0.0001$, ****$P < 0.0001$, *Mthfd2*; **$P = 0.0071$, ****$P < 0.0001$, *Asns*; *$P = 0.0188$, ****$P < 0.0001$. (**H**) Analysis of integrated stress response (ISR) genes via qRT-PCR of total cardiac biopsies from wild-type (WT, blue, $n = 3$), *Chchd10* (red, $n = 3$), *Oma1* (gray, $n = 3$), and *Chchd10/Oma1* (green, $n = 2$–3) female mice at 14 weeks in Fig. 3C. Data are relative mean fold changes ± SEM, one-way ANOVA, *Atf4*; ****$P < 0.0001$, *Atf5*; ****$P < 0.0001$, *Phgdh*; ****$P < 0.0001$, *Trib3*; ****$P < 0.0001$, *Mthfd2*; ***$P = 0.0001$, ****$P < 0.0001$, *Asns*; **$P = 0.0018$, ***$P = 0.0002$. (**I**) Heatmap of differentially expressed proteins (DEPs) upregulated in cardiac mitochondria profiled from *Chchd10* (red, $n = 5$) versus *Chchd10/Oma1* (green, $n = 5$) hearts by mass spectrometry (Dataset EV5). (**J**) Principal component analysis (PCA) of cardiac proteomics performed on wild-type (WT, $n = 5$, blue), *Chchd10* ($n = 5$, red), *Oma1* ($n = 5$, gray) and *Chchd10/Oma1* ($n = 5$, green) male mice at 14 weeks of age (Dataset EV5). (**K**) Cardiac histology of 4 genotypes and both sexes. Representative Sirius red myocardium staining of wild-type (WT), *Chchd10*, *Oma1*, and *Chchd10/Oma1* male (22 weeks of age, $n = 6$) and female (22 weeks of age, $n = 4$) mice. Tissue fibrosis analysis was performed using an automated macro developed in QuPath and quantified (bottom). Data represent mean ± SEM. One-way ANOVA, Male; WT vs *Chchd10* ***$P = 0.0002$, *Oma1* vs *Chchd10/Oma1* ****$P < 0.0001$, Female; WT vs *Chchd10* ***$P = 0.0003$, *Oma1* vs *Chchd10/Oma1* **$P < 0.0019$, ns=not significant. (**L**) Kaplan–Meier survival curve (left) of *Oma1* (gray, $n = 15$), and *Chchd10/Oma1* (green, $n = 15$) male mice and (right) *Oma1* (gray, $n = 13$), and *Chchd10/Oma1* (green, $n = 21$) female mice. Dotted red line represents median lifespan of *Chchd10* mice. Log-rank test *Chchd10* vs *Chchd10/Oma1*; male $P = 0.0844$ and female $P = 0.2520$. (**M**) Body mass of *Oma1* (gray), and *Chchd10/Oma1* (green) male mice compared to data from male *Chchd10* mice. Dotted gray line represents the age after which body mass differences between WT and *Chchd10* male (17 weeks) mice are observed. (**N**) Venn diagram of differentially expressed genes (DEGs) in female *Chchd10/Oma1* versus *Chchd10* mice. Bulk RNA-seq was performed on cardiac biopsies ($n = 3$) at 14 weeks of age. Reactome, KEGG, and Gene Ontology (GO) pathway enrichment performed with Enrichr. Source data are available online for this figure.

 © The Author(s)

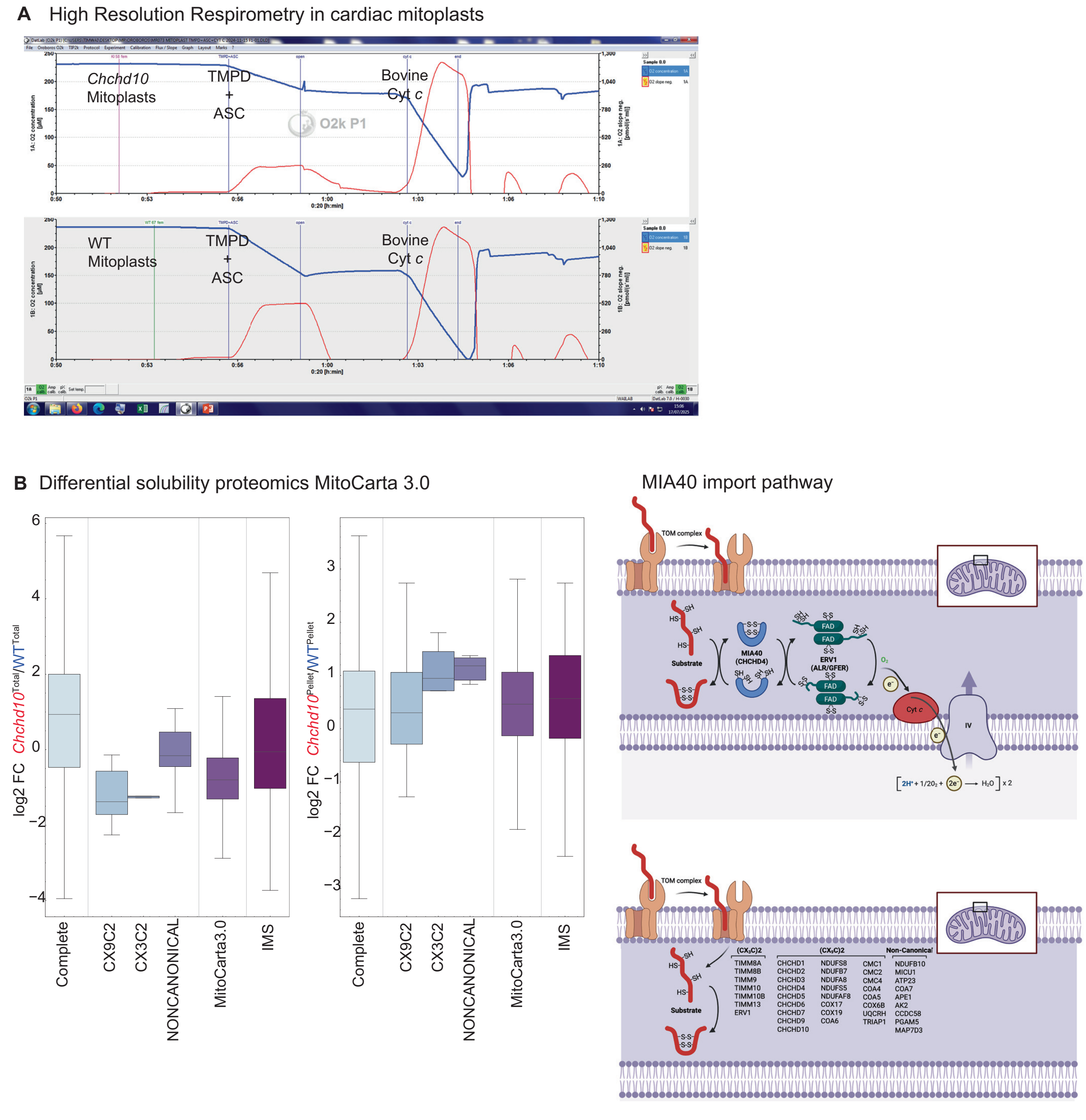

Figure EV4. Differential solubility proteomics in *Chchd10* mice.

(**A**) Representative traces of oxygen consumption rates (JO_2) measured in *Chchd10* and wild-type (WT) mitoplasts using High-Resolution Respirometry (Oroboros). Blue trace represents chamber oxygen (O_2) concentration (µM, left *y* axis) and red trace represents O_2 consumption (pmol/(s*ml, right *y* axis). JO_2 measured sequentially following addition of N,N,N′,N′-Tetramethyl-p-phenylenediamine (TMPD) plus ascorbate (ASC) and bovine cytochrome *c* (Cyt *c*) at indicated times (running time in hours: min (h:min), *x* axis). (**B**) Boxplots of MitoCarta, IMS, and MIA40 client types based on differential solubility proteomic analyses of total (T, left) and pellet (P, right) fractions of detergent-solubilized cardiac mitochondria from WT ($n = 5$) and *Chchd10* ($n = 5$) male mice at 14 weeks of age (left, Dataset EV7). Box edges display the 25% (lower) and 75% (upper) quartile. The whiskers display the highest and lowest non-outlier value. Outliers (1.5 x inter quartile range from box edge) are not shown. The black line in the box indicates the median (50%). Cartoon representation of MIA40/ERV1 import pathway (top) in which client proteins (Al-Habib and Ashcroft, 2021) are indicated (right).
